# From detail to diversity: Capturing the chemical signature of non-*Saccharomyces* yeasts in white wine through GC×GC/TOF-MS metabolomics and complementary analytical approaches^☆^

**DOI:** 10.1016/j.fochx.2026.103789

**Published:** 2026-03-24

**Authors:** Doris Delač Salopek, Urska Vrhovsek, Silvia Carlin, Sanja Radeka, Marina Tomašević, Igor Lukić

**Affiliations:** a Institute of Agriculture and Tourism, Karla Huguesa 8, 52440 Poreč, Croatia; b Metabolomics Unit, Research and Innovation Centre, Fondazione Edmund Mach (FEM), Via Edmund Mach 1, 38098 San Michele all'Adige, Italy; c Faculty of Food Technology and Biotechnology, University of Zagreb, Pierottijeva 6, 10000 Zagreb, Croatia

**Keywords:** Wine, Volatiles, GC×GC/TOF-MS, Metschnikowia pulcherrima, Pichia kluyveri, Lachancea thermotolerans, Schizosaccharomyces pombe

## Abstract

To gain a broad understanding of yeast species' effects, Malvazija istarska wines produced by sequential inoculation with five non-*Saccharomyces* starters and monoculture fermentation with a *Saccharomyces cerevisiae×S. paradoxus* hybrid and a *S. cerevisiae* control were thoroughly analyzed. Two-dimensional gas chromatography/mass spectrometry, alongside conventional GC, enabled the identification of 399 volatile compounds and revealed many yeast-specific effects. Non-*Saccharomyces* starters generally decreased the concentrations of acetaldehyde, 2-phenylethanol, fatty acids, and volatile phenols, while increasing the concentrations of isobutanol, its esters, and isoamyl acetate. *Torulaspora delbrueckii* had the most pronounced impact, with higher concentrations of short-chain ethyl esters and acetates, and lower levels of acetaldehyde, medium-chain acids, and their ethyl esters. The effects on terpenoids, norisoprenoids, thiols, C_6_-alcohols, ketones, lactones, and furanoids varied. Multivariate analysis revealed numerous yeast-specific volatile markers. Non-*Saccharomyces* yeasts preserved more hydroxycinnamic acids. Overall, the results obtained provided an in-depth insight into the yeast-driven modulation of white wine chemical composition.

## Highlights

• GC/MS and GC × GC/TOF-MS resulted in the identification of 399 volatile compounds.
• Each yeast excelled by producing higher/lower concentration of many specific markers.
• Non-*Saccharomyces* yeasts: decreased acetaldehyde, vinylphenols, increased isobutanol derivatives, isoamyl acetate.
• *T. delbrueckii* as a starter exhibited the most distinctive ester composition.
• Comprehensive insight into the effects of various yeasts in white wine production.

## 1 Introduction

Non-*Saccharomyces* yeasts have traditionally been associated with negative impacts on wine quality. However, in recent decades, studies have shown that when used as co-fermentation starters with conventional *Saccharomyces cerevisiae* strains, they can enhance physico-chemical characteristics and contribute to greater complexity and stylistic uniqueness in wine. Consequently, several species are now commercially available and used in modern winemaking (Morata et al., 2020). Due to their high sensitivity to sulfur dioxide and alcohol, and limited fermentation capacity, non-*Saccharomyces* yeasts require sequential inoculation or co-inoculation with *S. cerevisiae* to ensure complete fermentation. This approach provides enough time for non-*Saccharomyces* yeasts to influence wine composition, while *S. cerevisiae* completes the fermentation (Lleixà et al., 2016; Maicas & Mateo, 2023). Among their various applications, certain species like *Schizosaccharomyces pombe* and *Metschnikowia pulcherrima* have been shown to reduce ethanol levels in wine, helping to mitigate the effects climate change, including premature grape ripening (Blanco et al., 2020; Morata et al., 2020). *Schizosaccharomyces pombe* naturally lowers total acidity by partially converting malic acid to ethanol, potentially eliminating the need for malolactic bacteria and their complex metabolic pathways and associated risks (Benito, 2019; Vicente et al., 2023). In contrast, *Lachancea thermotolerans* can produce significant amounts of lactic acid and be used for bio-acidification (Blanco et al., 2020). *Torulaspora delbrueckii* and *M. pulcherrima* have been found to show strong bioprotective effects by limiting undesirable microbiota and reducing the need for sulfur dioxide (Escribano-Viana et al., 2022).

Besides the mentioned effects, non-*Saccharomyces* yeasts can also significantly influence the volatile aroma profile of wine, enhancing its complexity and distinctiveness. Their aromatic contributions vary among species and strains, likely due to differences in the activity of enzymes encoded by key genes, most of which have been characterized primarily in *S. cerevisiae*. These include: glycosidases (e.g., β-glucosidases) which contribute to the liberation of glycosylated varietal aroma compounds; β-lyases involved in volatile thiol release (genes *IRC7*, *STR3*); Ehrlich-pathway enzymes shaping higher alcohols and derived esters (e.g., decarboxylases; *PDC1*, *PDC5*, and *PDC6*); alcohol dehydrogenases influencing higher alcohol/ethanol interconversions (*ADH* gene family); acyl-CoA:ethanol O-acyltransferases responsible for medium-chain ethyl ester formation (*EEB1*, *EHT1*); alcohol acetyltransferases driving acetate ester formation (*ATF1*, *ATF2*); esterases/lipases affecting ester turnover (e.g., *IAH1* and others); and sulfur assimilation/metabolism enzymes affecting sulfur-containing volatiles (e.g., *MET* pathway genes) (Dzialo et al., 2017; Hazelwood et al., 2008; Padilla et al., 2016; Roncoroni et al., 2011; Saerens et al., 2010; Swiegers & Pretorius, 2007). Several studies have investigated the use of non-*Saccharomyces* starters to modulate wine volatile composition under various conditions and inoculation regimes (Anfang et al., 2009; Benito et al., 2015; Belda, Ruiz, Beisert, et al., 2017; Binati et al., 2023; Canonico et al., 2023; Cheng et al., 2025; Dutraive et al., 2019; Del Fresno et al., 2025; Maicas & Mateo, 2023; Renault et al., 2016; Sadoudi et al., 2017; Vicente et al., 2021). In such studies, conventional gas chromatography (GC) coupled with mass spectrometry (MS), commonly referred to as GC/MS, has been widely used for the (tentative) identification and quantitative analysis of a limited number of target compounds. In this regard, the role of GC/MS remains undeniable. However, in recent years, comprehensive two-dimensional gas chromatography (GC × GC) has emerged as a state-of-the-art technique for analyzing complex mixtures of volatile compounds in matrices such as wine (Zhang et al., 2023). The additional column in GC × GC allows for superior separation, lower spectral interference, and more detailed insights through a significantly larger number of identified analytes (Carlin et al., 2016; Lukić et al., 2020). Compared to conventional GC/MS, GC × GC represents a major advancement, offering peak capacities that are up to ten times greater. This suggests that it could be exploited to substantially broaden the scope of information available in non-*Saccharomyces* fermentation studies, potentially uncovering numerous previously undetected effects of enological relevance. To date, the application of advanced GC × GC techniques in this context remains rare (Beckner Whitener et al., 2016; Delač Salopek et al., 2024). Furthermore, the impact of non-*Saccharomyces* fermentation on other essential wine constituents, particularly phenolics, which are key to oxidative stability, bitterness, astringency, and color, remains poorly characterized. Certain yeast species have been shown to influence these compounds, but empirical data on their modification via non-*Saccharomyces* co-fermentation remain notably limited (Zhang et al., 2021).

This study hypothesizes that the biochemical effects of non-*Saccharomyces* starters, now widely employed in modern winemaking, extend well beyond current understanding. The primary objective was to comprehensively examine their influence across a much broader spectrum of wine-relevant metabolites, including both volatiles and phenolics. To achieve this, fermentations were performed using a diverse panel of non-*Saccharomyces* species, alongside a *S. cerevisiae* × *S. paradoxus* hybrid and a *S. cerevisiae* control, while the analysis of volatiles combined traditional GC/MS with untargeted comprehensive GC × GC coupled with time-of-flight mass spectrometry (GC × GC/TOF-MS). Simultaneously, the study addressed the often-overlooked modulation of phenolic composition by yeast activity during fermentation. The experimental design, which involved multiple yeast species tested under identical vinification conditions, enabled a robust comparative analysis that is hardly attainable by cross-study comparisons, primarily due to variations in grape material and fermentation protocols. The results are expected to significantly enhance understanding in this research area and offer practical value for the wine industry.

## 2 Materials and methods

### 2.1 Experiment set-up

#### 2.1.1 Grape processing

The experiment was conducted using grapes of Malvazija istarska (*Vitis vinifera* L.), the most widespread and economically important native white cultivar in Croatia, harvested on 28 September 2021 from the experimental vineyard of the Institute of Agriculture and Tourism in Poreč (Istria, Croatia). Winery equipment was sanitized with caustic soda, potassium metabisulfite, citric acid, and 70% (v/v) ethanol, followed by rinsing with hot water. Handpicked grapes were destemmed, crushed, and pressed using a closed-type pneumatic press (500 L capacity, Letina Inox d.o.o., Čakovec, Croatia) at pressures of 2 × 0.5 bar and 1 × 0.8 bar. Juice was sulfited with potassium metabisulfite (8 g/hL; Corimpex Service S.R.L., Romans d'Isonzo, Italy) and cold-settled with Endozym Rapid pectolytic enzymes (2 g/hL; AEB s.p.a., Brescia, Italy) for 36 h at 10 °C. Before acidification, the juice had 22.1°Brix, 115 mg/L YAN, total acidity of 4.7 g/L (as tartaric acid), and pH 3.41; the latter two parameters were adjusted to 6.0 g/L and 3.27, respectively, by the addition of tartaric acid. The homogenized juice was distributed into 80 L stainless steel tanks.

#### 2.1.2 Fermentation

The selected non-*Saccharomyces* yeasts and strains were chosen based on their documented enological relevance, commercial availability, and previously reported impacts on fermentation performance and aroma modulation. Together, they represent functionally distinct groups with well-established enological significance rather than providing an exhaustive coverage of non-*Saccharomyces* diversity (Vejarano & Gil-Calderón, 2021). The non-*Saccharomyces* yeasts used were *T. delbrueckii* (TD; BIODIVA®), *M. pulcherrima* (MP; FLAVIA®), and *L.* *thermotolerans* (LT; LAKTIA®) from Lallemand Inc. (Montreal, Canada), *S. pombe* (SP; Atecrem 12H®, BioEnologia 2.0, Oderzo, Italy), and *Pichia kluyveri* (PK; Frootzen®, CHR Hansen, Hoersholm, Denmark). Additionally, a *S. cerevisiae × S. paradoxus* hybrid (SC × SPx; EXOTIC®, Oenobrands, Montpellier, France) and a *S. cerevisiae* control (SC; Lalvin EC1118®, Lallemand Inc.) were used in monoculture. The same *S. cerevisiae* strain (SC) was used to complete the fermentations initiated by non-*Saccharomyces* yeasts (TD, MP, LT, SP, and PK) and was inoculated once the volume fraction of ethanol reached approximately 2% (v/v), as calculated from the measured °Brix values in the fermenting grape musts. SC, SC × SPx, TD, MP, and LT yeasts were rehydrated, SP was inoculated in cream form, and frozen PK was thawed before direct inoculation. Yeasts were inoculated at ∼4–5 × 10^6^ cells/mL, except PK, which was used at 1 × 10^6^ cells/mL (a tenfold higher concentration than that recommended by the producers) to reasonably match the initial concentrations of the other yeasts. Specific abbreviations were assigned to the treatments to clearly denote sequential inoculations: *T. delbrueckii* and *S. cerevisiae* (TD + SC), *M. pulcherrima* and *S. cerevisiae* (MP + SC), *P. kluyveri* and *S. cerevisiae* (PK + SC), *L. thermotolerans* and *S. cerevisiae* (LT + SC), and *S. pombe* and *S. cerevisiae* (SP + SC).

Fermentations were conducted at 17 °C in triplicate. Diammonium phosphate (30 g/hL; Corimpex Service S.R.L.) was added 36 h post-inoculation. The density of the fermenting musts, converted into °Brix values, was monitored daily using a DMA 35 portable density meter (Anton Paar GmbH, Graz, Austria), providing an approximate estimate of sugar concentration during fermentation. In the later stages of fermentation, reducing sugars were determined daily by the OIV standard method (OIV, 2022). Once fermentation was completed (residual reducing sugars <4.0 g/L), wines were racked, settled for 3 weeks, racked again, and sampled for analysis. The wines were sulfited at all critical stages of vinification, specifically during the first and second rackings, to achieve a target free SO₂ concentration of 35 mg/L. The analyses were conducted six months post-fermentation, during which the wines were stored in the cellar under appropriate conditions.

### 2.2 Physico-chemical analysis

#### 2.2.1 Standard physico-chemical parameters, organic acids and glycerol

Alcoholic strength, total dry extract, reducing sugars, total and volatile acidity, and pH were determined by OIV methods (OIV, 2022). Organic acids and glycerol were analyzed by HPLC using an Agilent Infinity 1260 system (Agilent Technologies, Santa Clara, CA, USA) equipped with DAD and RID detectors for determination of organic acids and glycerol, respectively. Samples (0.5 mL) were diluted with 1.0 mL ultrapure water, filtered (0.45 μm PTFE), and injected (10 μL) onto an Agilent Hi-Plex H column (300 × 7.7 mm, particle size 8 μm) with a PL Hi-Plex H guard (5 × 3 mm) (Agilent Technologies). The eluent was 4 mM sulfuric acid, flow rate 0.5 mL/min, at 70 °C. UV/Vis detection was at 210 nm, and acids were identified by comparing their retention times and spectra with those of pure standards. The RID cell was held at 50 °C. Calibration curves were built from standards in 13% ethanol at pH 3.3.

#### 2.2.2 Major volatile aroma compounds

GC/FID with direct injection was used to analyze acetaldehyde, ethyl acetate, methanol, and higher alcohols, using a Varian 3350 GC (Varian Inc., Harbour City, CA, USA) with an Rtx-WAX column (60 m × 0.25 mm i.d. × 0.25 μm d.f.; Restek, Bellefonte, PA, USA). A split ratio of 1:20 was applied. 1-Pentanol was used as an internal standard. Calibration curves for quantification were generated from pure standard solutions by plotting peak area ratios relative to the internal standard against concentration. Data processing was performed using Varian Star software (version 4.51; Varian Inc.).

Other major volatiles were extracted by HS-SPME using a DVB/CAR/PDMS fiber (StableFlex, 50/30 μm, 1 cm; Supelco, Bellefonte, PA, USA), and analyzed by GC/MS (Varian 3900 GC and Saturn 2100 T MS; Varian Inc.) with the same column. An internal standard mixture consisting of 2-octanol (for all compounds except fatty acids) and heptanoic acid (for fatty acids) was used. Calibration curves were generated from pure standard solutions by plotting peak area ratios relative to the internal standard against concentration. Compounds for which commercial standards were not available were semi-quantified by assuming equivalent detector response for structurally similar compounds. Data processing was performed using Varian MS Workstation (version 6.6; Varian Inc.). The operating conditions, as well as the identification, quantification and validation parameters, were as described by Lukić et al. (2020).

#### 2.2.3 Minor volatile compounds

Minor volatiles were extracted by HS-SPME using a DVB-CAR-PDMS fiber (StableFlex, 50/30 μm, 2 cm; Supelco, Sigma Aldrich, Milan, Italy). Injections in splitless mode were performed with a Gerstel MPS autosampler (Gerstel GmbH & Co. KG, Mülheim an der Ruhr, Germany), and the analysis was carried out by GC × GC/TOF-MS using an Agilent 7890 N GC (Agilent Technologies) connected to a LECO Pegasus IV time-of-flight MS (TOF-MS) (Leco Corporation, St. Joseph, MI, USA). The system included two columns of differing polarity connected by a modulator. The first-dimension column (30 m × 0.25 mm × 0.25 μm d.f. VF-WAXms; Agilent Technologies) was held at 40 °C for 4 min, ramped to 250 °C at 6 °C/min, then held for 5 min. The second-dimension column (1.5 m × 0.15 mm × 0.15 μm d.f. Rxi 17Sil MS; Restek) operated at 5 °C above the temperature of the first column during the entire analysis. Helium flow was set at 1.2 mL/min. Mass spectra were acquired in EI mode (70 eV, 40–350 *m*/*z*). Data were processed using LECO ChromaTOF software version 4.32 (Leco Corporation) for baseline correction, chromatogram deconvolution, and peak alignment. Baseline offset was set to 0.8 and signal-to-noise ratio (S/N) was set to 100. A mixture of pure standards of 122 volatile compounds was analyzed under the same GC × GC/TOF-MS conditions for identification purposes. Volatile compounds were identified by comparison of their retention times and mass spectra with those of pure standards and with mass spectra from commercial mass spectral libraries (NIST 2.0, Wiley 8, and FFNSC 2 (Chromaleont, Messina, Italy)), using a minimum similarity match factor of 750 as the acceptance criterion. To further confirm compound identities, experimentally determined linear retention indices (calculated relative to C10–C30 n-alkanes) were compared with literature values obtained from one-dimensional GC analyses using equivalent or similar capillary columns. Concentrations of individual volatile compounds (μg/L) were semi-quantified relative to the internal standard 2-octanol, under the assumption of equivalent detector responses. The operating conditions, as well as the identification and quantification parameters, have been reported previously in Carlin et al. (2016) and Lukić et al. (2020).

#### 2.2.4 Volatile thiols

Volatile thiols were analyzed according to Tominaga et al. (1998), with modifications described by Tomašević et al. (2017). Wine (50 mL) was treated with 5 mL of 1 mM *p*-hydroxymercurybenzoate, equilibrated, and loaded onto a Dowex 1 × 2 chloride form column (Sigma Aldrich, St. Louis, MO, USA). Thiols were eluted with a solution of cysteine hydrochloride monohydrate, extracted with 2 mL of dichloromethane, and, after desiccation, analyzed by GC/MS (Agilent 6890 GC with 5973 MS; Agilent Technologies). Extracts were injected onto a BP20 column (50 m × 0.22 mm × 0.25 μm d.f.; SGE Analytical Science, Victoria, Australia) in splitless mode via a 7683B autosampler (Agilent Technologies). Oven temperature was 40 °C for 5 min, then increased to 200 °C at 3 °C/min, and subsequently to 240 °C at 30 °C/min, with a final hold of 1 min. Selective Ion Monitoring (SIM) mode was used. The remaining operation conditions, as well as the identification and quantification parameters, have been reported previously in Tomašević et al. (2017).

#### 2.2.5 Phenolic compounds

Phenolics were analyzed by UPLC/QqQ-MS/MS using an Acquity UPLC system coupled to a Xevo TQ MS (Waters Corporation, Milford, MA, USA), according to the method described by Vrhovsek et al. (2012). Samples were filtered through a 0.2 μm PTFE filter and injected onto a reverse-phase Acquity HSS T3 column (100 mm × 2.1 mm, 1.8 μm; Waters Corporation). Mobile phases consisted of water and acetonitrile, both with 0.1% formic acid. Multistep gradients, MRM conditions, and quantification details followed Vrhovsek et al. (2012). Data were processed using MassLynx 4.1 and TargetLynx 4.1 software (Waters Corporation).

Total phenolics were measured by the Folin–Ciocalteu assay with a Cary 50 UV/Vis spectrophotometer (Varian Inc.) at 765 nm, and the results were expressed as mg/L gallic acid equivalents (GAE).

### 2.3 Statistical analyis

One-way analysis of variance (ANOVA) and the Least Significant Difference (LSD) test (*p* < 0.05) were used to assess differences among treatments. Hierarchical clustering analysis (HCA) was performed on two normalized datasets: one comprising 50 volatile compounds and the other comprising 70 esters, selected as those with the highest *F*-ratios from ANOVA (clustering: Ward method; distance: Euclidean). Forward stepwise linear discriminant analysis (SLDA) was separately conducted on volatile and phenolic compounds, with Wilks' lambda as the entry criterion (*F*-to-enter = 1). ANOVA and SLDA were performed using Statistica v. 13.2 (StatSoft Inc., Tulsa, OK, USA), and HCA was performed using MetaboAnalyst v. 6.0 software (http://www.metaboanalyst.ca).

## 3 Results and discussion

### 3.1 Standard physico-chemical parameters

Previous studies reported reduced alcohol content after sequential fermentation with particular non-*Saccharomyces* yeasts (Blanco et al., 2020; Morata et al., 2020), but no significant differences in ethanol concentration were found among the wines in this study (Table 1). SP + SC wine had the lowest dry extract without reducing sugars and the lowest total acidity, mostly due to reduced malic acid, confirming *S. pombe*'s capacity to convert malic acid to ethanol via malic dehydrogenases (Vicente et al., 2023). Such a compositional profile could potentially be reflected in a less intense perception of acidity. LT + SC and TD + SC wines had increased lactic acid levels (Table 1), consistent with previous *L.* *thermotolerans* research (Benito, 2018). Benito (2018) reported substantial variability in lactic acid production by *L.* *thermotolerans* strains across studies, influenced by inoculation modality, with values spanning from 0.3 to 9.6 g/L. The 12.5% increase in LT + SC compared to SC wine observed in this study (Table 1) was modest, aligning with results for some strains, whereas most studies reported substantially higher increases in lactic acid concentration (Binati et al., 2020; Hranilović et al., 2021, Hranilović et al., 2022). In the present study, a selected commercial *L.* *thermotolerans* strain with documented enological performance and bioacidification capacity was used, suggesting that the limited acidification cannot readily be attributed to strain selection, but rather to the specific composition of the grape juice. Among the factors that may have contributed, the relatively low, near-borderline level of yeast assimilable nitrogen (YAN) in the grape juice cannot be excluded. Lactic acid production by *L.* *thermotolerans* has been shown to be influenced by nitrogen availability, with amino acid limitation linked experimentally to changes in lactate formation pathways in synthetic grape juice fermentations (Battjes et al., 2023). Nevertheless, the underlying causes of the limited lactic acid production observed here remain unclear and warrant further investigation. MP + SC and PK + SC wines had the lowest lactic acid levels (Table 1). SC wine had the highest citric acid concentration, followed by SP + SC, while SC × SPx and LT + SC wines had the lowest. Enhanced citric acid production during mixed fermentations has been reported previously (Balmaseda et al., 2018; Izquierdo Cañas et al., 2014). TD + SC and SC × SPx wines showed lower volatile acidity compared to some other treatments (Table 1), consistent with *T. delbrueckii*'s known trait of producing lower acetic acid levels (Bely et al., 2008; Cheng et al., 2025), but did not differ significantly from SC wine. pH trends followed acidity levels, with SP + SC wine having the highest pH, while other wines showed lower pH values than the control SC wine. The highest increase in glycerol was observed in SC × SPx wine, consistent with previous findings on *S. paradoxus* (Constantini et al., 2021). SC × SPx was followed by TD + SC wine, while the other wines had lower levels than SC wine.

**Table 1:** Standard physico-chemical parameters of Malvazija istarska white wines produced by fermentation with different yeasts.

Physico-chemical parameters	Wine						
	SC	SC × SPx	TD + SC	MP + SC	PK + SC	LT + SC	SP + SC
Ethanol (vol%)	13.07	12.99	12.97	13.01	13.14	13.01	13.12
Total dry extract without reducing sugars (g/L)	19.8 ^a^	20.3 ^a⁎^	19.6 ^a⁎^	19.3 ^a⁎^	19.4 ^a⁎^	19.3 ^a⁎^	18.2 ^b⁎^
Total acidity (g/L)	5.9 ^a^	5.7 ^a⁎^	5.9 ^a⁎^	5.7 ^a⁎^	5.7 ^a⁎^	5.7 ^a⁎^	4.7 ^b⁎^
Volatile acidity (g/L)	0.32 ^bc^	0.26 ^c^	0.28 ^c^	0.38 ^a^	0.37 ^ab^	0.38 ^ab^	0.38 ^ab^
Citric acid (g/L)	0.40 ^a^	0.34 ^d⁎^	0.35 ^c⁎^	0.35 ^c⁎^	0.35 ^c⁎^	0.34 ^d⁎^	0.36 ^b⁎^
Tartaric acid (g/L)	2.56 ^b^	2.57 ^ab^	2.52 ^b^	2.55 ^b^	2.64 ^a^	2.55 ^b^	2.50 ^b^
Malic acid (g/L)	2.22 ^a^	1.89 ^c⁎^	1.89 ^c⁎^	2.19 ^a⁎^	2.09 ^b⁎^	2.18 ^a^	1.10 ^d⁎^
Lactic acid (g/L)	0.08 ^b^	0.08 ^b^	0.09 ^a⁎^	0.07 ^c⁎^	0.07 ^c⁎^	0.09 ^a⁎^	0.08 ^b^
pH	3.24 ^b^	3.25 ^b⁎^	3.23 ^b⁎^	3.25 ^b⁎^	3.24 ^b⁎^	3.25 ^b⁎^	3.33 ^a⁎^
Glycerol (g/L)	6.18 ^c^	6.98 ^a⁎^	6.47 ^b⁎^	5.48 ^e⁎^	5.51 ^e⁎^	5.64 ^d⁎^	5.48 ^e⁎^

Abbreviations: SC – *Saccharomyces cerevisiae* (control, monoculture); SC × SPx – *Saccharomyces cerevisiae*×*Saccharomyces paradoxus* hybrid (monoculture); TD + SC – *Torulaspora delbrueckii + S. cerevisiae*; MP + SC – *Metschnikowia pulcherrima + S. cerevisiae*; PK + SC – *Pichia kluyveri + S. cerevisiae*; LT + SC – *Lachancea thermotolerans + S. cerevisiae*; SP + SC – *Schizosaccharomyces pombe + S. cerevisiae* (TD + SC, MP + SC, PK + SC, LT + SC, and SP + SC sequential fermentations were finished by *S. cerevisiae* (SC) inoculated at 2 vol% ethanol). Different superscript lowercase letters in a row represent statistically significant differences among wines produced using different yeasts determined by one-way ANOVA and least significant difference test (LSD) at *p* < 0.05. Asterisks represent statistically significant differences between *SC* and each other wine determined by Student's *t*-test at *p* < 0.05.

### 3.2 Volatile aroma compounds

Volatile compound concentrations determined by GC/FID, GC/MS, and GC × GC/TOF-MS are shown in Table 2. A combination of these three GC techniques, particularly GC × GC/TOF-MS, yielded one of the most comprehensive aroma profiles of Malvazija istarska wine reported to date. In total, 399 volatiles were identified or tentatively identified, including five hydrocarbons, 54 terpenes, 15 C_13_-norisoprenoids, two thiols, 11 aldehydes, 12 ketones, 44 alcohols, 24 acids, 117 esters, 59 benzenoids, nine volatile phenols, 28 furanoids and lactones, 18 sulfur-containing compounds and one other compound. Significant differences were found between wines for most compounds.

**Table 2:** Concentrations (μg/L if not otherwise indicated) of volatile aroma compounds found in Malvazija istarska white wines produced by fermentation with different yeasts determined by gas chromatography with flame-ionization detection (GC-FID)^¤^, one-dimensional gas chromatography–mass spectrometry (GC/MS)^‡^ and two-dimensional gas chromatography–mass spectrometry with time-of flight mass spectrometry (GC × GC/TOF-MS) sorted by compound class and descending Fisher *F*-ratio.

Code	Volatile compounds	ID	LRI_exp_	LRI_lit_	*F*-ratio	Wine						
						SC	SC × SPx	TD + SC	MP + SC	PK + SC	LT + SC	SP + SC
	*Hydrocarbons*											
HY1	Azulene	MS, LRI	1754	1746	6.718	2.03 ^bc^	2.26 ^a⁎^	1.90 ^c^	2.13 ^ab⁎^	2.12 ^ab^	1.87 ^c^	2.16 ^ab^
HY2	*trans, cis*-2,4-Dodecadiene	MS, LRI	1404	1402	3.965	0.450 ^a^	0.236 ^b⁎^	0.311 ^ab^	0.308 ^ab^	0.436 ^a^	0.192 ^b⁎^	0.427 ^a^
HY3	1,3,5,5-Tetramethyl-1,3-cyclohexadiene ^‡^	MS	1405	1370	3.665	0.429 ^ab^	0.328 ^bc⁎^	0.484 ^a^	0.426 ^ab^	0.455 ^a^	0.387 ^abc^	0.274 ^c⁎^
HY4	*trans*-1-Ethyl-2-methyl-cyclohexane	MS	1350	–	2.442	0.035 ^c^	0.398 ^a⁎^	0.285 ^ab⁎^	0.297 ^ab⁎^	0.145 ^bc^	0.259 ^abc^	0.228 ^abc^
HY5	3-Methylene-4-vinylcyclohex-1-ene	MS	1672	–	2.416	0.076 ^bc^	0.093 ^ab⁎^	0.082 ^abc^	0.103 ^a^	0.089 ^abc^	0.096 ^ab⁎^	0.071 ^c^
	*Terpenoids*											
TE1	β-Bisabolene	MS, LRI	1698	1699	33.887	0.009 ^b^	0.055 ^b⁎^	0.017 ^b^	0.006 ^b^	0.053 ^b^	0.407 ^a⁎^	0.033 ^b^
TE2	Geranyl acetate	MS, LRI	1760	1759	21.847	0.89 ^c^	0.43 ^d⁎^	1.71 ^a⁎^	0.66 ^cd^	0.60 ^cd⁎^	0.89 ^c^	1.38 ^b^
TE3	Terpenoid n.i. I	MS	1782	–	20.793	0.563 ^bc^	0.680 ^a⁎^	0.206 ^f⁎^	0.632 ^ab^	0.503 ^cd^	0.429 ^de⁎^	0.383 ^e⁎^
TE4	Citronellol	S, MS, LRI	1766	1760	19.804	1.43 ^a^	0.97 ^b⁎^	0.77 ^c⁎^	0.83 ^bc⁎^	0.86 ^bc⁎^	0.76 ^c⁎^	0.78 ^c⁎^
TE5	β-Pinene ^‡^	MS, LRI	1146	1145	18.387	8.16 ^ab^	5.51 ^c⁎^	9.05 ^a^	7.16 ^b^	5.84 ^c^	5.40 ^c⁎^	4.11 ^d⁎^
TE6	Citronellyl acetate	MS, LRI	1666	1659	12.323	0.806 ^a^	0.507 ^bc⁎^	0.799 ^a^	0.364 ^c⁎^	0.409 ^bc⁎^	0.412 ^bc⁎^	0.531 ^b⁎^
TE7	Terpenoid n.i. II	MS	1716	–	11.175	0.191 ^b^	0.176 ^b^	0.262 ^a^	0.165 ^b^	0.149 ^bc⁎^	0.101 ^c⁎^	0.267 ^a^
TE8	*trans*-2-Pinanol	MS, LRI	1520	1522	10.623	3.66 ^a^	1.59 ^c⁎^	2.22 ^b⁎^	2.77 ^b⁎^	2.67 ^b⁎^	2.69 ^b⁎^	2.57 ^b⁎^
TE9	Epoxyterpinolene	MS, LRI	1492	1486	9.730	1.51 ^a^	0.70 ^c⁎^	0.95 ^b⁎^	1.12 ^b⁎^	1.11 ^b⁎^	1.12 ^b⁎^	1.04 ^b⁎^
TE10	3-Carene	MS. LRI	1146	1143	9.401	0.38 ^c^	0.49 ^c^	1.05 ^a⁎^	0.44 ^c^	0.46 ^c^	0.60 ^bc⁎^	0.73 ^b^
TE11	α-Bisabolene	MS, LRI	1735	1740	7.398	0.240 ^bc^	0.604 ^a⁎^	0.270 ^bc^	0.314 ^b^	0.159 ^c^	0.331 ^b⁎^	0.331 ^b^
TE12	Geraniol	S, MS, LRI	1847	1847	5.382	1.10 ^b^	0.78 ^c^	1.02 ^bc^	1.13 ^b^	1.20 ^b^	0.97 ^bc^	1.53 ^a^
TE13	Geranial	MS, LRI	1741	1743	4.045	0.139 ^bc^	0.061 ^c^	0.265 ^ab^	0.267 ^a^	0.193 ^ab^	0.212 ^ab^	0.306 ^a⁎^
TE14	Nerol oxide	MS, LRI	1477	1473	2.902	4.07 ^abc^	4.76 ^a⁎^	4.06 ^abc^	4.91 ^a^	4.41 ^ab^	3.37 ^c^	3.57 ^bc^
TE15	α-Calacorene	MS, LRI	1926	1928	2.807	0.397 ^ab^	0.422 ^a^	0.352 ^b^	0.410 ^a^	0.348 ^b⁎^	0.355 ^b^	0.375 ^ab^
TE16	Linalool ^‡^	SC, MS, LRI	1542	1542	2.269	29.82 ^abc^	29.02 ^bc^	33.68 ^a^	32.85 ^ab^	28.62 ^c^	32.21 ^abc^	30.43 ^abc^
TE17	γ-Terpinene	MS, LRI	1245	1239	2.133	2.93 ^b^	3.90 ^a⁎^	2.68 ^b^	3.17 ^ab^	3.19 ^ab^	2.99 ^b^	3.15 ^ab^
TE18	Limonene	S, MS, LRI	1188	1196	2.094	3.99 ^b^	5.76 ^ab^	5.90 ^ab^	5.73 ^ab^	6.97 ^a⁎^	6.57 ^a⁎^	5.95 ^ab⁎^
TE19	Menthol	MS, LRI	1641	1641	2.064	0.160 ^ab^	0.146 ^b^	0.151 ^b^	0.163 ^ab^	0.142 ^b⁎^	0.136 ^b⁎^	0.201 ^a^
TE20	β-Phellandrene	MS, LRI	1203	1218	2.030	2.92 ^b^	2.79 ^b^	2.87 ^b^	3.46 ^a^	3.18 ^ab^	3.03 ^ab^	2.93 ^b^
TE21	Nerol	S, MS, LRI	1804	1801	1.893	0.983 ^a^	0.784 ^b^	0.824 ^ab^	0.958 ^ab^	0.912 ^ab^	0.836 ^ab^	1.016 ^a^
TE22	Isomenthone	MS, LRI	1487	1489	1.819	0.319 ^ab^	0.350 ^ab^	0.225 ^b^	0.400 ^a^	0.298 ^ab^	0.390 ^ab^	0.450 ^a⁎^
TE23	Carvone	MS, LRI	1741	1742	1.679	0.055 ^b^	0.110 ^ab^	0.094 ^ab^	0.082 ^ab^	0.094 ^ab^	0.141 ^a⁎^	0.053 ^b^
TE24	β-Myrcene	S, MS, LRI	1160	1159	1.639	11.44 ^b^	18.15 ^a⁎^	15.93 ^ab^	17.46 ^a⁎^	14.45 ^ab^	17.36 ^a⁎^	15.11 ^ab^
TE25	γ-Isogeraniol	MS, LRI	1814	1820	1.581	0.103 ^ab^	0.118 ^ab^	0.119 ^ab^	0.169 ^a^	0.174 ^a^	0.074 ^b^	0.131 ^ab^
TE26	α-Isomethyl ionone ^‡^	MS, LRI	1835	1848	1.558	1.43 ^ab^	1.14 ^b^	1.24 ^b^	1.32 ^ab^	1.10 ^b^	1.81 ^ab^	2.57 ^a^
TE27	4-Terpineol	S, MS, LRI	1604	1604	1.524	0.749 ^a^	0.670 ^ab^	0.678 ^ab^	0.745 ^a^	0.654 ^ab^	0.499 ^b^	0.730 ^a^
TE28	*trans*-β-Ocimene	S, MS, LRI	1253	1258	1.455	9.80 ^b^	10.95 ^ab^	10.28 ^ab^	11.98 ^ab^	10.84 ^ab^	13.20 ^a^	10.14 ^b^
TE29	Terpenoid n.i. III	MS	1203	–	1.452	2.58 ^b^	3.84 ^ab⁎^	3.36 ^ab^	3.57 ^ab⁎^	4.62 ^a^	3.85 ^ab⁎^	4.17 ^ab⁎^
TE30	Geranyl acetone	MS, LRI	1860	1856	1.442	3.90 ^ab^	4.05 ^ab^	4.88 ^ab^	4.15 ^ab^	2.98 ^b⁎^	2.93 ^b⁎^	5.63 ^a^
TE31	Cadalene	MS, LRI	2227	2226	1.401	0.217 ^ab^	0.225 ^ab^	0.203 ^ab^	0.236 ^a^	0.191 ^b^	0.216 ^ab^	0.213 ^ab^
TE32	Terpenoid n.i. IV	MS	1456	–	1.398	38.07 ^ab^	47.23 ^a^	38.80 ^ab^	42.93 ^ab^	39.11 ^ab^	45.27 ^ab^	35.46 ^b^
TE33	Isomyocorene	MS	1203	–	1.394	0.463 ^ab^	0.519 ^ab^	0.364 ^b^	0.494 ^ab^	0.497 ^ab^	0.681 ^a^	0.514 ^ab^
TE34	*trans*-Linalool furan oxide	S, MS, LRI	1445	1450	1.390	0.92 ^ab^	0.91 ^ab^	1.00 ^ab^	1.09 ^a^	0.84 ^ab^	1.09 ^a^	0.63 ^b^
TE35	α-Phellandrene	MS, LRI	1160	1160	1.352	0.257 ^ab^	0.171 ^b^	0.410 ^a^	0.148 ^b^	0.290 ^ab^	0.285 ^ab^	0.182 ^ab^
TE36	*cis*-Calamenene	MS, LRI	1841	1840	1.217	0.322	0.314	0.309	0.322	0.288 ^⁎^	0.284	0.320
TE37	Ho-trienol	MS, LRI	1610	1612	1.199	12.28 ^ab^	12.59 ^ab^	11.21 ^b^	13.65 ^a^	12.20 ^ab^	12.40 ^ab^	12.93 ^ab^
TE38	α-Terpineol	MS, LRI	1704	1701	1.192	11.94 ^ab^	11.85 ^ab^	11.14 ^b^	13.43 ^a^	12.15 ^ab^	12.26 ^ab^	11.96 ^ab^
TE39	α-Ocimene	MS, LRI	1235	1245	1.165	11.17	13.24	13.88	14.54	12.23	14.41	12.08
TE40	Geranyl ethyl ether	MS, LRI	1514	1506	1.104	7.75	9.40	8.02	9.30	8.23	9.61	7.87
TE41	Neryl ethyl ether	MS, LRI	1482	1477	1.075	1.43	1.72	1.43	1.64	1.52	1.65	1.44
TE42	Neomenthol	MS, LRI	1587	1586	0.981	0.266	0.261	0.241	0.276	0.253	0.262	0.281
TE43	*cis*-Alloocimene	MS, LRI	1382	1369	0.971	1.18	1.22	1.20	1.39	1.29	1.38	1.21
TE44	Cosmene	MS, LRI	1456	1460	0.937	3.35	3.87	3.46	4.08	3.52	4.01	3.54
TE45	Longifolene	MS, LRI	1575	1575	0.932	0.193	0.168	0.196	0.201	0.131	0.168	0.194
TE46	*p*-Menth-1-en-9-al	MS, LRI	1622	1629	0.876	0.95	1.12	1.08	1.22	1.05	1.11	1.03
TE47	α-Terpinolene	S, MS, LRI	1287	1274	0.875	7.12	9.54	7.93	8.37	7.71	7.16	7.79
TE48	*trans*-Alloocimene	MS, LRI	1403	1400	0.776	1.36	1.45	1.52	1.61	1.45	1.63 ^⁎^	1.44
TE49	Dihydromyrcenol	MS, LRI	1468	1455	0.586	0.74	0.58	2.26	0.46	1.22	1.21	1.31
TE50	*cis*-Linalool furan oxide	MS, LRI	1436	1438	0.531	0.78	0.80	3.77	0.24	1.52	2.51	2.09
TE51	Farnesene I	MS, LRI	1672	1685	0.405	1.48	1.57	1.43	1.67	1.51	1.60	1.63
TE52	Linalool ethyl ether	MS, LRI	1329	1331	0.303	17.67	19.09	16.99	18.24	18.48	18.42	17.41
TE53	α-Curcumene	MS, LRI	1785	1782	0.131	0.223	0.210	0.220	0.226	0.208	0.223	0.209
TE54	Farnesene II	MS, LRI	1754	1757	0.119	0.306	0.313	0.322	0.322	0.325	0.347	0.335
	*Norisoprenoids*											
NO1	Norisoprenoid n.i. ^‡^	MS	2212	–	7.998	0.623 ^b^	0.627 ^b^	0.975 ^a⁎^	0.534 ^b^	0.427 ^bc^	0.500 ^b^	0.215 ^c⁎^
NO2	β-Cyclocitral	S, MS, LRI	1629	1630	5.324	0.264 ^a^	0.175 ^b⁎^	0.249 ^a^	0.298 ^a^	0.264 ^a^	0.254 ^a^	0.270 ^a^
NO3	Vitispirane I ^‡^	MS, LRI	1521	1524	3.906	0.94 ^d^	1.31 ^abc⁎^	1.10 ^bcd^	1.15 ^bcd^	1.05 ^cd^	1.53 ^a⁎^	1.42 ^ab^
NO4	Safranal	MS, LRI	1654	1648	2.228	0.240 ^b^	0.229 ^b^	0.230 ^b^	0.294 ^a^	0.263 ^ab^	0.264 ^ab^	0.273 ^ab^
NO5	*trans*-β-Damascenone	S, MS, LRI	1829	1829	1.757	29.98 ^ab^	33.48 ^ab^	29.21 ^b^	35.48 ^ab^	30.89 ^ab^	30.36 ^ab^	36.10 ^a^
NO6	1,2-Dihydro-1,4,6-trimethylnaphthalene	MS, LRI	2097	2071	1.739	0.072 ^ab^	0.101 ^a^	0.078 ^ab^	0.085 ^ab^	0.078 ^ab^	0.097 ^a^	0.065 ^b^
NO7	α-Ionene	MS, LRI	1560	1567	1.618	0.411 ^ab^	0.592 ^a⁎^	0.440 ^ab^	0.519 ^ab^	0.472 ^ab^	0.568 ^a^	0.357 ^b^
NO8	*cis*-β-Damascenone	MS, LRI	1772	1774	1.509	2.70 ^ab^	3.05 ^ab^	2.61 ^b^	3.29 ^a^	2.80 ^ab^	2.89 ^ab^	3.17 ^ab^
NO9	Vitispirane II	MS, LRI	1537	1543	1.454	3.18 ^ab^	3.98 ^a^	3.37 ^ab^	3.67 ^ab^	3.32 ^ab^	3.80 ^ab^	2.97 ^b^
NO10	Theaspirane isomer II	MS, LRI	1553	1550	1.449	1.04 ^ab^	1.24 ^a^	1.05 ^ab^	1.07 ^ab^	1.03 ^ab^	0.99 ^b^	1.19 ^ab^
NO11	Theaspirane isomer I	MS, LRI	1537	1523	1.409	1.96 ^b^	2.46 ^a⁎^	2.03 ^ab^	2.16 ^ab^	2.00 ^ab^	2.28 ^ab^	2.05 ^ab^
NO12	1,2-Dihydro-1,5,8-trimethylnaphthalene	MS, LRI	1760	1757	1.276	1.84	2.31	2.00	2.26	1.98	2.29	1.78
NO13	*trans*-1-(2,3,6-Trimethylphenyl)buta-1,3-diene (TPB)	MS, LRI	1835	1832	1.221	0.566	0.760	0.656	0.750	0.592	0.730	0.549
NO14	1,1,6-Trimethyl-1,2-dihydronaphthalene (TDN)	S, MS, LRI	1722	1722	1.207	0.208	0.265	0.234	0.277	0.219	0.264	0.203
NO15	β-Ionone ^‡^	MS, LRI	1916	1915	0.852	0.781	0.685	0.678	0.781	0.584	0.888	0.788
	*Thiols*											
TH1	3-Mercaptohexyl acetate (3MHA) (ng/L) ^‡^	SC, MS	–	–	37.448	30.33 ^de^	49.27 ^cd^	151.7 ^a^	67.41 ^bc^	24.50 ^e^	79.98 ^b^	66.18 ^bc^
TH2	3-Mercaptohexan-1-ol (3MH) (ng/L) ^‡^	SC, MS	–	–	7.561	906.3 ^b^	1371.2 ^a^	1363.1 ^a^	1216.3 ^a^	1315.7 ^a^	828.4 ^b^	1409.6 ^a^
	*Aldehydes*											
AD1	Acetaldehyde (mg/L) ^¤^	SC	<1100	714	26.495	54.77 ^a^	31.10 ^d⁎^	23.93 ^e⁎^	38.50 ^bc⁎^	35.63 ^cd⁎^	38.84 ^bc⁎^	42.22 ^b⁎^
AD2	2-Nonenal	MS, LRI	1542	1540	13.326	0.050 ^cd^	0.024 ^d⁎^	0.063 ^cd^	0.027 ^cd^	0.074 ^bc⁎^	0.123 ^b⁎^	0.188 ^a⁎^
AD3	2-(Acetoxy)-propanal	MS	1829	–	7.567	1.43 ^c^	2.59 ^a⁎^	1.16 ^c^	1.64 ^bc^	1.49 ^c^	1.25 ^c^	2.10 ^ab^
AD4	Tetradecanal	MS, LRI	1926	1923	2.754	0.148 ^b^	0.179 ^b^	0.170 ^b^	0.272 ^a⁎^	0.147 ^b^	0.190 ^b^	0.198 ^ab^
AD5	Nonanal	MS, LRI	1403	1403	2.260	24.12 ^b^	29.17 ^ab^	19.81 ^b^	26.95 ^ab^	28.94 ^ab^	30.09 ^ab^	36.22 ^a^
AD6	Octanal	MS, LRI	1293	1281	1.962	1.54 ^b^	2.14 ^ab^	2.02 ^b^	2.11 ^ab^	2.26 ^ab^	1.80 ^b^	3.19 ^ab⁎^
AD7	2,6,6-Trimethyl-1-cyclohexene-1-acrolein	MS	1947	–	1.603	0.252 ^ab^	0.267 ^ab^	0.239 ^b^	0.292 ^a^	0.256 ^ab^	0.262 ^ab^	0.230 ^b^
AD8	Undecanal	S, MS, LRI	1610	1610	1.244	0.443 ^b^	0.662 ^a^	0.541 ^ab^	0.431 ^b^	0.506 ^ab^	0.532 ^ab^	0.573 ^ab^
AD9	Decanal	S, MS, LRI	1503	1504	1.135	11.16 ^ab^	17.99 ^a^	13.94 ^ab^	7.89 ^b^	13.85 ^ab^	13.82 ^ab^	13.09 ^ab^
AD10	Dodecanal	MS, LRI	1716	1713	0.907	0.548	0.840	0.754	0.789	0.615	0.750	0.857
AD11	Cyclomyral	MS	1722	–	0.881	0.96	0.90	0.86	1.02	0.95	0.89	0.88
	*Ketones*											
KE1	1,2-Dihydroxycyclobutene-3,4-dione	MS	1672	–	97.587	0.71^d^	0.38 ^e⁎^	1.86 ^a⁎^	1.55 ^b⁎^	1.57 ^b⁎^	1.44 ^b⁎^	1.14 ^c⁎^
KE2	2-Undecanone	MS, LRI	1598	1598	62.609	4.13 ^a^	1.89 ^b⁎^	1.22 ^d⁎^	1.52 ^bcd⁎^	1.56 ^bcd⁎^	1.42 ^cd⁎^	1.67 ^bcd⁎^
KE3	2-Nonanone	S, MS, LRI	1393	1392	47.956	59.03 ^a^	26.89 ^b⁎^	14.52 ^c⁎^	17.61 ^c⁎^	21.17 ^bc⁎^	17.24 ^c⁎^	18.03 ^c⁎^
KE4	3-(Acetoxy)-4-methyl-2-pentanone	MS	1466	–	20.096	0.266 ^a^	0.290 ^a^	0.104 ^c⁎^	0.196 ^b^	0.218 ^b^	0.204 ^b⁎^	0.136 ^c⁎^
KE5	2-Ethyl-1,6-dioxaspiro[4,4]nonane	MS	1357	–	3.952	0.535 ^bc^	0.707 ^a⁎^	0.448 ^c^	0.473 ^c^	0.490 ^c^	0.694 ^ab⁎^	0.516 ^c^
KE6	Acetoin	S, MS, LRI	1287	1285	3.471	1.24 ^bc^	1.60 ^a^	0.92 ^c^	1.36 ^ab^	1.27 ^ab^	1.18 ^bc^	1.43 ^ab^
KE7	2,3-Dihydro-3,3,4,5-tetramethyl-1H-inden-1-one	MS	2154	–	1.943	0.111 ^ab^	0.122 ^ab^	0.104 ^b^	0.133 ^a^	0.114 ^ab^	0.115 ^ab^	0.131 ^a^
KE8	6-Methyl-5-hepten-2-one	MS, LRI	1345	1343	1.203	0.612 ^ab^	0.566 ^ab^	0.568 ^ab^	0.632 ^ab^	0.434 ^b⁎^	0.472 ^ab^	0.671 ^a^
KE9	2-Decanone	MS, LRI	1498	1503	1.025	1.44 ^ab^	1.40 ^ab^	1.34 ^ab^	1.43 ^ab^	1.33 ^b^	1.41 ^ab^	1.56 ^a^
KE10	Limona ketone	MS, LRI	1559	1568	0.925	0.592	0.547	0.574	0.773	0.770	0.847 ^⁎^	0.644
KE11	*p*-*tert*-Butylcyclohexanone	MS, LRI	1641	1645	0.733	0.430	0.387	0.364	0.430	0.374	0.387	0.377
KE12	2-Dodecanone	MS, LRI	1710	1709	0.660	0.751	0.717	0.794	0.768	0.706	0.705	0.895
	*Alcohols*											
AL1	3-Methylpentanol	S, MS, LRI	1329	1322	273.814	187.5 ^b^	215.3 ^a⁎^	60.7 ^de⁎^	82.6 ^c⁎^	69.8 ^d⁎^	54.4 ^e⁎^	61.0 ^de⁎^
AL2	2-Phenylethanol (mg/L) ^‡^	SC, MS, LRI	1891	1893	87.406	29.75 ^b^	46.01 ^a⁎^	24.58 ^c⁎^	27.89 ^bc^	16.38 ^d⁎^	18.51 ^d⁎^	16.72 ^d⁎^
AL3	1-Propanol (mg/L) ^¤^	SC	–	1035	64.967	26.38 ^b^	19.14 ^c⁎^	29.47 ^a⁎^	13.19 ^e⁎^	15.80 ^d⁎^	18.65 ^c⁎^	18.23 ^c⁎^
AL4	2-Undecanol	MS, LRI	1722	1723	50.712	3.16 ^a^	1.19 ^c⁎^	1.85 ^b⁎^	1.01 ^c⁎^	1.08 ^c⁎^	0.92 ^c⁎^	1.06 ^c⁎^
AL5	Butanediol derivative I	MS, LRI	1610	1600	37.988	2.66 ^c^	6.30 ^a⁎^	4.67 ^b⁎^	2.92 ^c^	3.35 ^c⁎^	5.75 ^a⁎^	4.06 ^b⁎^
AL6	2,7-Dimethyl-4,5-octanediol	MS	1741	–	36.621	0.386 ^b^	0.708 ^a⁎^	0.176 ^d⁎^	0.367 ^b^	0.261 ^cd⁎^	0.233 ^cd⁎^	0.269 ^c⁎^
AL7	1-Heptanol	S, MS, LRI	1456	1457	30.248	5.14 ^bc^	5.64 ^b⁎^	4.24 ^c^	8.41 ^a⁎^	7.96 ^a⁎^	8.29 ^a⁎^	8.21 ^a⁎^
AL8	Isobutanol (mg/L) ^¤^	SC, MS, LRI	1090	1098	29.783	19.83 ^e^	24.34 ^d^	31.10 ^bc⁎^	30.00 ^bc⁎^	35.08 ^a⁎^	32.73 ^ab⁎^	28.90 ^c⁎^
AL9	1-Nonanol	S, MS, LRI	1660	1661	29.665	2.82 ^d^	0.65 ^e⁎^	2.30 ^d^	4.07 ^ab⁎^	3.60 ^bc^	3.16 ^cd^	4.40 ^a⁎^
AL10	*cis*-6-Nonen-1-ol	MS, LRI	1716	1714	27.522	0.71 ^c^	0.78 ^c⁎^	0.44 ^d⁎^	1.15 ^ab⁎^	1.08 ^b⁎^	1.30 ^a⁎^	1.34 ^a⁎^
AL11	2-Nonanol	S, MS, LRI	1520	1518	25.227	26.53 ^a^	14.70 ^b⁎^	12.97 ^bc⁎^	13.38 ^bc⁎^	12.60 ^bc⁎^	11.42 ^c⁎^	12.49 ^bc⁎^
AL12	3-Methyl-3-buten-1-ol	MS, LRI	1245	1244	23.647	0.93 ^a^	1.00 ^a^	0.55 ^c⁎^	0.62 ^bc⁎^	0.66 ^bc⁎^	0.57 ^c⁎^	0.69 ^b⁎^
AL13	Butanediol derivative II	MS	1654	–	22.703	4.83 ^c^	9.18 ^b⁎^	7.98 ^b⁎^	8.19 ^b⁎^	9.25 ^b⁎^	11.13 ^a⁎^	12.30 ^a⁎^
AL14	Isoamyl alcohol (mg/L) ^¤^	SC, MS, LRI	1229	1229	21.905	175.0 ^bc^	222.0 ^a⁎^	161.2 ^cd⁎^	179.0 ^b⁎^	188.1 ^b^	157.9 ^de⁎^	141.3 ^e⁎^
AL15	1-Octanol	MS, LRI	1553	1558	18.579	36.98 ^bc^	41.14 ^b⁎^	18.97 ^d⁎^	49.71 ^a⁎^	38.35 ^bc^	32.96 ^c⁎^	34.88 ^bc^
AL16	2,3-Butanediol isomer I	S, MS, LRI	1537	1542	16.426	101.6 ^ab^	109.5 ^a^	72.3 ^e⁎^	81.5 ^de⁎^	84.6 ^cd⁎^	83.0 ^cd^*	92.5 ^bc⁎^
AL17	*cis*-3-Octen-1-ol	MS	1575	–	11.369	0.019 ^c^	0.558 ^a⁎^	0.052 ^c^	0.267 ^bc⁎^	0.426 ^ab⁎^	0.421 ^ab⁎^	0.363 ^b⁎^
AL18	2,3-Butanediol isomer II	S, MS, LRI	1575	1576	8.421	24.06 ^bc^	27.05 ^a^	22.24 ^bcd⁎^	19.37 ^e^	20.45 ^de⁎^	21.55 ^cde⁎^	24.66 ^ab^
AL19	1-Hexanol (mg/L) ^‡^	SC, MS, LRI	1356	1357	6.936	1.38 ^ab^	1.29 ^bc^	1.11 ^c⁎^	1.53 ^a^	1.20 ^c⁎^	1.48 ^a^	1.23 ^bc^
AL20	Methanol (mg/L) ^¤^	SC	<1000	911	6.551	51.03 ^a^	41.14 ^b^	39.48 ^bc⁎^	33.47 ^c⁎^	43.61 ^b⁎^	40.46 ^b⁎^	41.86 ^b⁎^
AL21	*trans*-2-*tert*-Butylcyclohexan-1-ol	MS	1610	–	6.463	0.758 ^ab^	0.878 ^a^	0.585 ^cd^	0.717 ^bc^	0.610 ^bcd⁎^	0.494 ^d⁎^	0.623 ^bcd^
AL22	2-Ethyl-1-hexanol	MS, LRI	1482	1490	6.020	11.15 ^bc^	10.89 ^bc^	9.80 ^c^	12.10 ^ab^	9.48 ^c^	9.29 ^c^	13.64 ^ab⁎^
AL23	4-Allyl-1,6-heptadiene-4-ol	MS	1791	–	4.887	0.381 ^bc^	0.353 ^c^	0.342 ^c^	0.437 ^ab^	0.410 ^bc^	0.436 ^ab⁎^	0.494 ^a⁎^
AL24	3-Nonanol	MS, LRI	1492	1493	4.466	0.408 ^a^	0.361 ^ab⁎^	0.334 ^bc^	0.365 ^ab^	0.334 ^bc^	0.279 ^c⁎^	0.412 ^a^
AL25	*cis*-3-Hexen-1-ol ^‡^	SC, MS, LRI	1389	1389	4.269	50.36 ^b^	50.82 ^b^	41.62 ^c^	58.42 ^a^	52.68 ^ab^	48.74 ^bc^	49.53 ^b^
AL26	*trans*-2-Octen-1-ol	S, MS, LRI	1616	1618	4.176	1.52 ^c^	1.52 ^c^	1.56 ^c^	1.84 ^ab⁎^	1.62 ^bc^	1.73 ^abc⁎^	1.98 ^a⁎^
AL27	3-Ethyl-4-methylpentan-1-ol	MS	1509	–	3.859	0.97 ^bc^	0.99 ^bc^	0.88 ^c^	1.20 ^a^	1.07 ^ab⁎^	1.06 ^abc⁎^	1.20 ^a⁎^
AL28	*cis*-2-Hexen-1-ol ^‡^	SC, MS, LRI	1416	1413	3.826	13.85 ^abcd^	10.46 ^d^	11.28 ^cd^	15.59 ^ab^	14.58 ^abc^	12.84 ^bcd^	17.31 ^a^
AL29	*cis*-4-Decen-1-ol	MS, LRI	1797	1797	3.270	0.269 ^b^	0.257 ^b^	0.252 ^b^	0.462 ^a^	0.399 ^ab^	0.405 ^ab^	0.484 ^a^
AL30	3-Octanol	MS, LRI	1392	1393	3.139	1.08 ^c^	1.33 ^ab⁎^	1.13 ^bc^	1.46 ^a⁎^	1.34 ^ab^	1.39 ^⁎^	1.32 ^ab⁎^
AL31	1-Octen-3-ol	S, MS, LRI	1450	1452	3.044	19.07 ^abc^	19.76 ^ab^	16.31 ^c^	21.85 ^a^	19.88 ^ab^	18.00 ^bc^	20.15 ^ab^
AL32	2-Heptanol	S, MS, LRI	1319	1312	3.019	1.58 ^bc^	0.46 ^c⁎^	1.10 ^bc^	3.52 ^ab^	4.67 ^a^	3.07 ^ab⁎^	2.69 ^abc^
AL33	*trans*-3-Hexen-1-ol ^‡^	SC, MS, LRI	1366	1361	2.818	77.48 ^ab^	75.25 ^abc^	64.49 ^c⁎^	84.36 ^a^	74.01 ^abc^	74.47 ^abc^	71.29 ^bc^
AL34	2-Methyl-5-nonanol	MS	1575	–	2.663	0.418 ^ab^	0.391 ^ab^	0.383 ^abc^	0.389 ^abc^	0.357 ^bc⁎^	0.326 ^c⁎^	0.425 ^a^
AL35	4-*tert*-Butylcyclohexanol	MS	1752	–	2.491	0.196 ^abc^	0.091 ^c^	0.102 ^bc⁎^	0.209 ^ab^	0.171 ^abc^	0.198 ^abc^	0.253 ^a^
AL36	*trans*-2-Pentenol	MS, LRI	1319	1316	2.448	0.592 ^ab^	0.635 ^a^	0.532 ^b^	0.642 ^a^	0.663 ^a⁎^	0.584 ^ab^	0.649 ^a⁎^
AL37	1-Undecanol	MS, LRI	1866	1871	1.992	0.244 ^a^	0.212 ^ab^	0.242 ^a^	0.273 ^a^	0.146 ^b⁎^	0.214 ^ab^	0.239 ^a^
AL38	1-Hexadecanol	MS, LRI	2372	2364	1.390	0.189 ^ab^	0.164 ^b^	0.169 ^ab^	0.262 ^ab^	0.218 ^ab^	0.163 ^b^	0.317 ^a^
AL39	6-Methyl-5-hepten-2-ol	S, MS, LRI	1461	1460	1.306	0.143 ^b^	0.155 ^ab^	0.166 ^ab^	0.206 ^a^	0.173 ^ab^	0.182 ^ab⁎^	0.144 ^b^
AL40	1-Tridecanol	MS, LRI	2068	2063	1.302	0.074	0.042	0.055	0.075	0.046	0.032	0.025
AL41	3,4,7-Trimethyl-1,5-octadien-4-ol	MS	1414	–	1.100	0.118 ^ab^	0.129 ^ab^	0.047 ^b⁎^	0.156 ^ab^	0.169 ^ab^	0.138 ^ab^	0.352 ^a^
AL42	Polyol n.i.	MS, LRI	2328	2322	1.026	3.78	3.45	2.02	2.05 ^⁎^	3.13	3.66	2.62
AL43	1-Dodecanol	MS, LRI	1968	1973	0.852	1.81	1.66	2.20	2.40	1.74	2.29	4.64
AL44	3-Dodecanol	MS, LRI	1797	1792	0.656	0.303	0.303	0.417	0.179	0.329	0.410	0.714
	*Acids*											
AC1	2-Methylbutyric acid	MS, LRI	1679	1674	113.961	83.6 ^b^	138.6 ^a⁎^	71.8 ^c^	69.2 ^c⁎^	68.1 ^c⁎^	56.7 ^d⁎^	47.1 ^e⁎^
AC2	Isovaleric acid	S, MS, LRI	1672	1675	105.861	198.5 ^a^	199.0 ^a^	84.2 ^e⁎^	152.2 ^b⁎^	140.3 ^b⁎^	119.8 ^c⁎^	101.4 ^d⁎^
AC3	Isobutyric acid	S, MS, LRI	1570	1570	59.443	26.32 ^d^	49.02 ^b⁎^	74.22 ^a⁎^	40.80 ^c⁎^	40.27 ^c⁎^	43.29 ^bc⁎^	24.30 ^d^
AC4	*trans*-2-Hexenoic acid	MS, LRI	1968	1967	59.016	1.54 ^a^	0.66 ^c⁎^	0.56 ^c⁎^	0.88 ^b⁎^	1.45 ^a^	0.91 ^b⁎^	0.98 ^b⁎^
AC5	Pivalic acid	MS, LRI	1581	1586	8.807	1.18 ^bc^	1.48 ^a⁎^	1.01 ^c⁎^	1.13 ^bc^	1.44 ^a⁎^	1.19 ^b^	1.14 ^bc^
AC6	Acetic acid ^‡^	MS, LRI	1445	1439	7.379	8.07 ^bc^	5.49 ^c^	5.46 ^c^	10.44 ^ab^	11.32 ^a^	11.57 ^a^	10.67 ^ab^
AC7	Octanoic acid (mg/L) ^‡^	SC, MS, LRI	2043	2042	6.585	6.85 ^a^	5.63 ^cd⁎^	4.83 ^d⁎^	6.66 ^abc^	5.95 ^bc^	6.48 ^ab^	5.98 ^bc^
AC8	*cis*-2-Octenoic acid	MS	2125	–	4.711	0.311 ^ab^	0.228 ^bc^	0.164 ^c⁎^	0.304 ^ab^	0.304 ^ab^	0.293 ^ab^	0.355 ^a^
AC9	Hexanoic acid (mg/L) ^‡^	SC, MS, LRI	1824	1828	4.146	5.86 ^ab^	4.86 ^c⁎^	4.84 ^c⁎^	5.89 ^ab^	6.31 ^a^	5.99 ^ab^	5.43 ^bc^
AC10	Undecanoic acid	MS, LRI	2340	2359	4.003	0.006 ^c^	0.062 ^ab⁎^	0.007 ^c^	0.004 ^c^	0.069 ^a⁎^	0.021 ^bc^	0.068 ^a^
AC11	3-Octenoic acid	MS	2104	–	3.881	0.352 ^a^	0.313 ^a⁎^	0.250 ^b⁎^	0.357 ^a^	0.334 ^a^	0.355 ^a^	0.348 ^a^
AC12	Isohexanoic acid	MS, LRI	1810	1809	2.962	0.528 ^a^	0.506 ^ab^	0.379 ^abc^	0.404 ^abc^	0.307 ^c⁎^	0.355 ^bc^	0.313 ^c⁎^
AC13	Nonanoic acid	S, MS, LRI	2180	2178	2.505	1.61 ^ab^	0.16 ^b^	3.58 ^a^	0.52 ^b^	0.09 ^b^	0.24 ^b^	0.88 ^b^
AC14	Propanoic acid	S, MS, LRI	1542	1540	2.501	1.03 ^a^	0.97 ^ab^	0.75 ^bc^	0.75 ^bc^	0.88 ^abc^	0.67 ^c⁎^	0.95 ^ab^
AC15	2-Ethylhexanoic acid	MS, LRI	1954	1960	2.426	3.39 ^abc^	3.25 ^bc^	2.89 ^c^	3.76 ^abc^	3.91 ^abc^	4.26 ^ab^	4.48 ^a^
AC16	2,2-Dimethylbutyric acid	MS	1419	–	2.251	1.74 ^b^	3.01 ^a⁎^	2.15 ^b^	2.57 ^ab^	1.70 ^b^	2.12 ^ab^	2.04 ^ab^
AC17	Butyric acid ^‡^	SC, MS, LRI	1617	1612	1.964	1.70 ^a^	1.20 ^b⁎^	1.38 ^ab^	1.57 ^ab^	1.75 ^a^	1.48 ^ab^	1.36 ^ab^
AC18	2-Propenoic acid	MS	1641	–	1.791	0.802 ^a^	0.709 ^ab⁎^	0.703 ^a^	0.796 ^a^	0.783 ^ab^	0.674 ^b^	0.698 ^ab^
AC19	Tetradecanoic acid	MS, LRI	2696	2693	1.674	0.676 ^ab^	0.795 ^ab^	0.545 ^b^	0.628 ^ab^	0.976 ^a⁎^	0.798 ^ab^	0.880 ^ab^
AC20	Pentanoic acid	S, MS, LRI	1747	1751	1.447	2.62	2.73	2.21	2.22	2.88	2.71	2.37
AC21	3-Methyl-2-butenoic acid	MS, LRI	1804	1802	1.400	0.427 ^ab^	0.382 ^b^	0.438 ^ab^	0.568 ^a⁎^	0.490 ^ab^	0.448 ^ab^	0.526 ^ab^
AC22	Hexadecanoic acid	MS, LRI	2904	2903	1.031	0.355 ^ab^	0.351 ^ab^	0.246 ^b^	0.351 ^ab^	0.447 ^a^	0.409 ^ab^	0.395 ^ab^
AC23	Heptanoic acid	S, MS, LRI	1954	1955	1.015	3.41	3.39	2.89	2.58	3.15	3.36	3.19
AC24	Decanoic acid (mg/L) ^‡^	SC, MS, LRI	2257	2258	0.695	2.52	2.42	2.26	2.37	2.08	2.44	2.07
	*Ethyl esters*											
EE1	Ethyl 2-methylbutyrate ^‡^	SC, MS, LRI	1049	1049	98.633	5.56 ^b^	9.31 ^a⁎^	5.25 ^b^	5.14 ^b^	3.91 ^c⁎^	3.65 ^c⁎^	2.81 ^d⁎^
EE2	Ethyl propanoate ^‡^	MS, LRI	<1000	949	47.539	30.23 ^b^	32.00 ^b^	38.02 ^a⁎^	16.55 ^c⁎^	13.87 ^cd⁎^	15.88 ^c⁎^	11.18 ^d⁎^
EE3	Ethyl 3-methylbutyrate ^‡^	SC, MS, LRI	1065	1065	43.083	13.47 ^a^	13.47 ^a^	6.19 ^d⁎^	11.35 ^b^	7.98 ^c⁎^	7.82 ^c⁎^	5.78 ^d⁎^
EE4	Ethyl isobutyrate ^‡^	MS LRI	<1000	965	41.189	26.89 ^de^	37.38 ^b⁎^	56.86 ^a⁎^	36.21 ^b⁎^	29.10 ^cd^	33.90 ^bc⁎^	22.60 ^e⁎^
EE5	Ethyl 2-butenoate ^‡^	MS, LRI	1153	1153	34.939	13.09 ^d^	10.57 ^d^	32.85 ^a⁎^	24.79 ^b⁎^	20.20 ^c⁎^	19.69 ^c⁎^	22.77 ^bc⁎^
EE6	Ethyl 4-hexenoate I ^‡^	MS, LRI	1300	1292	29.880	0.84 ^b^	0.98 ^b^	1.78 ^a⁎^	0.88 ^b^	0.54 ^c^	0.91 ^b^	0.61 ^c^
EE7	Ethyl 2-hexenoate II	MS, LRI	1361	1357	26.972	0.287 ^b^	0.176 ^c⁎^	0.131 ^d⁎^	0.153 ^cd⁎^	0.337 ^a^	0.191 ^c⁎^	0.245 ^b⁎^
EE8	Ethyl isoamyl succinate	MS, LRI	1904	1907	24.371	5.12 ^bcd^	10.32 ^a⁎^	4.78 ^cd^	6.34 ^b^	5.75 ^bc^	4.19 ^d^	3.92 ^d⁎^
EE9	Ethyl 3-hydroxydecanoate	MS, LRI	2104	2102	24.313	2.37 ^a^	1.41 ^b⁎^	1.05 ^cd⁎^	1.20 ^bcd⁎^	0.97 ^d⁎^	0.99 ^cd⁎^	1.29 ^bc⁎^
EE10	Ethyl phenyllactate	MS, LRI	2281	2273	23.071	0.67 ^d^	1.55 ^ab⁎^	0.62 ^d^	1.81 ^a⁎^	1.40 ^b⁎^	0.98 ^c⁎^	1.10 ^c⁎^
EE11	Ethyl 3-hydroxybutyrate	MS, LRI	1520	1524	21.125	1.63 ^cd^	1.33 ^d^	4.81 ^a⁎^	2.03 ^cd^	3.84 ^b⁎^	2.07 ^cd⁎^	2.31 ^c⁎^
EE12	Ethyl *trans*-2-butenoate	MS, LRI	1160	1158	16.145	12.15 ^e^	17.32 ^cd⁎^	26.64 ^a⁎^	21.09 ^b⁎^	20.28 ^bc⁎^	16.56 ^d⁎^	22.59 ^b⁎^
EE13	Ethyl butyrate ^‡^	SC, MS, LRI	1030	1030	15.862	530.4 ^c^	469.5 ^d⁎^	641.7 ^a⁎^	595.7 ^b⁎^	531.9 ^c^	532.6 ^c^	594.8 ^b⁎^
EE14	Ethyl 2-hydroxy-4-methylvalerate	MS, LRI	1543	1547	14.956	12.13 ^e^	19.83 ^ab⁎^	12.25 ^de^	21.32 ^a⁎^	17.77 ^bc⁎^	17.50 ^bc⁎^	15.02 ^cd⁎^
EE15	Ethyl 2-hexenoate I	MS, LRI	1350	1357	13.725	29.32 ^ab^	21.02 ^cd⁎^	14.57 ^e⁎^	15.26 ^de⁎^	35.50 ^a^	19.82 ^cde⁎^	25.97 ^bc^
EE16	Ethyl 3-nonenoate	MS, LRI	1592	1587	10.541	0.072 ^bc^	0.085 ^b^	0.151 ^a⁎^	0.072 ^bc^	0.120 ^a⁎^	0.083 ^b^	0.043 ^c⁎^
EE17	Ethyl butyl succinate	MS, LRI	1797	1820	10.495	0.305 ^d^	0.795 ^abc⁎^	0.669 ^bc⁎^	0.852 ^ab⁎^	0.927 ^a⁎^	0.704 ^bc⁎^	0.632 ^c⁎^
EE18	Ethyl propyl succinate	MS, LRI	1766	1767	10.223	0.263 ^b^	0.345 ^a⁎^	0.347 ^a^	0.210 ^b^	0.249 ^b^	0.223 ^b⁎^	0.214 ^b⁎^
EE19	Ethyl methyl succinate	MS, LRI	1635	1642	10.002	0.78 ^bc^	0.95 ^b⁎^	1.13 ^a⁎^	0.71 ^c^	0.76 ^c^	0.66 ^c⁎^	0.65 ^c⁎^
EE20	Ethyl 3-hexenoate	MS, LRI	1308	1292	8.210	4.48 ^bc^	5.82 ^b^	9.13 ^a⁎^	3.53 ^c⁎^	4.77 ^bc^	4.37 ^bc^	2.94 ^c⁎^
EE21	Ethyl pentanoate	MS, LRI	1136	1140	6.468	0.041 ^c^	0.073 ^bc^	0.213 ^a⁎^	0.061 ^bc^	0.066 ^bc^	0.064 ^bc^	0.133 ^b^
EE22	Ethyl 4-hydroxybutyrate	MS, LRI	1804	1796	5.891	6.12 ^a^	2.07 ^c⁎^	2.56 ^bc⁎^	3.55 ^bc^	3.82 ^b^	3.21 ^bc^	3.46 ^bc^
EE23	Ethyl *trans*-4-octenoate	MS	1520	–	5.267	0.091 ^abc^	0.077 ^cd^	0.067 ^d^	0.092 ^abc^	0.097 ^ab^	0.083 ^bcd^	0.104 ^a^
EE24	Ethyl pentadecanoate	MS, LRI	2154	2151	5.259	0.307 ^bc^	0.355 ^bc^	0.273 ^c^	0.357 ^bc^	0.572 ^a⁎^	0.331 ^bc^	0.438 ^b^
EE25	Ethyl octadecanoate	MS, LRI	2463	2464	4.891	0.401 ^bc^	0.606 ^ab⁎^	0.227 ^c⁎^	0.504 ^b^	0.809 ^a^	0.610 ^ab⁎^	0.457 ^bc^
EE26	Ethyl lactate (mg/L) ^‡^	SC, MS, LRI	1341	1341	4.481	11.66 ^ab^	11.40 ^ab^	12.58 ^a⁎^	11.52 ^ab^	10.05 ^bc⁎^	12.80 ^a^	9.16 ^c⁎^
EE27	Ethyl nonanoate	MS, LRI	1537	1535	4.400	6.19 ^cd^	8.04 ^abc^	4.77 ^d^	6.37 ^bcd^	8.15 ^ab^	6.54 ^bcd^	8.53 ^a^
EE28	Ethyl tetradecanoate	MS, LRI	2054	2054	4.109	9.49 ^bc^	11.41 ^bc^	5.30 ^c⁎^	17.17 ^ab⁎^	25.94 ^a^	10.36 ^bc^	10.44 ^bc^
EE29	Ethyl dodecanoate ^‡^	MS, LRI	1843	1843	3.950	0.477 ^c^	0.828 ^ab⁎^	0.422 ^c^	0.814 ^ab⁎^	0.963 ^a⁎^	0.913 ^a^	0.557 ^bc^
EE30	Ethyl octanoate (mg/L) ^‡^	SC, MS, LRI	1435	1435	3.805	0.84 ^cd^	0.91 ^bcd^	0.72 ^d^	1.08 ^abc^	1.08 ^abc^	1.27 ^a⁎^	1.20 ^ab^
EE31	Ethyl 2-octenoate	MS, LRI	1559	1557	3.750	0.224 ^a^	0.207 ^ab^	0.081 ^c⁎^	0.173 ^ab^	0.196 ^ab^	0.149 ^bc^	0.197 ^ab^
EE32	Ethyl undecanoate	MS, LRI	1747	1739	3.600	0.434 ^bc^	0.541 ^abc^	0.294 ^c^	0.365 ^c^	0.819 ^a⁎^	0.439 ^bc^	0.668 ^ab^
EE33	Ethyl *cis*-4-octenoate	MS	1471	–	3.505	0.032 ^c^	0.047 ^bc^	0.032 ^c^	0.087 ^ab^	0.094 ^ab^	0.067 ^abc^	0.102 ^a⁎^
EE34	Ethyl hexanoate (mg/L) ^‡^	SC, MS, LRI	1242	1236	3.377	0.93 ^abc^	0.83 ^bc⁎^	0.81 ^c⁎^	0.87 ^bc^	0.86 ^bc^	0.95 ^ab^	1.04 ^a^
EE35	Ethyl pyruvate	MS, LRI	1271	1267	3.281	50.52 ^abc^	41.80 ^bc^	61.92 ^ab^	62.78 ^ab^	68.89 ^a^	30.88 ^c^	45.06 ^bc^
EE36	Ethyl hexadecanoate	MS, LRI	2251	2241	3.092	22.72 ^bc^	26.70 ^ab^	13.89 ^c⁎^	25.25 ^ab^	34.08 ^a^	22.37 ^bc^	20.77 ^bc^
EE37	Ethyl tridecanoate	MS, LRI	1947	1950	2.886	0.264 ^b^	0.333 ^b^	0.240 ^b^	0.253 ^b^	0.579 ^a⁎^	0.289 ^b^	0.449 ^ab^
EE38	Ethyl heptanoate	MS, LRI	1340	1342	2.750	10.05 ^abc^	11.42 ^a⁎^	10.80 ^ab^	8.04 ^c⁎^	11.05 ^ab^	8.99 ^bc^	9.21 ^abc^
EE39	Ethyl 4-hexenoate II ^‡^	MS LRI	1300	1292	2.490	1.02 ^abc^	1.87 ^ab^	1.92 ^a^	0.91 ^abc^	0.82 ^bc^	0.83 ^bc^	0.42 ^c⁎^
EE40	Ethyl decanoate (mg/L) ^‡^	S, MS, LRI	1637	1638	2.471	0.36 ^c^	0.44 ^bc^	0.40 ^bc^	0.58 ^a⁎^	0.48 ^abc^	0.46 ^abc^	0.50 ^ab^
EE41	Ethyl *cis*-11-hexadecenoate	MS, LRI	2281	2236	2.352	0.343 ^b^	0.450 ^ab^	0.365 ^b^	0.388 ^b^	0.611 ^a^	0.391 ^b^	0.333 ^b^
EE42	Ethyl *trans*-4-decenoate	MS, LRI	1672	1680	1.971	0.398 ^ab^	0.337 ^b^	0.240 ^b^	0.576 ^a^	0.392 ^ab^	0.320 ^b^	0.434 ^ab^
EE43	Ethyl 9-decenoate	S, MS, LRI	1697	1697	1.929	0.43 ^b^	1.41 ^ab^	0.37 ^b^	1.28 ^ab⁎^	2.02 ^a^	0.68 ^b^	0.92 ^ab⁎^
EE44	Ethyl 7-octenoate	MS, LRI	1491	1486	1.520	0.337 ^ab^	0.429 ^ab^	0.318 ^b^	0.457 ^ab^	0.506 ^ab⁎^	0.309 ^b^	0.563 ^a⁎^
	*Acetate esters*											
AE1	3-Ethoxypropyl acetate	MS	1361	–	286.143	4.92 ^b^	0.66 ^cd⁎^	9.90 ^a⁎^	0.15 ^d⁎^	0.21 ^d⁎^	0.99 ^⁎^	0.28 ^d⁎^
AE2	Diol acetate n.i.	MS	1741	–	159.362	42.82 ^a^	27.18 ^b⁎^	15.49 ^c⁎^	14.55 ^c⁎^	13.32 ^c⁎^	15.45 ^c⁎^	13.73 ^c⁎^
AE3	*trans*-3-Hexen-1-yl acetate ^‡^	MS LRI	1313	1316	85.144	73.2 ^d^	77.8 ^cd⁎^	120.3 ^b⁎^	85.8 ^c⁎^	141.4 ^a⁎^	75.0 ^d^	113.9 ^b⁎^
AE4	Isopropyl acetate ^‡^	MS, LRI	<1000	901	84.294	54.91 ^b^	38.09 ^d⁎^	88.61 ^a⁎^	36.33 ^d⁎^	41.64 ^cd⁎^	44.48 ^c⁎^	47.28 ^c⁎^
AE5	*cis*-3-Hexen-1-yl acetate ^‡^	MS, LRI	1304	1300	79.568	95.7 ^d^	105.9 ^d⁎^	218.9 ^a⁎^	123.9 ^c⁎^	173.9 ^b⁎^	110.9 ^cd⁎^	171.9 ^b⁎^
AE6	Pentyl acetate	S, MS, LRI	1169	1185	55.273	3.94 ^e^	4.55 ^e⁎^	14.21 ^a⁎^	4.89 ^de⁎^	6.80 ^c⁎^	6.41 ^cd⁎^	10.90 ^b⁎^
AE7	Isoamyl acetate (mg/L) ^‡^	SC, MS, LRI	1133	1133	51.536	3.50 ^e^	4.55 ^d⁎^	8.29 ^a⁎^	5.35 ^c⁎^	5.69 ^c⁎^	5.75 ^c⁎^	6.46 ^b⁎^
AE8	Butyl acetate	MS, LRI	<1100	1064	46.877	17.50 ^d^	23.75 ^cd⁎^	77.89 ^a⁎^	21.92 ^cd⁎^	28.56 ^c⁎^	44.65 ^b⁎^	49.96 ^b⁎^
AE9	1,2,4-Butanetriol 1,4-diacetate	MS	1450	–	45.363	0.110 ^c^	0.065 ^d^	0.000 ^e⁎^	0.099 ^cd^	0.170 ^b⁎^	0.189 ^b⁎^	0.263 ^a⁎^
AE10	2-Phenethyl acetate ^‡^	SC, MS, LRI	1803	1801	45.204	166.1 ^bc^	402.1 ^a⁎^	439.3 ^a⁎^	212.0 ^b⁎^	156.3 ^bc^	112.0 ^c⁎^	195.5 ^b^
AE11	Isobutyl acetate ^‡^	SC, MS, LRI	1015	1009	43.581	81.7 ^d^	145.3 ^c⁎^	239.4 ^a⁎^	143.3 ^c⁎^	146.5 ^c⁎^	186.1 ^b⁎^	182.5 ^b⁎^
AE12	Ethyl acetate (mg/L) ^¤^	SC, MS, LRI	<1100	890	35.583	43.40 ^cd^	36.68 ^d^	75.36 ^a⁎^	42.66 ^d^	49.75 ^c⁎^	57.75 ^b⁎^	62.64 ^b⁎^
AE13	Hexyl acetate ^‡^	SC, MS, LRI	1272	1272	21.825	293.9 ^de^	321.5 ^cd⁎^	394.1 ^a⁎^	361.0 ^bc⁎^	340.7 ^c⁎^	277.3 ^e⁎^	379.6 ^ab⁎^
AE14	1,3-Butanediol diacetate	MS, LRI	1785	1768	18.387	1.19 ^b^	1.26 ^b^	1.52 ^a^	0.78 ^cd⁎^	0.60 ^d⁎^	0.82 ^cd⁎^	0.91 ^c⁎^
AE15	*cis*-6-Nonen-1-yl acetate	MS, LRI	1635	1634	16.523	0.263 ^b^	0.198 ^b^	0.097 ^c⁎^	0.268 ^b^	0.101 ^c⁎^	0.077 ^c⁎^	0.404 ^a^
AE16	Thujyl acetate	MS	1735	–	13.698	0.301 ^b^	0.256 ^b^	0.518 ^a⁎^	0.302 ^b^	0.292 ^b^	0.290 ^b^	0.455 ^a⁎^
AE17	2-Ethylbutyl acetate	MS, LRI	1238	1205	9.871	6.24 ^d^	12.30 ^b⁎^	16.74 ^a⁎^	7.83 ^cd^	6.11 ^d^	7.28 ^d^	11.13 ^bc⁎^
AE18	3-Methyl-1,4-pentadien-3-yl acetate	MS	1440	–	9.762	0.083 ^cde^	0.067 ^de^	0.173 ^a⁎^	0.098 ^cd^	0.124 ^bc⁎^	0.039 ^e⁎^	0.143 ^ab^
AE19	*trans*-Penten-1-yl acetate	MS, LRI	1224	1228	9.182	0.76 ^cd^	0.89 ^bc^	1.14 ^a⁎^	0.71 ^d⁎^	1.14 ^a⁎^	0.89 ^bc⁎^	0.97 ^b⁎^
AE20	Heptyl acetate	MS, LRI	1382	1374	8.999	10.24 ^bc^	12.72 ^b⁎^	13.06 ^b^	13.39 ^b⁎^	10.43 ^bc^	8.22 ^c^	18.28 ^a⁎^
AE21	(*trans*,*trans*)-2,4-Octadienyl acetate	MS	1570	–	8.117	0.212 ^ab^	0.239 ^a^	0.262 ^a^	0.220 ^ab^	0.181 ^b^	0.119 ^c⁎^	0.265 ^a^
AE22	Methyl acetate ^‡^	MS, LRI	<1000	813	6.369	13.70 ^b^	14.42 ^b^	19.45 ^a⁎^	21.89 ^a⁎^	19.93 ^a⁎^	18.50 ^a⁎^	19.96 ^a⁎^
AE23	2-Ethenylphenyl acetate	MS	1990	–	4.865	0.074 ^bc^	0.103 ^a⁎^	0.084 ^b^	0.082 ^b^	0.078 ^bc^	0.059 ^c⁎^	0.064 ^bc^
AE24	3-Hepten-1-yl acetate	MS	1408	–	4.499	0.485 ^bc^	0.660 ^ab^	0.877 ^a^	0.716 ^ab^	0.579 ^ab^	0.171 ^c^	0.882 ^a^
AE25	3-Methyl-3-buten-1-yl acetate	MS, LRI	1188	1190	4.050	0.452 ^bcd^	0.359 ^bcd^	0.828 ^a^	0.169 ^d^	0.278 ^cd^	0.500 ^abc^	0.614 ^ab^
AE26	Phenylmethyl acetate	MS, LRI	1736	1743	3.138	0.153 ^c^	0.151 ^c^	0.205 ^a^	0.153 ^c^	0.138 ^c^	0.158 ^bc^	0.197 ^ab^
AE27	3-Methylheptyl acetate	MS, LRI	1387	1395	2.238	0.424 ^ab^	0.622 ^a^	0.665 ^a^	0.670 ^a⁎^	0.301 ^b^	0.522 ^ab^	0.581 ^a^
AE28	Octyl acetate ^‡^	MS, LRI	1481	1483	2.137	1.60 ^b^	1.10 ^b^	3.36 ^a^	1.45 ^b^	1.28 ^b^	1.03 ^b^	1.64 ^b^
AE29	1-Octen-3-yl acetate	MS, LRI	1382	1379	1.935	0.427 ^b^	0.520 ^ab^	0.883 ^a^	0.408 ^b^	0.524 ^ab^	0.405 ^b^	0.853 ^ab⁎^
AE30	Propyl acetate	MS, LRI	<1100	982	1.283	54.3	33.8	94.9	27.1	31.0	43.4	105.1
	*Other esters*											
OE1	2-Phenylethyl formate	MS, LRI	1797	1806	54.676	0.93 ^b^	1.38 ^a^^^⁎^^	0.65 ^de⁎^	0.80 ^c^	0.70 ^cd⁎^	0.55 ^e⁎^	0.61 ^de⁎^
OE2	2-Methylbutyl 2-methylbutyrate	MS, LRI	1282	1277	53.942	0.281 ^b^	0.914 ^a⁎^	0.103 ^cd⁎^	0.287 ^b^	0.313 ^b^	0.214 ^bc⁎^	0.074 ^d^
OE3	2-Phenylethyl isobutyrate	MS, LRI	1891	1896	48.626	0.92 ^b^	2.15 ^a⁎^	0.61 ^c⁎^	0.56 ^c⁎^	0.43 ^c⁎^	0.52 ^c⁎^	0.43 ^c⁎^
OE4	Methyl pyruvate	MS	1779	–	43.220	19.14 ^a^	15.27 ^b⁎^	5.73 ^e⁎^	18.41 ^a^	12.71 ^c⁎^	8.82 ^d⁎^	11.05 ^cd⁎^
OE5	Isobutyl octanoate	MS, LRI	1553	1551	35.751	0.63 ^d^	1.00 ^c⁎^	0.80 ^cd^	1.98 ^a⁎^	2.21 ^a⁎^	1.58 ^b⁎^	2.16 ^a⁎^
OE6	Isoamyl isovalerate	MS, LRI	1298	1294	25.136	0.421 ^b^	0.730 ^a⁎^	0.278 ^d⁎^	0.393 ^bc^	0.386 ^bc^	0.322 ^cd^	0.292 ^d⁎^
OE7	Isoamyl propanoate	MS, LRI	1188	1188	24.146	3.813 ^b^	5.154 ^a^	2.763 ^c⁎^	1.501 ^d⁎^	1.833 ^d⁎^	1.274 ^⁎^	1.243 ^d⁎^
OE8	Diethyl malate	MS, LRI	2047	2048	22.880	1.55 ^b^	1.35 ^b^	1.43 ^b^	1.86 ^a^	1.50 ^b^	1.47 ^b^	0.51 ^c⁎^
OE9	Isoamyl octanoate	MS, LRI	1660	1657	22.391	34.52 ^d^	46.14 ^c⁎^	19.69 ^e⁎^	55.15 ^ab⁎^	56.15 ^a⁎^	37.27 ^d^	47.57 ^bc⁎^
OE10	Methyl octanoate	MS, LRI	1398	1399	16.685	81.8 ^c^	82.1 ^c^	45.04 ^d⁎^	93.4 ^bc^	100.8 ^ab⁎^	81.3 ^c^	116.4 ^a⁎^
OE11	2-Phenylethyl isovalerate	MS, LRI	1997	1983	15.476	0.096 ^b^	0.150 ^a⁎^	0.086 ^b^	0.148 ^a⁎^	0.101 ^b^	0.097 ^b^	0.098 ^b^
OE12	Hexyl propyl oxalate	MS	1525	–	14.362	0.72 ^b^	1.02 ^a⁎^	0.58 ^c^^⁎^	0.70 ^bc^	0.66 ^bc^	0.63 ^bc^	0.92 ^a⁎^
OE13	Methyl decanoate	MS, LRI	1598	1599	14.264	10.50 ^bc^	9.93 ^c^	5.93 ^d⁎^	12.69 ^b^	12.83 ^b⁎^	9.33 ^c^	15.34 ^a⁎^
OE14	Propyl hexanoate	MS, LRI	1324	1319	13.388	2.83 ^a^	1.96 ^bc⁎^	2.30 ^b^	1.19 ^d⁎^	1.65 ^c⁎^	1.75 ^c⁎^	2.32 ^b^
OE15	2-Phenylethyl 2-methylbutyrate	MS, LRI	1968	1864	13.258	1.65 ^b^	2.58 ^a⁎^	1.66 ^b^	1.60 ^bc^	1.03 ^d⁎^	1.16 ^cd⁎^	1.10 ^d⁎^
OE16	Diethyl succinate ^‡^	MS, LRI	1677	1669	13.043	397.6 ^cd^	585.2 ^a⁎^	503.0 ^b⁎^	469.4 ^bc^	435.1 ^bcd^	388.7 ^de^	317.9 ^e^
OE17	Diethyl glutarate	MS, LRI	1785	1780	11.745	0.249 ^a^	0.191 ^bc⁎^	0.178 ^c⁎^	0.236 ^a^	0.199 ^bc⁎^	0.199 ^bc⁎^	0.145 ^d⁎^
OE18	2-Phenylethyl hexanoate	MS, LRI	2176	2166	11.179	0.589 ^b^	0.716 ^a^	0.361 ^d⁎^	0.558 ^bc^	0.468 ^c⁎^	0.435 ^cd⁎^	0.507 ^bc^
OE19	Propyl octanoate	MS, LRI	1525	1530	10.906	1.91 ^b^	1.47 ^cd⁎^	1.28 ^d⁎^	1.44 ^cd⁎^	1.71 ^bc⁎^	1.59 ^bcd^	2.36 ^a^
OE20	Isoamyl hexanoate	S, MS, LRI	1466	1458	9.455	29.45 ^b^	40.16 ^a⁎^	19.76 ^c⁎^	35.41 ^ab⁎^	37.59 ^a⁎^	29.69 ^b^	34.27 ^ab^
OE21	Isobutyl hexanoate	MS, LRI	1357	1357	9.323	2.36 ^c^	3.49 ^bc⁎^	3.34 ^bc^	4.52 ^ab⁎^	5.73 ^a⁎^	5.06 ^a⁎^	5.55 ^a⁎^
OE22	Isoamyl lactate	MS, LRI	1570	1580	8.770	2.17 ^bc^	3.13 ^a⁎^	2.21 ^bc^	2.37 ^bc^	1.99 ^cd^	2.48 ^b^	1.60 ^d⁎^
OE23	Ester n.i.	MS	1210	–	8.705	20.71 ^bc^	34.79 ^ab^	6.71 ^c⁎^	9.10 ^c⁎^	50.61 ^a⁎^	21.18 ^bc^	34.67 ^ab^
OE24	Hexadecyl hexanoate	MS	1990	–	8.686	0.063 ^b^	0.086 ^b⁎^	0.262 ^a⁎^	0.216 ^a⁎^	0.204 ^a⁎^	0.203 ^a⁎^	0.196 ^a⁎^
OE25	Diethyl fumarate	MS, LRI	1654	1647	8.397	0.191 ^b^	0.229 ^a⁎^	0.192 ^b^	0.235 ^a⁎^	0.241 ^a⁎^	0.188 ^b^	0.181 ^b^
OE26	Isobutyl decanoate	MS, LRI	1760	1756	8.295	0.100 ^d^	0.159 ^cd⁎^	0.140 ^cd^	0.254 ^b⁎^	0.359 ^a⁎^	0.227 ^bc⁎^	0.260 ^b⁎^
OE27	Diethyl malonate	MS, LRI	1581	1582	8.130	0.503 ^bc^	0.473 ^bc^	0.456 ^c^	0.690 ^a^	0.722 ^a⁎^	0.575 ^b⁎^	0.572 ^b^
OE28	2-Phenylethyl octanoate	MS, LRI	2388	2373	6.620	1.06 ^b^	1.36 ^a⁎^	0.73 ^c⁎^	1.22 ^ab^	1.05 ^b^	1.00 ^b^	1.04 ^b^
OE29	Isoamyl buyrate ^‡^	MS, LRI	1262	1266	5.970	7.65 ^ab^	8.61 ^a⁎^	4.67 ^c⁎^	7.17 ^ab^	6.33 ^b⁎^	6.82 ^b^	6.79 ^b^
OE30	Amyl methacrylate	MS	2047	–	5.440	0.219 ^a^	0.111 ^b⁎^	0.090 ^b^	0.135 ^b^	0.127 ^b^	0.091 ^b⁎^	0.098 ^b⁎^
OE31	Methyl hexanoate	S, MS, LRI	1181	1188	4.850	5.50 ^ab^	6.67 ^a^	3.74 ^bc⁎^	3.21 ^c⁎^	2.98 ^c⁎^	2.74 ^c⁎^	3.99 ^bc^
OE32	Ethyl hydrogen succinate	MS, LRI	2380	2367	4.580	106.8 ^a^	120.8 ^a^	91.9 ^ab^	65.3 ^bc^	88.0 ^ab^	54.8 ^c⁎^	72.7 ^bc^
OE33	Isoamyl decanoate	MS, LRI	1866	1864	3.697	19.12 ^bc^	23.17 ^ab^	11.93 ^c⁎^	23.32 ^ab^	31.68 ^a^	18.91 ^bc^	19.84 ^bc^
OE34	Isoamyl dodecanoate	MS, LRI	2069	2071	3.657	1.03 ^bc^	1.37 ^bc^	0.55 ^c⁎^	1.20 ^bc^	2.28 ^a^	1.47 ^ab^	1.01 ^bc^
OE35	Methyl dodecanoate	MS, LRI	1810	1806	3.118	0.251 ^bc^	0.349 ^ab^	0.135 ^c⁎^	0.332 ^ab^	0.473 ^a^	0.333 ^ab^	0.339 ^ab^
OE36	Propyl decanoate	MS, LRI	1729	1743	2.718	0.459 ^ab^	0.414 ^b^	0.381 ^b^	0.390 ^b^	0.471 ^ab^	0.385 ^b^	0.568 ^a^
OE37	Diethyl 2-hydroxyglutarate	MS, LRI	2161	2195	2.163	0.377 ^ab^	0.309 ^b^	0.338 ^ab^	0.430 ^a^	0.378 ^ab^	0.287 ^b^	0.277 ^b^
OE38	Triethyl citrate	MS, LRI	2463	2461	2.070	0.065 ^abc^	0.073 ^ab^	0.038 ^bc^	0.041 ^abc^	0.077 ^a^	0.036 ^c^	0.058 ^abc^
OE39	Isopropyl decanoate	MS	2235	–	1.815	0.388 ^ab^	0.305 ^b^	0.537 ^ab^	0.682 ^a^	0.319 ^b^	0.386 ^ab^	0.383 ^ab^
OE40	Isobutyl butyrate	MS, LRI	1155	1159	1.069	0.250	0.428	0.403	0.452	0.433	0.467	0.470
OE41	Hexyl propanoate	MS, LRI	1345	1342	0.867	0.499	0.643 ^⁎^	0.251 ^⁎^	0.147 ^⁎^	0.173 ^⁎^	0.180 ^⁎^	1.866
OE42	2-Ethyl-1-hexyl propanoate	MS	1455	–	0.544	0.94	1.10	0.82	1.25	0.95	1.00	1.04
OE43	Butyl butyrate	MS, LRI	1219	1219	0.505	1.05	1.30	2.21	1.30	1.75	1.22	1.36
	*Sulfur containing compounds*											
SU1	3-Hydroxyethyl 3-hydroxypropyl sulfide I	MS	1779	–	146.469	0.180 ^d^	0.930 ^b⁎^	1.559 ^a⁎^	0.510 ^c⁎^	0.536 ^c⁎^	0.949 ^b⁎^	0.507 ^c⁎^
SU2	3-Hydroxyethyl 3-hydroxypropyl sulfide II	MS, LRI	1822	1825	123.608	0.101 ^e^	0.853 ^b⁎^	0.947 ^a⁎^	0.392 ^d⁎^	0.406 ^d⁎^	0.549 ^c⁎^	0.466 ^d⁎^
SU3	3-Methionyl acetate	MS, LRI	1635	1627	61.646	1.75 ^d^	3.47 ^b⁎^	6.37 ^a⁎^	3.05 ^bc⁎^	2.17 ^d⁎^	3.00 ^bc⁎^	2.86 ^c⁎^
SU4	Mercapto-2-propanone	MS, LRI	1377	1359	60.887	0.318 ^b^	0.042 ^c⁎^	0.963 ^a⁎^	0.000 ^c⁎^	0.086 ^c^	0.000 ^c⁎^	0.045 ^c⁎^
SU5	Ethyl 3-methylthiopropanoate	MS, LRI	1570	1577	47.646	3.74 ^a^	3.98 ^a^	2.53 ^c⁎^	2.98 ^b⁎^	2.24 ^cd⁎^	1.86 ^d⁎^	1.29 ^e⁎^
SU6	2-Thiophenecarboxaldehyde	S, MS, LRI	1704	1701	43.062	0.313 ^b^	0.373 ^a⁎^	0.169 ^cd⁎^	0.205 ^c⁎^	0.174 ^cd⁎^	0.144 ^d⁎^	0.137 ^d⁎^
SU7	Dihydro-2-methyl-3(2H)-thiophenone ^‡^	MS, LRI	1512	1506	42.031	2.79 ^c^	1.48 ^d⁎^	8.13 ^a⁎^	5.64 ^b⁎^	5.52 ^b⁎^	5.44 ^b⁎^	3.69 ^c⁎^
SU8	Methionol	S, MS, LRI	1722	1717	34.023	17.25 ^c^	26.54 ^a⁎^	18.64 ^c^	23.72 ^b⁎^	16.96 ^cd^	14.60 ^de⁎^	13.57 ^e⁎^
SU9	S-ethyl octanethioate	MS	1525	–	30.629	5.34 ^c^	1.14 ^d⁎^	5.70 ^c^	2.29 ^d⁎^	5.68 ^c^	10.77 ^b⁎^	16.67 ^a⁎^
SU10	Ethyl thiophene-2-carboxylate	MS	1772	–	16.127	0.060 ^d^	0.093 ^ab⁎^	0.040 ^e⁎^	0.096 ^a⁎^	0.078 ^bc^	0.072 ^cd^	0.098 ^a⁎^
SU11	Methional	MS, LRI	1465	1461	13.742	0.088 ^b^	0.252 ^a⁎^	0.116 ^b^	0.242 ^a⁎^	0.125 ^b^	0.102 ^b^	0.105 ^b^
SU12	Propyl ethynyl sulfoxide	MS	1559	–	10.748	1.16 ^c^	3.18 ^a⁎^	1.81 ^bc⁎^	3.05 ^a⁎^	1.87 ^b^	2.07 ^b⁎^	1.95 ^b⁎^
SU13	Benzothiazole	MS, LRI	1965	1962	8.174	0.614 ^bc^	0.693 ^b^	0.607 ^bc^	0.626 ^bc^	0.690 ^b^	0.596 ^c^	0.839 ^a⁎^
SU14	2-(Methylmercapto) benzothiazole ^‡^	MS, LRI	2433	2422	3.555	1.87 ^abc^	0.85 ^bc^	0.62 ^c^	2.87 ^a^	0.86 ^bc^	2.10 ^ab^	1.17 ^bc^
SU15	2-Methyltetrahydrothiophen-3-one	MS, LRI	1531	1538	3.327	28.02 ^cd^	26.32 ^d^	37.42 ^ab⁎^	39.79 ^a^	30.80 ^bcd^	37.99 ^ab⁎^	35.18 ^abc^
SU16	Ethyl methanesulfonate	MS	1691	–	2.554	1.38 ^a^	1.32 ^ab^	1.18 ^b^	1.45 ^a^	1.49 ^a^	1.36 ^ab^	1.32 ^ab^
SU17	Isothiocyanatocyclohexane	MS, LRI	1679	1670	2.263	0.667 ^b^	0.851 ^a⁎^	0.705 ^b^	0.792 ^ab^	0.704 ^b^	0.723 ^b^	0.710 ^b^
SU18	4-(Methylthio)-1-butanol	MS	1841	**–**	1.055	0.048 ^ab^	0.049 ^ab^	0.044 ^b^	0.054 ^a^	0.049 ^ab^	0.048 ^ab^	0.050 ^ab^
	*Furanoids and lactones*											
FL1	γ-Butyrolactone	MS, LRI	1635	1644	28.741	35.87 ^a^	21.73 ^e⁎^	22.59 ^e⁎^	29.58 ^bc⁎^	26.79 ^cd⁎^	30.66 ^b⁎^	23.99 ^de⁎^
FL2	2-Butyltetrahydrofuran	MS	1271	–	25.642	28.50 ^b^	33.63 ^a^	11.76 ^d⁎^	19.24 ^c⁎^	21.23 ^c⁎^	18.09 ^c⁎^	18.35 ^c⁎^
FL3	γ-Decalactone	MS, LRI	2154	2152	10.478	2.02 ^bc^	2.27 ^bc^	1.97 ^c^	2.58 ^b^	1.95 ^c^	3.74 ^a⁎^	2.31 ^bc^
FL4	γ-Nonalactone	S, MS, LRI	2040	2046	10.145	4.05 ^b^	3.90 ^b^	4.15 ^b^	5.71 ^a⁎^	3.47 ^b^	3.70 ^b^	4.10 ^b^
FL5	4-(1-Hydroxyethyl)-γ-butyrolactone	MS, LRI	2388	2328	9.481	1.87 ^d^	4.54 ^cd⁎^	9.40 ^a⁎^	6.20 ^bc⁎^	9.32 ^a⁎^	5.56 ^c⁎^	8.63 ^ab⁎^
FL6	β-Methyl-γ-butyrolactone	MS	1816	–	7.828	1.43 ^d^	1.55 ^cd^	2.01 ^b⁎^	1.95 ^b⁎^	1.88 ^b⁎^	2.35 ^a⁎^	1.88 ^bc⁎^
FL7	Pantolactone	MS, LRI	2040	2034	7.644	0.387 ^bc^	0.678 ^a⁎^	0.464 ^b^	0.426 ^b^	0.465 ^b^	0.286 ^c^	0.371 ^bc^
FL8	2,2,4-Trimethyl-5-(2,2-dimethylpropyl)-3(2H)-furanone	MS	1570	–	4.694	0.85 ^d^	1.52 ^abc⁎^	1.35 ^bcd⁎^	1.85 ^ab⁎^	1.56 ^abc^	1.91 ^a⁎^	1.22 ^cd^
FL9	Ethyl 2-furoate	MS, LRI	1629	1628	3.884	24.93 ^a^	28.54 ^a⁎^	18.08 ^b⁎^	26.14 ^a^	28.37 ^a^	27.55 ^a⁎^	29.42 ^a^
FL10	Solerone	MS, LRI	2076	2096	3.531	1.34 ^abc^	1.61 ^a^	1.01 ^c^	1.15 ^bc^	1.36 ^ab^	1.02 ^bc^	1.23 ^bc^
FL11	γ-Undecalactone	MS, LRI	2235	2235	3.371	6.12 ^b^	5.38 ^b^	6.03 ^b^	9.35 ^a^	7.53 ^ab^	5.64 ^b^	6.24 ^b^
FL12	γ-Octalactone	S, MS, LRI	1926	1924	2.967	4.36 ^a^	4.19 ^a^	3.33 ^b⁎^	4.48 ^a^	4.21 ^a^	4.25 ^a^	4.53 ^a^
FL13	Furfural	S, MS, LRI	1466	1460	2.596	3.01 ^ab^	3.38 ^a^	3.10 ^ab^	3.08 ^ab^	2.61 ^b^	2.57 ^b^	2.77 ^b^
FL14	γ-Ethoxybutyrolactone	MS, LRI	1735	1728	2.363	0.235 ^b^	0.240 ^b^	0.284 ^ab^	0.328 ^a^	0.223 ^b^	0.245 ^b^	0.234 ^b^
FL15	2-Furancarboxylic acid	MS	2446	–	2.241	0.232 ^bcd^	0.311 ^bcd^	0.314 ^abcd^	0.389 ^abcd^	0.297 ^bcd^	0.266 ^bcd^	0.484 ^a^
FL16	4-Methyl-2-butenoic acid γ-lactone	MS, LRI	1897	1909	2.138	0.432 ^ab^	0.544 ^a^	0.391 ^b^	0.515 ^a^	0.476 ^ab^	0.516 ^a^	0.544 ^a^
FL17	δ-Hexalactone	MS, LRI	1804	1798	2.014	1.99 ^a^	1.53 ^ab^	1.38 ^b^	1.65 ^ab^	1.81 ^ab^	1.91 ^a^	1.96 ^a^
FL18	α-Methyl-γ-crotonolactone	MS, LRI	1729	1726	1.906	0.194 ^b^	0.193 ^b^	0.183 ^b^	0.219 ^ab^	0.212 ^ab^	0.203 ^ab^	0.237 ^a^
FL19	γ-Crotonolactone	MS, LRI	1766	1758	1.872	0.503 ^ab^	0.454 ^ab^	0.419 ^b^	0.510 ^a^	0.510 ^a^	0.496 ^ab^	0.537 ^a^
FL20	2-Pentylfuran	MS, LRI	1229	1231	1.577	1.22 ^ab^	1.40 ^a^	1.25 ^ab^	1.19 ^ab^	1.23 ^ab^	1.11 ^b^	1.19 ^ab^
FL21	β-Dihydroagarafuran	MS, LRI	1691	1704	1.279	0.215 ^ab^	0.232 ^a^	0.201 ^ab^	0.184 ^ab^	0.200 ^ab^	0.232 ^a^	0.110 ^b^
FL22	2-Hydroxy-γ-butyrolactone	MS, LRI	2176	2142	0.991	0.95	0.78	0.80	0.81 ^⁎^	1.08	0.72	1.11
FL23	2,5-Furandicarboxaldehyde	MS, LRI	1991	1991	0.910	0.092	0.095	0.090	0.104	0.092	0.088	0.098
FL24	δ-Lactone n.i. I	MS	1891	–	0.902	0.065	0.051	0.051	0.014	0.090	0.105	0.095
FL25	γ-Dodecalactone	MS, LRI	2380	2384	0.823	0.219	0.199	0.262	0.210	0.212	0.220	0.266
FL26	δ-Lactone n.i. II	MS	2090	–	0.721	0.130	0.060	0.077	0.037	0.084	0.116	0.106
FL27	2-Furancarboxaldehyde	MS, LRI	2505	2501	0.716	0.106	0.087	0.081	0.079	0.094	0.079 ^⁎^	0.095
FL28	2-Octylfuran	MS, LRI	1542	1530	0.682	0.230	0.309	0.195	0.277	0.294	0.330	0.251
	*Benzenoids*											
BE1	Benzeneacetaldehyde	S, MS, LRI	1654	1656	63.453	52.62 ^b^	84.97 ^a⁎^	38.96 ^cd⁎^	52.00 ^b^	41.91 ^c⁎^	34.59 ^d⁎^	37.35 ^cd⁎^
BE2	Benzenoid n.i.	MS	2187	–	57.407	0.125 ^b^	3.891 ^a⁎^	0.014 ^b^	0.020 ^b^	0.092 ^b^	0.000 ^b^	0.308 ^b^
BE3	Ethyl benzeneacetate	MS, LRI	1791	1788	40.729	15.51 ^b^	22.50 ^a⁎^	9.84 ^d⁎^	13.22 ^c^	10.35 ^d⁎^	10.15 ^d⁎^	8.87 ^d⁎^
BE4	Ethyl phenethyl ether	MS	1528	**–**	37.873	1.23 ^b^	1.99 ^a⁎^	0.99 ^c^	1.22 ^b^	1.02 ^c^	0.98 ^c⁎^	0.97 ^c⁎^
BE5	Acetaldehyde ethyl phenethyl acetal	MS	1891	–	37.817	0.239 ^b^	0.491 ^a⁎^	0.177 ^cd^	0.238 ^bc^	0.155 ^d⁎^	0.154 ^d⁎^	0.134 ^d⁎^
BE6	2-(2-Phenylethoxy)propanal	MS	1570	–	36.006	0.097 ^b^	0.265 ^a⁎^	0.035 ^cd^	0.099 ^b^	0.060 ^bc^	0.048 ^cd^	0.006 ^d⁎^
BE7	(2-Methylphenyl) methanol, 1-methylpropyl ether	MS	2596	**–**	32.189	0.277 ^b^	1.209 ^a⁎^	0.080 ^b⁎^	0.140 ^b⁎^	0.079 ^b⁎^	0.070 ^b⁎^	0.073 ^b⁎^
BE8	*p*-Ethylstyrene	MS, LRI	1461	1462	11.826	0.655 ^bc^	0.624 ^c^	0.410 ^d⁎^	0.701 ^abc^	0.789 ^a^	0.593 ^c^	0.746 ^ab^
BE9	Cardene	MS, LRI	1271	1269	8.755	2.98 ^b^	3.82 ^a⁎^	2.45 ^bc^	2.79 ^bc^	2.60 ^bc^	2.29 ^c⁎^	2.27 ^c^
BE10	1-Phenyl-3-phenethylundecane	MS	1954	–	6.002	0.668 ^ab^	0.691 ^ab^	0.513 ^c^	0.723 ^a^	0.732 ^a^	0.606 ^bc^	0.744 ^a^
BE11	Hexylbenzene	MS, LRI	1525	1524	4.904	0.646 ^abc^	0.603 ^bcd^	0.537 ^d^	0.673 ^ab^	0.670 ^ab^	0.577 ^cd^	0.708 ^a^
BE12	3,3-Dimethoxy-1-phenylpropane-1,2-dione	MS	1471	–	4.825	3.96	7.10 ^a⁎^	4.78 ^bc^	5.52 ^bc^	5.34 ^bc^	5.17 ^bc^	4.44 ^bc^
BE13	(4-Methylphenyl) methanol, neopentyl ether	MS	1968	–	4.688	0.347 ^ab^	0.340 ^ab^	0.258 ^c^	0.363 ^ab^	0.367 ^a^	0.306 ^bc^	0.385 ^a^
BE14	Benzeneacetic acid	MS, LRI	2566	2560	4.604	0.731 ^a^	0.746 ^a^	0.465 ^c^	0.539 ^bc^	0.650 ^ab^	0.478 ^c^	0.497 ^bc^
BE15	1-Methylnaphthalene	MS, LRI	1897	1893	4.396	0.140 ^bc^	0.163 ^a⁎^	0.131 ^c^	0.150 ^ab^	0.142 ^bc^	0.139 ^bc^	0.149 ^ab^
BE16	Acetophenone	S, MS, LRI	1660	1660	4.188	2.09 ^a^	2.01 ^ab^	1.67 ^c⁎^	2.00 ^ab^	1.83 ^bc⁎^	1.68 ^c⁎^	1.87 ^abc^
BE17	2-Ethyl-*m*-xylene	MS, LRI	1377	1372	4.049	1.10 ^a^	0.66 ^b⁎^	0.85 ^ab^	0.60 ^b⁎^	0.79 ^b^	0.61 ^b⁎^	0.57 ^b⁎^
BE18	Benzyl alcohol	S, MS, LRI	1879	1877	3.325	2.45 ^bc^	2.71 ^ab^	2.35 ^c^	2.83 ^a^	2.60 ^abc^	2.44 ^bc^	2.88 ^a⁎^
BE19	2,6-Dimethylstyrene	MS	1482	–	3.233	0.629 ^a^	0.578 ^ab^	0.496 ^b^	0.639 ^a^	0.669 ^a^	0.576 ^ab^	0.497 ^b⁎^
BE20	Phenyl ethyl ketone	MS, LRI	1735	1734	3.146	0.133 ^ab^	0.128 ^b^	0.116 ^b^	0.135 ^ab^	0.124 ^b^	0.117 ^b⁎^	0.150 ^a^
BE21	Styrene	MS, LRI	1258	1262	3.097	2.66 ^b^	4.97 ^a⁎^	3.10 ^b^	3.41 ^b^	2.80 ^b^	2.57 ^b^	2.34 ^b^
BE22	α,α-Dimethylbenzenemethanol	MS, LRI	1766	1770	2.904	0.093 ^abc^	0.087 ^bc^	0.093 ^abc^	0.095 ^ab^	0.086 ^bc^	0.081 ^c^	0.102 ^a^
BE23	(Phenylmethylene)-propanedial	MS	1872	–	2.851	0.388 ^ab^	0.387 ^ab^	0.351 ^b^	0.398 ^a^	0.401 ^a^	0.357 ^b^	0.403 ^a^
BE24	4-Ethylacetophenone	MS, LRI	1841	1867	2.826	0.203 ^ab^	0.210 ^a^	0.196 ^ab^	0.212 ^a^	0.210 ^a^	0.183 ^b⁎^	0.217 ^a^
BE25	Versalide	MS	2326	–	2.592	0.055 ^b^	0.070 ^b^	0.122 ^a^	0.082 ^ab^	0.038 ^b^	0.049 ^b^	0.060 ^b^
BE26	(3-Methylphenyl)methanol, 2-methylpropyl ether	MS	1756	**–**	2.589	0.489 ^ab^	0.466 ^b^	0.385 ^b^	0.405 ^b^	0.522 ^ab^	0.454 ^b^	0.615 ^a⁎^
BE27	Methyl salicylate	S, MS, LRI	1785	1789	2.543	2.16 ^c^	2.49 ^abc^	2.26 ^bc^	2.64 ^ab⁎^	2.34 ^abc^	2.25 ^c^	2.70 ^a⁎^
BE28	*p*-Cymene	MS, LRI	1435	1442	2.525	28.34 ^bc^	47.37 ^a^	17.93 ^c^	35.50 ^abc^	34.29 ^abc^	43.71 ^ab^	31.65 ^abc^
BE29	4-Phenylbutenone	MS	1997	–	2.517	0.210 ^a^	0.212 ^a^	0.186 ^bc^	0.212 ^a^	0.206 ^abc^	0.184 ^c^	0.208 ^ab^
BE30	4-Methylacetophenone	MS, LRI	1766	1763	2.315	0.139 ^ab^	0.139 ^ab^	0.126 ^b^	0.148 ^a^	0.142 ^ab^	0.139 ^ab^	0.157 ^a^
BE31	4-Ethylbenzaldehyde	MS, LRI	1716	1714	2.172	0.873 ^ab^	0.927 ^a^	0.809 ^b^	0.948 ^a^	0.903 ^ab^	0.857 ^ab^	0.915 ^ab^
BE32	3-(1-Methylethyl)benzoic acid	MS	2642	–	2.119	0.095 ^a^	0.018 ^b^	0.011 ^b^	0.005 ^b^	0.022 ^b^	0.025 ^ab*^	0.070 ^ab^
BE33	Cinnamaldehyde	MS, LRI	1841	1884	2.104	0.421 ^abc^	0.432 ^ab^	0.392 ^bc^	0.437 ^a^	0.422 ^abc^	0.384 ^c^	0.433 ^ab^
BE34	Ethylbenzene	MS, LRI	1129	1130	2.031	0.236 ^b^	0.355 ^ab^	0.537 ^ab⁎^	0.281 ^b^	0.242 ^b^	0.517 ^ab⁎^	0.745 ^a^
BE35	Durene	MS, LRI	1445	1435	2.015	2.24 ^ab^	2.04 ^ab^	1.94 ^ab^	1.82 ^b^	2.36 ^a^	1.84 ^b^	1.86 ^b^
BE36	*p*-Methoxyanisole	MS, LRI	1747	1752	2.015	0.537 ^ab^	0.566 ^a^	0.494 ^b^	0.579 ^a^	0.539 ^ab^	0.519 ^ab^	0.552 ^ab^
BE37	2,4,6-Trimethylbenzoic acid	MS	2714	–	1.961	0.035 ^ab^	0.036 ^ab^	0.027 ^b^	0.031 ^b^	0.047 ^a^	0.033 ^b^	0.036 ^ab^
BE38	1H-indole	MS, LRI	2455	2454	1.886	0.697 ^ab^	0.694 ^ab^	0.529 ^b⁎^	0.529 ^b⁎^	0.739 ^a^	0.596 ^ab^	0.532 ^b⁎^
BE39	2,4’-Dihydroxy-3′-methyl acetophenone	MS	1945	–	1.827	0.110 ^ab^	0.228 ^ab^	0.096 ^b^	0.251 ^a^	0.147 ^ab^	0.085 ^b^	0.131 ^ab^
BE40	1,3,5-Trimethyl-2-vinylbenzene	MS	1548	–	1.694	0.128 ^b^	0.176 ^a⁎^	0.149 ^ab^	0.167 ^ab^	0.147 ^ab^	0.170 ^ab⁎^	0.135 ^ab^
BE41	1,2,3-Trimethylbenzene	MS, LRI	1340	1344	1.447	0.455 ^ab^	0.504 ^ab^	0.474 ^ab^	0.404 ^b^	0.610 ^a^	0.560 ^ab^	0.594
BE42	1,2,3,4-Tetramethylbenzene	MS, LRI	1498	1505	1.424	1.17 ^ab^	1.36 ^a^	1.07 ^b^	1.22 ^ab^	1.32 ^ab^	1.22 ^ab^	1.15 ^ab^
BE43	Ethyl *o*-methylbenzoate	MS, LRI	1747	1751	1.361	0.270 ^ab^	0.271 ^ab^	0.234 ^b^	0.271 ^ab^	0.274 ^ab^	0.247 ^ab^	0.281 ^ab^
BE44	*o*-Ethyltoluene	MS, LRI	1282	1271	1.332	0.326 ^b^	0.502 ^ab^	0.513 ^ab^	0.458 ^ab^	0.679 ^a^	0.378 ^ab^	0.574 ^ab^
BE45	Benzoic acid	S, MS, LRI	2438	2432	1.278	5.13 ^ab^	3.58 ^ab^	4.07 ^ab^	3.36 ^ab^	5.59 ^a^	3.00 ^b^	3.70 ^ab^
BE46	3-Methylbenzoic acid	MS	2538	–	1.237	0.138 ^a^	0.087 ^ab^	0.078 ^ab^	0.079 ^ab^	0.112 ^ab^	0.068 ^b^	0.094 ^ab^
BE47	2′,5’-Dimethylcrotonophenone	MS	1997	–	1.220	0.258	0.277	0.264	0.304	0.262	0.259	0.298
BE48	Ethyl cinnamate	MS, LRI	2140	2146	1.171	0.309 ^ab^	0.242 ^ab^	0.255 ^ab^	0.242 ^ab^	0.288 ^ab^	0.195 ^b^	0.323 ^a^
BE49	Ethyl benzoate	MS, LRI	1677	1680	1.143	7.48 ^a^	7.08 ^ab^	7.08 ^ab^	7.14 ^ab^	7.21 ^ab^	6.20 ^b⁎^	7.37 ^ab^
BE50	*o*-Xylene	MS, LRI	1179	1189	1.084	0.51	0.71	0.49	0.50	0.52	0.61	3.95
BE51	Benzaldehyde	S, MS, LRI	1525	1538	0.960	4.88	4.84	3.86 ^⁎^	4.34	4.71	4.51	4.64
BE52	*p*-Isobutyltoluene	MS	1418	–	0.944	0.418 ^ab^	0.428 ^ab^	0.426 ^ab^	0.378 ^ab^	0.481 ^a^	0.426 ^ab^	0.350 ^b^
BE53	*p*-Xylene	MS, LRI	1136	1149	0.874	10.42	20.97 ^⁎^	19.68	18.07	16.12	16.26	42.11
BE54	2-Methylbenzaldehyde	MS, LRI	1629	1622	0.822	0.613	0.627	0.570	0.650	0.638	0.595	0.627
BE55	3-Methylacetophenone	MS, LRI	1785	1789	0.768	0.231	0.250	0.216	0.239	0.229	0.229	0.243
BE56	4-Propylbenzaldehyde	MS	1835	–	0.611	0.059	0.049	0.043	0.054	0.049	0.047	0.058
BE57	Hexyl salicylate ^‡^	MS, LRI	2186	2206	0.551	1.21	1.21	1.06	1.35	1.03	1.15	1.02
BE58	4-Methylbenzaldehyde	MS, LRI	1656	1657	0.531	0.285	0.335	0.279	0.320	0.252	0.324	0.296
BE59	*sec*-Butylbenzene	MS, LRI	1311	1312	0.525	0.748	0.744	0.879	0.758	0.959	0.697	0.960
	*Volatile phenols*											
VP1	4-Vinylguaiacol	S, MS, LRI	2197	2196	29.081	5.68 ^a^	4.74 ^b^	2.32 ^de⁎^	3.47 ^c⁎^	1.91 ^e⁎^	2.79 ^cd⁎^	3.50 ^c⁎^
VP2	4-Vinylphenol	MS, LRI	2398	2406	19.855	4.15 ^a^	3.79 ^b^	2.35 ^c^	3.69 ^b^	2.06 ^c^	2.57 ^c^	3.46 ^b^
VP3	*o*-Cresol	MS, LRI	2011	2011	2.859	0.095 ^abc^	0.099 ^ab^	0.086 ^c^	0.100 ^ab^	0.097 ^abc^	0.090 ^bc^	0.105 ^a^
VP4	Guaiacol	MS, LRI	1866	1869	2.534	0.105 ^abc^	0.095 ^c^	0.110 ^ab^	0.112 ^ab^	0.109 ^abc^	0.101 ^bc^	0.117 ^a^
VP5	2,3,6-Trimethylphenol	MS, LRI	2004	2028	1.990	0.042 ^b^	0.040 ^b^	0.037 ^b⁎^	0.043 ^ab^	0.056 ^a^	0.040 ^b^	0.048 ^ab^
VP6	Phenol	S, MS, LRI	2011	2012	1.481	3.19 ^ab^	3.18 ^ab^	2.92 ^b^	3.36 ^ab^	3.21 ^ab^	3.00 ^ab^	3.47 ^a^
VP7	*p*-*tert*-Amylphenol	MS	2413	–	0.801	0.077	0.028 ^⁎^	0.070	0.049	0.046	0.066	0.059
VP8	Thymol	MS, LRI	2183	2187	0.741	0.077	0.128	0.100	0.115	0.093	0.126	0.096
VP9	*m-*Xylenol	MS, LRI	2176	2174	0.707	0.312	0.314	0.368	0.349	0.386	0.366	0.454
	*Other compounds*											
OC1	Glutaconic anhydride	MS	2004	**–**	4.305	1.71 ^bc^	1.93 ^ab^	1.43 ^c⁎^	1.96 ^ab^	1.96 ^ab^	1.91 ^ab⁎^	2.26 ^a⁎^

Abbreviations: ID – identification of compounds: S – retention time in accordance with that of a pure standard; SC – retention time in accordance with that of a pure standard and compound quantified using calibration curves prepared from pure standard solutions, other compounds were semi-quantified relative to the structurally similar compounds (GC/MS) or internal standard 2-octanol (GC × GC/TOF-MS), assuming a response factor = 1; MS – mass spectrum in accordance with that from NIST 2.0, Wiley 8, and FFNSC 2 mass spectra electronic databases or literature; LRI – linear retention index in accordance with that from literature. Compounds without an S symbol in the ID column were considered tentatively identified. LRI_exp_ – linear retention index determined experimentally; LRI_lit_ – linear retention index from the literature; SC – *Saccharomyces cerevisiae* (control, monoculture); SC × SPx – *Saccharomyces cerevisiae*×*Saccharomyces paradoxus* hybrid (monoculture); TD + SC – *Torulaspora delbrueckii + S. cerevisiae*; MP + SC – *Metschnikowia pulcherrima + S. cerevisiae*; PK + SC – *Pichia kluyveri + S. cerevisiae*; LT + SC – *Lachancea thermotolerans + S. cerevisiae*; SP + SC – *Schizosaccharomyces pombe + S. cerevisiae* (TD + SC, MP + SC, PK + SC, LT + SC, and SP + SC sequential fermentations were finished by *S. cerevisiae* (SC) inoculated at 2 vol% ethanol). Different superscript lowercase letters in a row represent statistically significant differences among wines produced using different yeasts determined by one-way ANOVA and least significant difference test (LSD) at *p* < 0.05. Asterisks represent statistically significant differences between *SC* and each other wine determined by Student's *t*-test at *p* < 0.05.

#### 3.2.1 Hydrocarbons

Azulene had the highest *F*-value and was more abundant in SC × SPx and MP + SC wines than in SC wine, while TD + SC and LT + SC wines had the lowest concentrations. *Trans*,*cis*-2,4-dodecadiene levels were higher in SC, PK + SC, and SP + SC wines than in SC × SPx and LT + SC wines. SC × SPx wine showed a tendency toward a higher concentration of *trans*-1-ethyl-2-methyl-cyclohexane, whereas MP + SC wine showed the same tendency for 3-methylene-4-vinylcyclohex-1-ene (Table 2).

#### 3.2.2 Terpenoids

Terpenes and terpenoids are synthesized and stored in grape berries as glycosides or in free form. Their concentrations in grapes and wine are highly dependent on the cultivar, pedoclimatic conditions, grape cultivation and winemaking techniques, including the yeast strain used in fermentation. The inoculated yeasts significantly altered terpenoid profiles, though the resulting patterns varied (Table 2). TD + SC wine contained more geranyl acetate and 3-carene than the other wines, SC × SPx wine had the highest α-bisabolene concentration, while LT + SC wine had the most β-bisabolene and SP + SC wine the most geraniol. The control SC wine had the highest levels of citronellol, *trans*-2-pinanol, and epoxyterpinolene. SC × SPx wine had the lowest concentrations of *trans*-2-pinanol and epoxyterpinolene, and SP + SC wine had the least β-pinene. Several other terpenoids were also affected by specific treatments compared to the control SC wine. Linalool, the most important wine terpenoid in white wines, due to its pleasant aroma, relatively high concentration, and low perception threshold, was found at higher levels in TD + SC than in SC × SPx and PK + SC wines. Azzolini et al. (2012) and Cheng et al. (2025) reported increased linalool levels with *T. delbrueckii* fermentation compared to *S. cerevisiae* monoculture, while other studies found no significant differences between non-*Saccharomyces* and *S. cerevisiae* fermentations (Benito et al., 2015; Dutraive et al., 2019). Among other major monoterpenols, SC and SP + SC wines had higher nerol levels than SC × SPx wine, and MP + SC wine was richer in ho-trienol and α-terpineol than TD + SC wine (Table 2). The differences observed did not completely match those reported in previous studies. For example, particular strains from *Pichia*, *Metschnikowia*, and *Lachancea* were previously noted for high β-glucosidase activity and thus for a greater potential to release free volatile terpenoids (Han et al., 2021; Zhang et al., 2021). In this study, although β-glucosidase activity was not directly measured, changes in the concentrations of certain free terpenoids may be consistent with the occurrence of such activity, albeit limited to specific compounds. This likely reflected the strong influence of experimental conditions, particularly grape juice composition, on yeast behavior. Moreover, the inconsistent patterns observed in this study (Table 2) suggest that the enzymes responsible for cleaving β-glycosidic bonds and transforming terpenoids might have acted in a compound-specific and highly yeast-dependent manner.

#### 3.2.3 C_13_-norisoprenoids

C_13_-norisoprenoids accumulate in grapes during ripening and are mainly bound at the start of fermentation, with only a small portion present in free form (Tufariello et al., 2021). They originate from carotenoid degradation (e.g., β-carotene, neoxanthin) or exist as glycoconjugates that release volatile aglycones through enzymatic or acid hydrolysis during fermentation. In this study, their distribution varied by treatment without a clear pattern (Table 2). An unidentified norisoprenoid (RI 2212), with the highest *F*-value among norisoprenoids, was found in the highest concentration in TD + SC wine. SC × SPx wine had lower β-cyclocitral levels than the other wines. SP + SC wine contained more *trans*-β-damascenone than TD + SC wine. *Trans*-β-damascenone is a potent wine odorant associated with apple stew and honey notes (Mendes-Pinto, 2009), which may account for subtle sensory differences between the two wines. MP + SC wine had more *cis*-β-damascenone than TD + SC wine. Compared to the SC control wine, vitispirane I, α-ionene, and theaspirane isomer I increased in SC × SPx wine, vitispirane I also rose in concentration in LT + SC and SP + SC wines, while safranal level was higher in MP + SC wine. Among possible reasons, it was assumed that these differences may have stemmed from differing β-glucosidase activity among yeasts (Padilla et al., 2016). No data were found in the literature on yeast interactions with grape carotenoid cleavage oxygenases, the enzymes responsible for norisoprenoid formation from carotenoids.

#### 3.2.4 Volatile thiols

Volatile thiols are almost entirely absent in grapes and are formed during fermentation via yeast β-lyases, which convert odorless S-cysteinylated and glutathionylated precursors. 3-Mercaptohexan-1-ol (3MH) was found in higher concentrations in SC × SPx, TD + SC, MP + SC, PK + SC, and SP + SC wines than in SC and LT + SC wines (Table 2). *T. delbrueckii* strains have been previously shown to produce high levels of thiols, particularly 3MH (Renault et al., 2016; Zott et al., 2011). TD + SC wine, as reported in Table 2, had the highest level of the 3MH acetic acid ester, 3-mercaptohexyl acetate (3MHA), aligning with generally higher acetate levels and the known upregulation of genes involved in acetate synthesis in *T. delbrueckii* (Dutraive et al., 2019, Verstrepen et al., 2003) Given the low odor threshold and fruity character of 3MHA, its higher concentration might have been reflected in an enhanced perception of tropical and fruity aromas in TD + SC wine. Relatively high levels of 3MHA were found in MP + SC, LT + SC, and SP + SC wines (Table 2). Conversely, SC and especially PK + SC wines had the lowest 3MHA concentrations, which differs from the findings of Anfang et al. (2009), who reported enhanced 3MHA production in *P. kluyveri* co-fermentations versus *S. cerevisiae* monocultures.

#### 3.2.5 Aldehydes and ketones

Acetaldehyde, the most abundant and sensory-relevant wine aldehyde, is produced by yeast during alcoholic fermentation as a direct ethanol precursor. The highest concentration was observed in the control SC wine, followed by SP + SC, MP + SC, LT + SC, and PK + SC wines, while the lowest levels were found in SC × SPx and TD + SC wines (Table 2). Several studies have shown that co-fermentation with non-*Saccharomyces* yeasts can lower acetaldehyde levels compared to *S. cerevisiae* (Belda, Ruiz, Esteban-Fernández, et al., 2017; Benito et al., 2015; Ogawa et al., 2022; Ruiz et al., 2018). Acetaldehyde levels generally mirrored those of acetic acid, one of its metabolic products (volatile acidity, Table 1). Among other aldehydes, the level of 2-nonenal was highest in SP + SC wine, and both SC × SPx and SP + SC wines had an elevated content of 2-(acetoxy)-propenal (Table 2). Other aldehydes showed less variation. Among ketones with the highest *F*-values, 1,2-dihydroxycyclobutene-3,4-dione was most abundant in TD + SC wine and least abundant in SC and especially SC × SPx wine. SC and SC × SPx wines tended to have higher levels of 2-undecanone, 2-nonanone, and 3-(acetoxy)-4-methyl-2-pentanone. The concentration of acetoin was higher in SC × SPx wine compared to SC wine.

#### 3.2.6 Major fermentation alcohols

SC × SPx wine showed the highest concentration of 2-phenylethanol, while the other treatment with a *Saccharomyces* yeast, the control SC wine, also contained a relatively high concentration (Table 2). 2-Phenylethanol is a rose-scented alcohol typically present in wine above its odor threshold, implying a greater sensory impact in the wines produced with *Saccharomyces* yeasts. The relatively low levels found in PK + SC, LT + SC, and SP + SC wines corroborated previous studies reporting higher 2-phenylethanol production in *S. cerevisiae* fermentations compared with those involving *T. delbrueckii*, *L. thermotolerans* or *S. pombe* (Hranilović et al., 2021; Loira et al., 2014; Vicente et al., 2021). Major fermentation alcohols (1-propanol, isobutanol, isoamyl alcohol) can negatively impact wine above 300 mg/L with their medicinal or solvent-like aromas. Produced via carbohydrate metabolism and the Ehrlich pathway, their levels are highly yeast-dependent (Tufariello et al., 2021). In this study, as reported in Table 2, Table 1-propanol levels were the highest in TD + SC wine, followed by SC wine. Intermediate levels were observed in SC × SPx, LT + SC, and SP + SC wines, while the lowest concentrations were found in PK + SC and especially MP + SC wine. Previous results on 1-propanol vary, though *S. cerevisiae* generally showed higher production than *S. paradoxus* and *S. pombe* (Benito, 2018; Majdak et al., 2002). *T. delbrueckii* was shown to increase 1-propanol levels relative to *S. cerevisiae* (Loira et al., 2014; Sánchez-Suárez & Peinado, 2024). PK + SC and LT + SC treatments showed a tendency to produce more isobutanol, while the lowest levels were found in SC × SPx and especially SC wine (Table 2). Mixed fermentations with *L.* *thermotolerans* and *S. pombe* have previously been shown to increase isobutanol levels by up to 50% (Del Fresno et al., 2017), and similar effects were observed for *T. delbrueckii* and *L.* *thermotolerans* in sequential fermentation (Zhang et al., 2022). SC × SPx wine contained the highest, and LT + SC and SP + SC wines the lowest concentrations of isoamyl alcohol (Table 2). The observed decrease relative to *S. cerevisiae* was consistent with the findings of Benito (2018) for *S. pombe* and Majdak et al. (2002) for *S. paradoxus*. It was previously shown that some non-*Saccharomyces* yeasts show distinct expression patterns of genes involved in higher alcohol synthesis compared to *S. cerevisiae*. For example, in a study by Tondini et al. (2019), it was found that higher alcohol formation, which depends on branched-chain amino acid catabolism through the Ehrlich pathway, was supported by the expression of key pathway genes in *S. cerevisiae*. In contrast, the lack of detectable transcripts for the branched-chain amino acid permease *BAP2* and the transaminase *BAT2* in *T. delbrueckii* suggested limited amino acid uptake and a lower contribution of this pathway in that species. However, in mixed fermentations the final levels of higher alcohols and other volatile compounds reflect interactions between non-*Saccharomyces* yeasts and *S. cerevisiae* during the course of fermentation, and therefore cannot be explained solely by gene expression patterns observed in one species. Regarding the possible sensory impact and differences between wines, no clear conclusions could be drawn, since each yeast differed in its profile of major higher alcohols rather than showing a consistent increase or decrease across compounds.

#### 3.2.7 Other alcohols

Unlike fermentation alcohols, C_6_-alcohols derive from grape lipids via enzymatic reactions in the lipoxygenase pathway, mainly during harvest and pre-fermentation steps. Their impact on aroma is generally minor due to high perception thresholds, but it can be negative. Although the differences in C_6_-alcohol levels observed in this study were diverse depending on the compound, MP + SC wine generally showed higher concentrations, whereas TD + SC wine showed lower levels (Table 2). Wines from *P. kluyveri* and *M. pulcherrima* starters have previously shown lower 1-hexanol levels than those fermented with *S. cerevisiae* (Hranilović et al., 2020; Vicente et al., 2021). In contrast, *L. thermotolerans* and *T. delbrueckii* tended to increase its concentration (Dutraive et al., 2019; Sadoudi et al., 2012; Sánchez-Suárez et al., 2025; Zhang et al., 2022), as did *T. delbrueckii* and *M. pulcherrima* for *trans*-3-hexen-1-ol (Sadoudi et al., 2012).

Among other minor alcohols, 3-methylpentanol showed the largest *F*-value and proved to be a good discriminator of wines produced by *Saccharomyces* yeasts: SC × SPx wine had the highest concentration, followed by the control SC wine (Table 2). Each wine also showed distinct alcohol markers: 2,7-dimethyl-4,5-octanediol was the most abundant in SC × SPx wine, as was 1-octanol in MP + SC wine, while the concentrations of 2-undecanol, 2-nonanol, and methanol were the highest in the control SC wine (Table 2). Conversely, 1-nonanol, 1-propanol, and 1-octanol levels were the lowest in SC × SPx, MP + SC, and TD + SC wines, respectively. Compared to the SC control, SC × SPx, MP + SC, PK + SC, LT + SC, and SP + SC treatments produced more 1-heptanol, *cis*-6-nonen-1-ol, and *cis*-3-octen-1-ol, while MP + SC, LT + SC, and SP + SC wines had higher *trans*-2-octen-1-ol levels.

#### 3.2.8 Short and branched-chain volatile acids

The odors of pure volatile fatty acids are commonly described as vinegary (acetic acid) and fatty, cheesy, or rancid (short- and medium-chain fatty acids); however, at concentrations typically found in sound, defect-free wines, these compounds are considered to contribute to aroma complexity. The highest *F*-values among acids differentiating treatments were observed for three branched short-chain fatty acids (Table 2). SC × SPx wine, followed by SC wine, had the most 2-methylbutyric acid, while the lowest levels were found in LT + SC and especially SP + SC wines. SC and SC × SPx wines, both fermented with *Saccharomyces* monocultures, were the richest in isovaleric and isohexanoic acids, whereas isovaleric acid level was the lowest in SP + SC and especially TD + SC wines. TD + SC wine had the highest isobutyric acid concentration, while SC and SP + SC wines had the lowest. Branched short-chain fatty acids are formed via amino acid degradation through the Ehrlich pathway (Waterhouse et al., 2016), suggesting distinct amino acid metabolism across the investigated yeasts. Another acid characterized by a high *F*-value was *trans*-2-hexenoic acid (from the lipoxygenase pathway), found at the highest level in SC and PK + SC wines (Table 2). Acetic acid levels, consistent with volatile acidity (Table 1), tended to be lower in SC × SPx and TD + SC wines, aligning with earlier findings on *T. delbrueckii* (Bely et al., 2008; Granchi et al., 2025; Sgouros et al., 2023). Tondini et al. (2019) reported substantially lower levels of acetic acid produced by *T. delbrueckii* compared to *S. cerevisiae*, which they associated with differences in the expression of genes involved in acetate metabolism. Transcripts of aldehyde dehydrogenases *ALD2*–*ALD6*, key enzymes in acetic acid formation, were detected in *S. cerevisiae*, whereas *T. delbrueckii* lacked expression of *ALD3*. In contrast, *T. delbrueckii* consistently expressed alcohol dehydrogenase (*ADH*) genes at higher levels than *ALD* genes. PK + SC, which was among the wines with the highest acetic acid concentrations, did not fully align with trends reported in previous studies, where no change or even a decrease in acetic acid levels was observed following *P. kluyveri* co-fermentation compared to *S. cerevisiae* (Anfang et al., 2009; Benito et al., 2015; Dutraive et al., 2019; Vicente et al., 2021; Ruiz-de-Villa et al., 2023).

#### 3.2.9 Linear medium-chain fatty acids

For linear medium-chain fatty acids, formed from acetyl-CoA via the fatty acid synthase (FAS) complex, the control SC treatment showed stronger production of butyric, hexanoic, and especially octanoic acid, though certain differences were not statistically significant (Table 2). MP + SC and LT + SC wines also had relatively high octanoic acid levels, and PK + SC wine showed a tendency toward higher hexanoic acid concentration (Table 2). TD + SC wine, along with several other wines, showed lower production of hexanoic and octanoic acids, confirming earlier reports on *T. delbrueckii* (Delač Salopek et al., 2022; Silva-Sousa et al., 2024; Zhang et al., 2022). When interpreted in light of the transcriptional analysis of Tondini et al. (2019), the higher medium-chain fatty acid concentrations observed in SC compared to TD + SC wine may be associated with prolonged expression of acetyl-CoA carboxylase (*ACC1*) and the fatty acid synthase (FAS) complex (*FAS1/FAS2*) in *S. cerevisiae* compared with the more transient expression of these genes in *T. delbrueckii*. Other minor acids, including pivalic, undecanoic, propanoic, and isohexanoic acids, were also significantly affected by the treatments (Table 2).

#### 3.2.10 Ethyl esters

Esters are key contributors to wine aroma, particularly fruity notes, and are in the largest part synthesized by yeast during fermentation. Similar to their corresponding short-chain fatty acids, several short-chain ethyl esters had the highest *F*-values among treatments (Table 2). TD + SC wine exhibited the most distinctive ethyl ester profile. It had the highest levels of important aroma compounds such as ethyl propanoate, ethyl isobutyrate, and ethyl butyrate known for their fruity aroma reminiscent of pineapple and strawberry, along with several minor esters of uncertain sensory impact, including ethyl 2-butenoate, 4-hexenoate I, 3-hydroxybutyrate, *trans*-2-butenoate, methyl succinate, 3-hexenoate, and pentanoate. The elevated production of short-chain ethyl esters observed may arise from species- and strain-specific differences connected with precursor availability (fatty acids sythesis metabolism) and the expression of genes encoding enzymes responsible for ethyl ester formation, such as *EEB1* and *EHT1* (Saerens et al., 2006). Tondini et al. (2019) reported a species-dependent expression pattern, with *T. delbrueckii* favoring *EHT1* expression and *S. cerevisiae* exhibiting stronger expression of *EEB1*. Previous studies identified *T. delbrueckii* as a good producer of ethyl propanoate, isobutyrate, and butyrate (Renault et al., 2015; Zhang et al., 2022), while Renault et al. (2015) considered ethyl pentanoate and isobutyrate as markers of its activity. Conversely, as reported in Table 2, TD + SC wine showed lower concentrations of several unsaturated and saturated medium- and long-chain ethyl esters, likely due to reduced precursor availability and/or enzymatic activity. *Saccharomyces*-fermented wines (SC, SC × SPx) had higher levels of ethyl 3-methylbutyrate and lower levels of 2-butenoates, while SP + SC wine had lower concentrations of ethyl propanoate, 3-methylbutyrate, isobutyrate, and 2-methylbutyrate, mirroring trends in their fatty acid analogues. Medium-chain saturated esters like ethyl hexanoate, octanoate, and decanoate, which are formed via the fatty acid synthase (FAS) complex, are crucial to fruity wine aromas and are believed to depend more on precursor availability (acetyl-CoA and elongation intermediates) than on enzyme activity (Saerens et al., 2010). The accumulation of medium-chain esters during fermentation is hypothesized to be correlated with depletion of unsaturated fatty acids and sterols, and with the arrest of fatty acid biosynthesis resulting in the accumulation of long-chain saturated acyl-CoA compounds, which inhibits the initial stages of fatty acid synthesis. Under these conditions, medium-chain fatty acids are released from the FAS complex in free form and/or as ethyl esters, which are subsequently excreted from the yeast cell (Waterhouse et al., 2016). Ethyl octanoate concentration peaked in LT + SC wine, with no significant difference from MP + SC, PK + SC, and SP + SC wines (Table 2). Benito et al. (2015), Ruiz et al. (2018), and Sánchez-Suárez et al. (2025) observed an increase in ethyl octanoate concentration in wine produced by sequential inoculation with *M. pulcherrima* compared to a *S. cerevisiae* control. Ethyl hexanoate was most abundant in SP + SC wine, followed by LT + SC and SC wines (Table 2). Ethyl decanoate concentration was higher in MP + SC wine than in SC × SPx, TD + SC, and especially SC wine. In terms of sensory relevance, ethyl ester concentrations varied in a yeast- and compound-dependent manner, preventing the identification of consistent trends among wines. Nevertheless, given the magnitude and direction of the compositional differences observed, these variations were likely to result in subtle differences in fruity notes between the wines.

Distinct patterns also emerged among some minor ethyl esters. PK + SC wine had higher levels of ethyl 2-hexenoate II and ethyl pentadecanoate. Succinic acid-derived esters, such as ethyl isoamyl succinate, ethyl butyl succinate, ethyl propyl succinate, and ethyl methyl succinate, were found in higher concentrations in SC × SPx than in SC wine. The production of long-chain fatty acid ethyl esters was higher in wines from *M. pulcherrima* and *P. kluyveri* starters: MP + SC and PK + SC wines contained more ethyl tetradecanoate and dodecanoate, MP + SC wine had more ethyl 9-decenoate, and PK + SC wine more ethyl undecanoate and tridecanoate compared to SC wine. LT + SC wine contained more ethyl octadecanoate, while SP + SC wine had more ethyl *cis*-4-octenoate and 7-octenoate than SC wine (Table 2).

#### 3.2.11 Acetate esters

Acetate esters, produced from higher alcohols and acetyl-CoA via yeast alcohol acetyltransferases, strongly contribute fruity and floral aromas (Waterhouse et al., 2016). TD + SC wine stood out with generally the highest concentrations, including less known acetates such as 3-ethoxypropyl, 1,3-butanediol, thujyl, 2-ethylbutyl, 3-methyl-1,4-pentadien-3-yl, and *trans*-penten-1-yl acetate, standard fermentation products such as isopropyl, *cis*-3-hexen-1-yl, pentyl, and butyl acetate, as well as the most important acetates for the sensory quality of wine, such as ethyl, isoamyl, isobutyl, and hexyl acetate (Table 2). Previous studies reported increased production of isoamyl and isobutyl acetates in *T. delbrueckii* co-fermentations (Renault et al., 2015; Sanoppa et al., 2019), whereas Azzolini et al. (2015) reported conflicting results. Acetate increases in TD + SC wine (Table 2) coincided with reduced acetic acid (Table 1), while a strong inverse correlation between total acetates and acetaldehyde concentrations was found across the whole dataset (*r* = −0.695, *n* = 21). This pointed to the *T. delbrueckii*-specific, but also general competition between the expression of: (i) *ATF* genes promoting acetate synthesis via acetyl-CoA and (ii) *PDC/ALD* genes leading to acetaldehyde and acetic acid from pyruvate (Dzialo et al., 2017). SP + SC wine followed TD + SC wine with high acetate concentrations, including 1,2,4-butanetriol, *cis*-6-nonen-1-yl, and heptyl acetate (Table 2). Alongside TD + SC, SC × SPx wine had the highest concentration of 2-phenethyl acetate. In TD + SC fermentation, this was likely driven by strong *ATF* gene expression (Saerens et al., 2010), while in SC × SPx it probably reflected higher availability of the precursor 2-phenylethanol (Table 2), as other acetate levels in this wine were generally low. No consistent correlations were observed between other acetates and their corresponding alcohol precursors, supporting the hypothesis that *ATF1*/*ATF2* gene expression plays a stronger role than precursor availability (Saerens et al., 2010). *Saccharomyces*-fermented wines, SC and SC × SPx, shared certain traits: higher levels of an unidentified diol acetate (RI 1741) and lower levels of various esters, including *cis*−/*trans*-3-hexen-1-yl, butyl, pentyl, methyl, and isoamyl acetates compared to the other wines (Table 2). Beyond TD + SC wine, LT + SC and SP + SC wines had higher ethyl acetate levels, while SP + SC wine showed a higher concentration of isoamyl acetate than the other wines. The lowest isoamyl acetate level was found in SC × SPx and especially SC wine. PK + SC wine had the highest concentration of *trans*-3-hexen-1-yl acetate, while LT + SC wine showed a tendency toward lower concentrations of *trans*-3-hexen-1-yl, 2-phenethyl, and hexyl acetates.

Major acetate esters are well known to contribute to the fruity character of wine, each imparting distinct sensory notes, for example isoamyl acetate with banana aroma, hexyl acetate with pear-like notes, and ethyl and isobutyl acetate with general fruity aromas (Belda, Ruiz, Esteban-Fernández, et al., 2017). The higher levels of key acetate esters in TD + SC wine may therefore indicate a more pronounced fruity aroma and enhanced overall sensory quality, whereas the lower isoamyl acetate concentrations observed in SC × SPx and SC wines could be associated with a less intense fruity, particularly banana-like, note.

#### 3.2.12 Other esters

Among other esters, consistent patterns were also evident. SC × SPx wine had the highest concentrations of several 2-phenylethanol esters, with 2-phenylethyl formate, isobutyrate, isovalerate, and hexanoate displaying high *F*-values (Table 2). Although sensory perception thresholds for these esters in wine have not yet been established, their potentially enhanced contribution to the fruity and floral aroma of SC × SPx wine cannot be excluded. This wine also showed elevated levels of esters of isoamyl alcohol and isobutanol with short- and medium-chain fatty acids, which are also associated with fruity notes, though not always with statistical significance (Table 2). MP + SC wine had higher levels of diethyl esters of dicarboxylic acids, such as malate, glutarate, fumarate and malonate, sometimes matched by PK + SC wine. The lowest level of diethyl malate was found in SP + SC wine, clearly linked to the reduced concentration of its precursor, malic acid (Table 1). The same wine exhibited increased levels of esters of octanoic and decanoic acids. Esters formed from methanol and higher alcohols with medium-chain FAS acids were generally more abundant in MP + SC, PK + SC, SP + SC, and in some cases LT + SC wines, than in other wines. TD + SC wine showed relatively low levels of methyl and isoamyl alcohol esters, while the concentrations of isobutyl esters were the lowest in SC wine. Several lesser-known esters also varied significantly across non-*Saccharomyces* wines compared to the SC control wine.

#### 3.2.13 Benzenoids

Some benzenoids originate from grape phenylpropanoid pathways, while others form during fermentation from aromatic amino acids like phenylalanine and tyrosine. Another possible source of benzenoids in wine may be contamination associated with environmental exposure or migration from contact materials used during winemaking, storage, or bottling, as documented for petroleum-derived aromatic hydrocarbons in wine (Baldock & Hayasaka, 2004). SC × SPx wine contained elevated levels of several benzenoids from phenylalanine metabolism: benzeneacetaldehyde, benzeneacetic acid, ethyl benzeneacetate, ethyl phenethyl ether, and acetaldehyde ethyl phenethyl acetal (Table 2). Given its high content of 2-phenylethanol from the same pathway, this suggests that the *S. cerevisiae×S. paradoxus* strain used may exhibit increased expression of genes involved in phenylalanine transamination and phenylpyruvate decarboxylation in the Ehrlich pathway (Dzialo et al., 2017). Similarly, the control SC wine, followed by MP + SC wine, as reported in Table 2, showed increased levels of these compounds compared to some other treatments, reflecting the pattern observed for 2-phenylethanol. SC × SPx wine also had the highest levels of other benzenoids including 2-(2-phenylethoxy)propanal, cardene, 3,3-dimethoxy-1-phenylpropane-1,2-dione, styrene, and one unidentified compound (RI 2187). Numerous other benzenoids were identified with specific, though less pronounced, differences across wines. Azzolini et al. (2015) reported increased benzeneacetaldehyde levels in *T. delbrueckii* co-fermentation compared with *S. cerevisiae* monoculture fermentation.

#### 3.2.14 Furanoids and lactones

2-Butyltetrahydrofuran had the highest concentration in SC × SPx wine, followed by SC, while TD + SC wine contained the lowest concentration (Table 2). γ-Butyrolactone had a high *F*-ratio and its concentration was the highest in SC wine. This finding differed from a previous study, which observed elevated levels of γ-butyrolactone after both sequential and co-inoculation with *T. delbrueckii* relative to *S. cerevisiae* monoculture fermentation. (Azzolini et al., 2015)*.* LT + SC wine contained the most γ-decalactone and β-methyl-γ-butyrolactone, MP + SC wine had the highest γ-nonalactone concentration, and SC × SPx wine had the most pantolactone (Table 2). These differences likely resulted from varying availability of hydroxycarboxylic acid precursors or from yeast-specific enzymatic activity.

#### 3.2.15 Sulfur-containing compounds

Non-thiol sulfur compounds mainly form via yeast metabolism of sulfur-containing amino acids cysteine and methionine during fermentation (Dzialo et al., 2017). 2-Hydroxyethyl 3-hydroxypropyl sulfides I and II had the highest *F*-values, with maximum concentrations in TD + SC and minimum concentrations in SC wine (Table 2). TD + SC wine also had the highest levels of mercapto-2-propanone, methionyl acetate, and dihydro-2-methyl-3(2H)-thiophenone. SC × SPx wine was the richest in 2-thiophenecarboxaldehyde and methionol, while SP + SC wine had the highest S-ethyl octanethioate concentration. Methionol levels were also high in MP + SC wine, consistent with the highest concentration of its product methional in SC × SPx and MP + SC wines.

#### 3.2.16 Volatile phenols

4-Vinylguaiacol concentration showed the most variation, being the highest in the SC control wine, followed by SC × SPx wine, and the lowest in PK + SC wine (Table 2). 4-Vinylphenol was also the most abundant in SC wine, followed by SC × SPx, MP + SC and SP + SC wines. Vinylphenols are produced via yeast decarboxylation of ferulic and *p*-coumaric acid by hydroxycinnamic acid decarboxylases (Waterhouse et al., 2016). Non-*Saccharomyces* yeasts typically show lower vinylphenol production than *S. cerevisiae* (Binati et al., 2020). The results of this study align with López-Enríquez et al. (2022), who found strong decarboxylase activity in most *S. cerevisiae* strains, and Escribano-Viana et al. (2017), who reported such activity only in some *M. pulcherrima* strains, and none in particular *T. delbrueckii*, *P. kluyveri*, and *L.* *thermotolerans* strains. Non-*Saccharomyces* yeasts may therefore represent an effective strategy for lowering the concentration of volatile phenols, which have very low sensory perception thresholds and are commonly associated with undesirable sensory attributes in wine, ranging from sweaty saddle odors to clove-like notes (Chatonnet et al., 1992; Morata et al., 2021).

### 3.3 Phenolic compounds

The effects of the treatments on grape-derived phenolic compounds are shown in Table 3. Among hydroxybenzoic acids, LT + SC wine had higher levels of *p*-hydroxybenzoic and protocatechuic acids than the control SC wine. MP + SC and PK + SC wines tended toward higher concentrations of 2,3-dihydroxybenzoic acid. Free hydroxycinnamic acids showed more pronounced variation, with lower levels of *p*-coumaric and ferulic acids in SC × SPx wine and especially SC wine. The observed difference may be explained by the higher decarboxylase activity of the two *Saccharomyces* yeasts, as reported in previous studies (Escribano-Viana et al., 2017; López-Enríquez et al., 2022), especially considering that this pattern was consistent with the elevated levels of their decarboxylation products, volatile phenols 4-vinylphenol and 4-vinylguaiacol, in these wines (Table 2). LT + SC wine had the lowest caffeic acid concentration (Table 3). SC × SPx wine showed higher levels of *trans*-caftaric and *trans*-fertaric acids, the main hydroxycinnamoyltartrates, compared with most other wines. The highest 4-aminobenzoic acid level was found in TD + SC wine, followed by SC × SPx wine, with the lowest level observed in MP + SC wine. Flavan-3-ols and flavonols varied less across treatments. Total phenolic content was reduced in TD + SC and PK + SC wines compared to the control SC wine. In addition to enzymatic effects, the observed variations might also have resulted from differential yeast adsorption, influenced by cell wall surface area, diffusion capacity, and phenol polarity (Zhang et al., 2021).

**Table 3:** Concentrations of phenolic compounds (mg/L) determined by ultra-performance liquid chromatography/mass spectrometry (UPLC/MS/MS) sorted by compound class and descending Fisher *F*-ratio and concentration of total phenols (mg/L gallic acid equivalents) in Malvazija istarska white wines produced by fermentation with different yeasts.

Phenols	*F*- ratio				Wine			
		SC	SC × SPx	TD + SC	MP + SC	PK + SC	LT + SC	SP + SC
*Hydroxybenzoic acid derivatives*								
2,5-Dihydroxybenzoic acid	3.245	0.278 ^b^	0.323 ^ab^	0.328 ^ab^	0.354 ^a^	0.358 ^a⁎^	0.286 ^b^	0.275 ^b^
*p*-Hydroxybenzoic acid	1.902	0.182 ^b^	0.225 ^ab^	0.245 ^ab^	0.261 ^ab^	0.159 ^b^	0.299 ^a⁎^	0.253 ^ab^
Protocatechuic acid	1.184	0.246 ^b^	0.430 ^ab^	0.450 ^ab^	0.501 ^ab⁎^	0.514 ^ab⁎^	0.606 ^a^	0.409 ^ab^
Syringic acid	1.008	0.405	0.466	0.331	0.298	0.431	0.330	0.317
Vanillic acid	0.874	0.093	0.098	0.087	0.111	0.084	0.092	0.095
*Hydroxycinnamic acid derivatives*								
Ferulic acid	10.062	0.454 ^d^	0.530 ^cd^	0.695 ^ab⁎^	0.697 ^ab⁎^	0.799 ^a⁎^	0.626 ^bc⁎^	0.761 ^a⁎^
*p*-Coumaric acid	7.295	0.377 ^d^	0.437 ^cd^	0.501 ^abc⁎^	0.503 ^abc⁎^	0.534 ^ab⁎^	0.471 ^bc⁎^	0.561 ^a⁎^
*trans*-Caftaric acid	4.804	1.37 ^ab^	1.38 ^a^	1.10 ^d⁎^	1.26 ^abc^	1.22 ^bcd^	1.21 ^cd^	1.09 ^d⁎^
*trans*-Fertaric acid	2.642	2.82 ^b^	3.16 ^a^	2.76 ^b^	2.80 ^b^	2.89 ^ab^	2.74 ^b^	2.73 ^b^
Caffeic acid	2.602	2.25 ^a^	2.22 ^a^	2.36 ^a^	2.28 ^a^	2.22 ^a^	2.00 ^b⁎^	2.29 ^a^
*trans*-Coutaric acid	0.935	0.764	0.837	0.760	0.782	0.781	0.758	0.813
*Other acids*								
4-Aminobenzoic acid	28.203	0.090 ^c^	0.136 ^b⁎^	0.186 ^a⁎^	0.063 ^d⁎^	0.096 ^c^	0.107 ^c^	0.103 ^c^
*Stilbenes*								
*cis*-Resveratrol	4.202	0.042 ^b^	0.067 ^a⁎^	0.062 ^a^	0.044 ^b^	0.054 ^ab^	0.037 ^b^	0.042 ^b^
*trans*-Resveratrol	2.253	0.083 ^b^	0.099 ^a⁎^	0.090 ^ab⁎^	0.091 ^ab⁎^	0.091 ^ab^	0.093 ^a^	0.095 ^a⁎^
*Flavan-3-ols*								
Catechin	3.646	1.49 ^ab^	1.64 ^a^	1.33 ^bc^	1.53 ^a^	1.46 ^abc^	1.51 ^ab^	1.30 ^c^
Gallocatechin	2.423	0.209 ^a^	0.165 ^abc^	0.188 ^ab^	0.172 ^abc^	0.160 ^abc^	0.150 ^bc^	0.120 ^c⁎^
Procyanidin B2 + B4	1.185	0.425 ^ab^	0.442 ^ab^	0.254 ^b^	0.505 ^a^	0.410 ^ab^	0.382 ^ab^	0.389 ^ab^
Procyanidin B1	1.058	2.85	2.93	2.83	3.39	2.95	3.19	3.33^⁎^
Epicatechin	1.030	0.411	0.432	0.419	0.368	0.447	0.402	0.284
Epigallocatechin	0.927	0.013	0.018	0.016	0.014	0.008	0.008	0.015
*Flavonols*								
Quercetin	2.345	0.153 ^abc^	0.179 ^ab^	0.146 ^bc^	0.149 ^bc^	0.159 ^abc^	0.140 ^c^	0.185 ^a^
Kaempferol	2.131	0.008 ^ab^	0.016 ^ab^	0.001 ^b^	0.024 ^a^	0.022 ^a⁎^	0.011 ^ab^	0.026 ^a⁎^
*Miscellaneous*								
Catechol	1.328	0.293	0.431	0.255	0.225	0.313	0.366	0.427^⁎^
Phlorizin	0.802	0.072	0.072	0.085	0.072	0.064	0.060	0.068
*Total phenols*		179.9 ^a^	177.8 ^ab^	166.2 ^bc⁎^	174.0 ^abc^	162.7 ^c^	170.0 ^abc^	180.9 ^a^

Abbreviations: SC – *Saccharomyces cerevisiae* (control, monoculture); SC × SPx – *Saccharomyces cerevisiae*×*Saccharomyces paradoxus* hybrid (monoculture); TD + SC – *Torulaspora delbrueckii + S. cerevisiae*; MP + SC – *Metschnikowia pulcherrima + S. cerevisiae*; PK + SC – *Pichia kluyveri + S. cerevisiae*; *LT + SC* – *Lachancea thermotolerans + S. cerevisiae*; *SP + SC* – *Schizosaccharomyces pombe + S. cerevisiae* (TD + SC, MP + SC, PK + SC, LT + SC, and SP + SC sequential fermentations were finished by *S. cerevisiae* (SC) inoculated at 2 vol% ethanol). Different superscript lowercase letters in a row represent statistically significant differences among wines produced using different yeasts determined by one-way ANOVA and least significant difference test (LSD) at *p* < 0.05. Asterisks represent statistically significant differences between *SC* and each other wine determined by Student's *t*-test at *p* < 0.05.

### 3.4 Multivariate differentiation

**Fig. 1:**
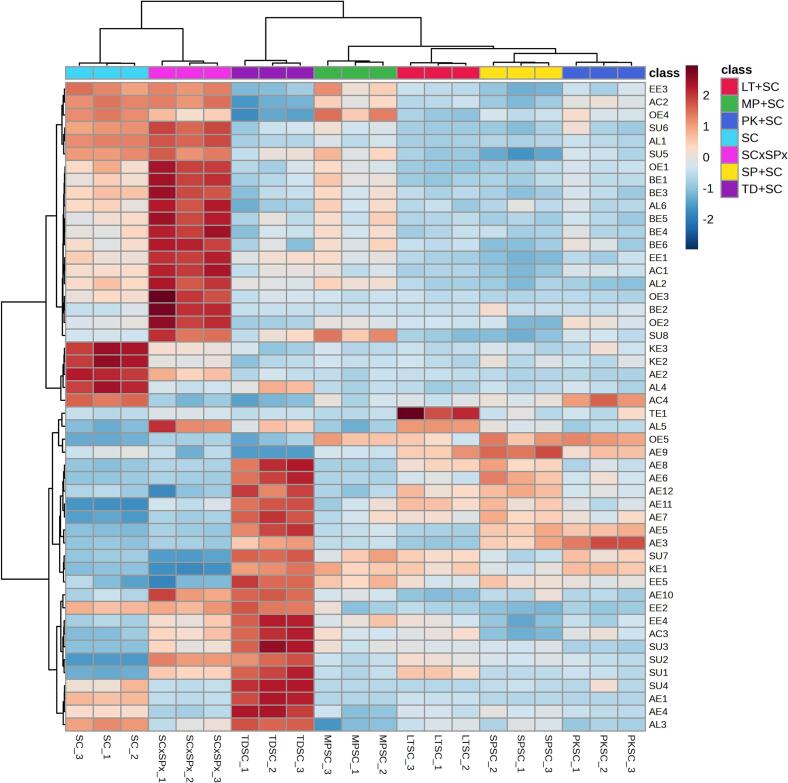
Hierarchical clustering analysis performed using volatile aroma compound profiles of Malvazija istarska wines produced by fermentation with different yeasts determined by GC/FID, GC/MS and GC × GC/TOF-MS analysis. The heatmap was generated using 50 most significant compounds according to their highest *F*-ratios. Compounds are designated by codes which correspond to those in Table 2. *Saccharomyces cerevisiae* (SC; control) and *Saccharomyces cerevisiae*×*Saccharomyces paradoxus* hybrid (SC × SPx) fermented in monoculture, while *Torulaspora delbrueckii* (TD + SC), *Metschnikowia pulcherrima* (MP + SC), *Pichia kluyveri* (PK + SC), *Lachancea thermotolerans* (LT + SC) and *Schizosaccharomyces pombe* (SP + SC) were inoculated as fermentation starters followed by sequential inoculation of *S. cerevisiae* (SC) at 2 vol% ethanol.

**Fig. 2:**
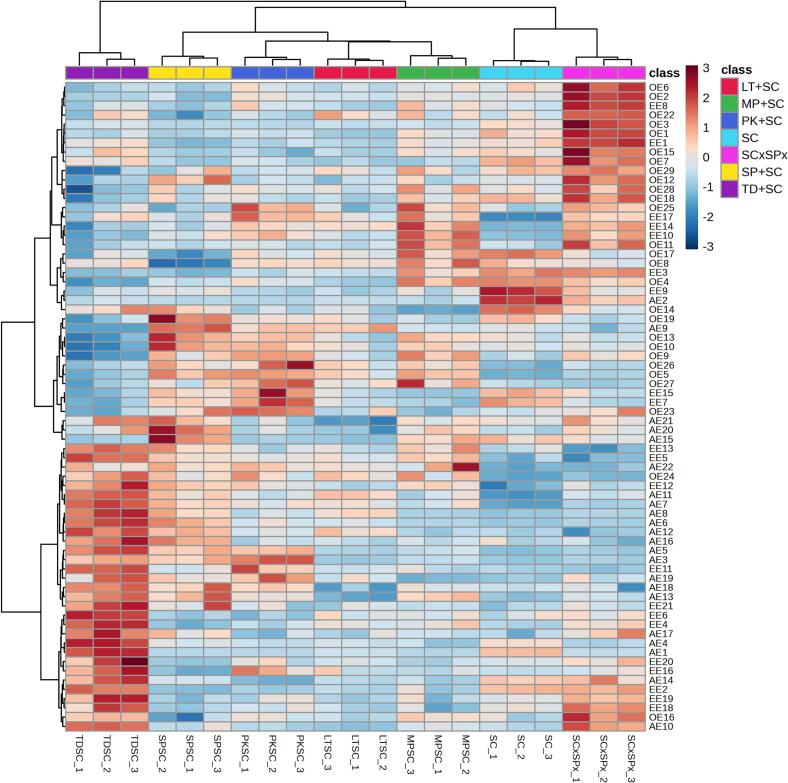
Hierarchical clustering analysis performed using volatile ester profiles of Malvazija istarska wines produced by fermentation with different yeasts determined by GC/FID, GC/MS and GC × GC/TOF-MS analysis. The heatmap was generated using 70 most significant esters according to their highest *F*-ratios. Compounds are designated by codes which correspond to those in Table 2. *Saccharomyces cerevisiae* (SC; control) and *Saccharomyces cerevisiae*×*Saccharomyces paradoxus* hybrid (SC × SPx) fermented in monoculture, while *Torulaspora delbrueckii* (TD + SC), *Metschnikowia pulcherrima* (MP + SC), *Pichia kluyveri* (PK + SC), *Lachancea thermotolerans* (LT + SC) and *Schizosaccharomyces pombe* (SP + SC) were inoculated as fermentation starters followed by sequential inoculation of *S. cerevisiae* (SC) at 2 vol% ethanol.

Hierarchical cluster analysis (HCA) was conducted on two datasets to better visualize relationships between treatments and the volatile profiles of the produced wines (Fig. 1, Fig. 2). Using the 50 volatile compounds with the highest *F*-values in the overall dataset, SC and SC × SPx wines, both resulting from *Saccharomyces* fermentations, clustered together and were clearly separated from non-*Saccharomyces* wines (Fig. 1). This distinction was driven by higher levels of 2-phenylethanol, certain other alcohols, benzenoids, and short-chain acids, and by lower levels of key odorants such as isoamyl, isobutyl, and other acetate esters. TD + SC wines formed their own cluster, mainly due to high acetate concentrations. Each wine was distinguishable by specific patterns of elevated or reduced levels of volatile compounds. The second HCA, based on the 70 esters with the highest *F*-values, again placed TD + SC wines apart due to their high concentrations of acetates and select ethyl esters, and their lower concentrations of others (Fig. 2). SC and SC × SPx wines formed a separate cluster, and the heatmap highlighted unique ester composition profiles for each yeast.

**Fig. 3:**
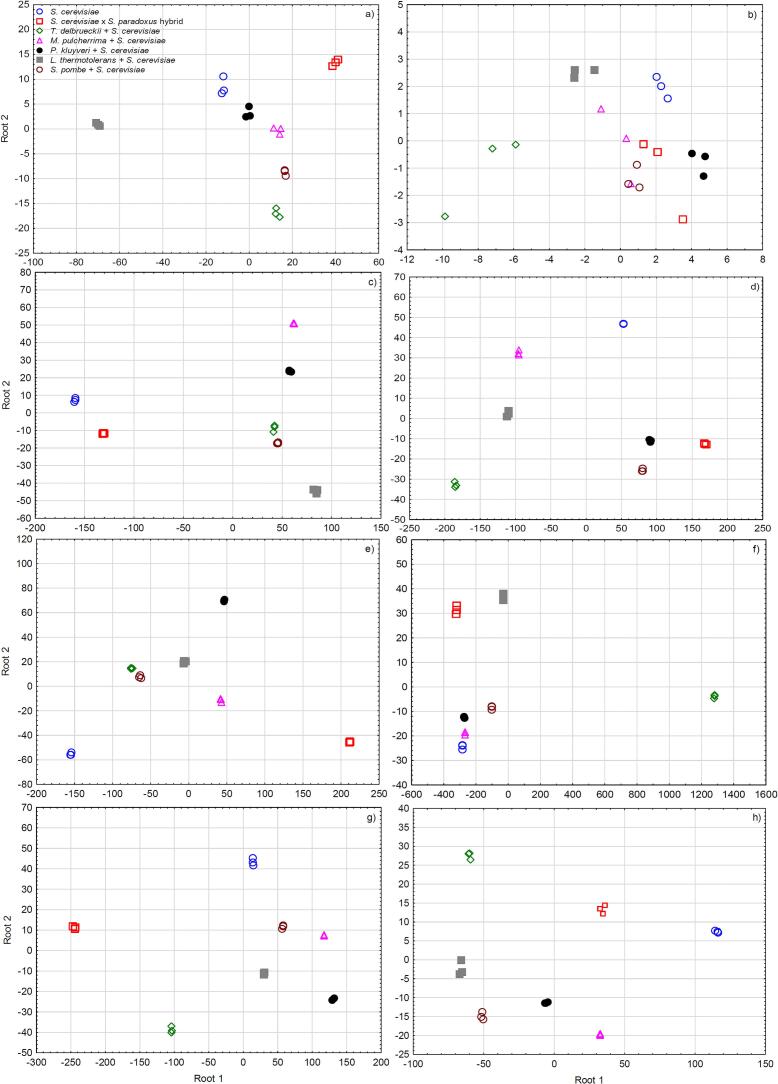
Separation of Malvazija istarska wines according to yeast used in fermentation defined by the first two discriminant functions (roots) obtained by forward stepwise discriminant analysis (SLDA) on the basis of the composition of volatile compounds from different chemical classes determined by GC/FID, GC/MS and GC × GC/TOF-MS analysis: a) terpenoids, b) thiols, c) alcohols, d) acids, e) ethyl esters, f) acetate esters, g) other esters and h) sulfur containing compounds. *Saccharomyces cerevisiae* (SC; control) and *Saccharomyces cerevisiae*×*Saccharomyces paradoxus* hybrid (SC × SPx) fermented in monoculture, while *Torulaspora delbrueckii* (TD + SC), *Metschnikowia pulcherrima* (MP + SC), *Pichia kluyveri* (PK + SC), *Lachancea thermotolerans* (LT + SC) and *Schizosaccharomyces pombe* (SP + SC) were inoculated as fermentation starters followed by sequential inoculation of *S. cerevisiae* (SC) at 2 vol% ethanol.

Stepwise linear discriminant analysis (SLDA), applied to GC data for each compound group separatelly, successfully differentiated wines by yeast species (Fig. 3, Figs. S1–S16). For most compound groups, a few key variables achieved 100% correct classification, for example, 2-methylbutyric and isovaleric acids (acids), acetaldehyde, 2-nonenal, and 2-(acetoxy)-propenal (aldehydes), 3-methylpentanol, 1-propanol, and 2-phenylethanol (alcohols), and ethyl propanoate, ethyl 3-methylbutyrate, and ethyl 2-hexenoate (ethyl esters). Additional compounds further improved classification (Tables S1–S16). Overall, compounds formed during fermentation, such as alcohols, acids, and esters, were the most effective for differentiation. Based on total squared Mahalanobis distances of wine samples from group centroids in the full discriminant space, specific compound classes served as stronger discriminators (positive or negative) of particular wines from the others: terpenoids for LT + SC wine (Table S17), thiols and acetates for TD + SC wine (Tables S18 and S19, respectively), aldehydes for SP + SC wine (Table S20), norisoprenoids, benzenoids, ethyl esters, and other esters for SC × SPx wine (Tables S21, S22, S23, and S24 respectively), and ketones, alcohols, and volatile phenols for *Saccharomyces* monoculture fermentation wines SC and SC × SPx (Tables S25, S26, and S27, respectively).

## 4 Conclusions

This study demonstrated that the yeasts investigated had markedly different effects on the physico-chemical composition of Malvazija istarska white wine. The most significant impact on basic wine parameters was observed with sequential inoculation using *S. pombe*, which notably reduced acidity. A total of 399 volatile compounds from diverse chemical classes were identified using GC/FID, GC/MS, and GC × GC/TOF-MS, providing a detailed characterization of the yeast-driven modulation of white wine volatile composition. Common effects of non-*Saccharomyces* yeasts compared to *S. cerevisiae* included reduced levels of acetaldehyde, 2-phenylethanol, short- and medium-chain fatty acids, and major volatile phenols, alongside increased concentrations of isobutanol, its esters, and isoamyl acetate. For most of these compounds, such changes could be considered positive for the aroma and overall sensory quality of white wine. Wine fermented with *T. delbrueckii* showed the most distinctive ester profile, with elevated short-chain ethyl esters and acetates, including 3-mercaptohexyl acetate, and lower levels of acetaldehyde, medium-chain acids, and their ethyl esters, a combination that may indicate a distinct and potentially more pronounced fruity aroma and enhanced overall sensory quality. Fermentation with the *S. cerevisiae × S. paradoxus* hybrid yielded higher concentrations of phenylalanine-derived benzenoids, including 2-phenylethanol and its esters, along with increased branched-chain fatty acids and volatile phenols, which were features shared by both *Saccharomyces* yeasts studied and which could potentially contribute to a distinct aroma profile. Each yeast also influenced the concentration of various other volatile markers, some previously unidentified or overlooked, supporting the hypothesis that their impact is broader than currently understood. This extended to compounds not directly produced during fermentation but transformed during its course, such as terpenoids, norisoprenoids, thiols, C_6_-alcohols, lactones, and furanoids, categories rarely explored in this context. Notably, the strongest discrimination arose from combinations of variables in multivariate models rather than from single stand-alone markers. The GC data from most chemical classes enabled clear multivariate differentiation of wines by yeast. Given the differences in the composition of aroma compounds, both desirable and undesirable, pronounced differences in sensory characteristics and overall aroma expression were highly likely among the wines. Although subtler, non-*Saccharomyces* yeasts also influenced grape phenolics, particularly through preservation of *p*-coumaric and ferulic acids, likely due to lower decarboxylase activity. Overall, the study offers deeper insight into the biochemical activity and enological potential of the tested yeasts, contributing valuable knowledge to the understanding of yeast-driven wine complexity and quality.

## CRediT authorship contribution statement

**Doris Delač Salopek:** Writing – review & editing, Writing – original draft, Visualization, Validation, Methodology, Investigation, Formal analysis, Data curation, Conceptualization. **Urska Vrhovsek:** Writing – review & editing, Validation, Supervision, Resources, Methodology, Investigation. **Silvia Carlin:** Writing – review & editing, Validation, Software, Investigation, Formal analysis, Data curation. **Sanja Radeka:** Writing – review & editing, Investigation, Formal analysis. **Marina Tomašević:** Writing – review & editing, Validation, Resources, Investigation, Formal analysis. **Igor Lukić:** Writing – review & editing, Validation, Supervision, Resources, Project administration, Methodology, Investigation, Funding acquisition, Data curation, Conceptualization.

## Funding

This study was funded by the Croatian Science Foundation under the projects HRZZ-IP-2020-02-4551 and HRZZ-DOK-2021-02-5500. This research is part of the ongoing doctoral thesis of Doris Delač Salopek at the Faculty of Food Technology and Biotechnology, University of Zagreb, Croatia.

## Declaration of competing interest

The authors declare that they have no known competing financial interests or personal relationships that could have appeared to influence the work reported in this paper.

## Data Availability

Data will be made available on request.

## Appendix A Supplementary data

Supplementary material Image 1
