# Supplementary material for: From detail to diversity: Capturing the chemical signature of non-Saccharomyces yeasts in white wine through GC×GC/TOF-MS metabolomics and complementary analytical approaches
Source: Food Chem X. 2026 Mar 24;35:103789. doi: 10.1016/j.fochx.2026.103789 (PMC13062559; doi:10.1016/j.fochx.2026.103789)
Supplement: Supplementary file 1 — Supplementary material [file mmc1.docx]

**Supplementary Data**


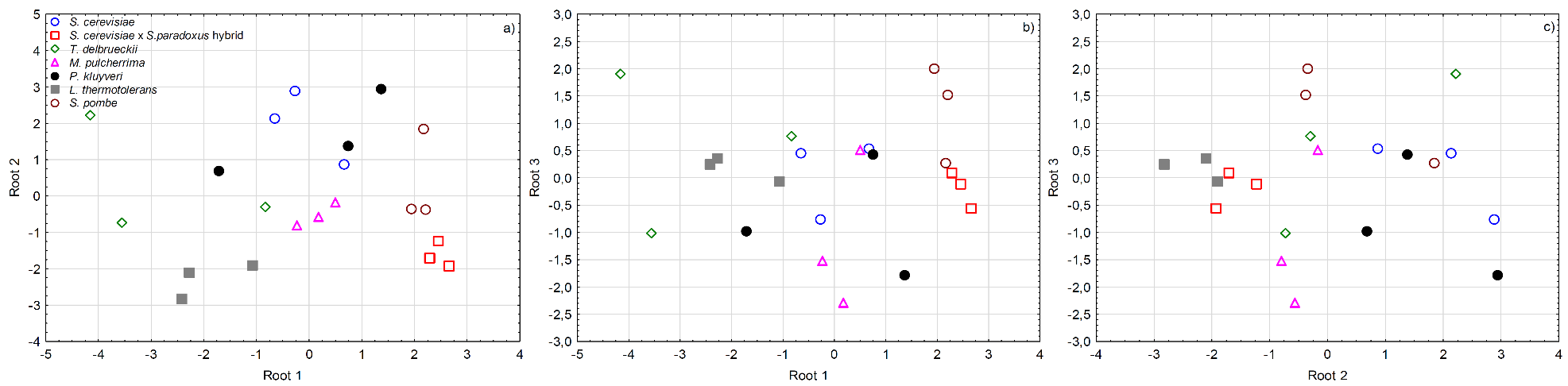


Figure S1. Separation of Malvazija istarska wines according to yeast used in fermentation defined by the first three discriminant functions (roots) obtained by forward stepwise linear discriminant analysis (SLDA) on the basis of the composition of hydrocarbons determined by GC×GC/TOF-MS analysis.

Table S1. Variables from the group of hydrocarbons included in the SLDA model with their contribution to the correct classification (%) of Malvazija istarska wines according to yeast used in fermentation.

| Hydrocarbons | Wine | | | | | | | |
| --- | --- | --- | --- | --- | --- | --- | --- | --- |
|  | SC | SC×SPx | TD+SC | MP+SC | PK+SC | LT+SC | SP+SC | TOT |
| Azulene | 100.0 | 66.7 | 0.0 | 0,0 | 0.0 | 66.7 | 33.3 | 38.1 |
| *trans,cis*-2,4-Dodecadiene | 100.0 | 66.7 | 33.3 | 66,7 | 33.3 | 100.0 | 66.7 | 66.7 |
| 1,3,5,5-Tetramethyl-1,3-cyclohexadiene | 66.7 | 100.0 | 66.7 | 100,0 | 33.3 | 100.0 | 100.0 | 81.0 |
| *trans*-1-Ethyl-2-methyl-cyclohexane | 100.0 | 100.0 | 66.7 | 100,0 | 33.3 | 100.0 | 100.0 | 85.7 |
| 3-Methylene-4-vinylcyclohex-1-ene | 66.7 | 100.0 | 66.7 | 100,0 | 33.3 | 100.0 | 100.0 | 81.0 |

Abbreviations: SC – *Saccharomyces cerevisiae*, SC×SPx – *Saccharomyces cerevisiae*×*Saccharomyces paradoxus* hybrid, TD+SC – *Torulaspora delbrueckii+S. cerevisiae*, MP+SC – *Metschnikowia pulcherrima+S. cerevisiae*, PK+SC – *Pichia kluyveri+S. cerevisiae,* LT+SC – *Lachancea thermotolerans+S. cerevisiae,* SP+SC – *Schizosaccharomyces pombe+S. cerevisiae*, TOT – TOTAL correct classification.

**
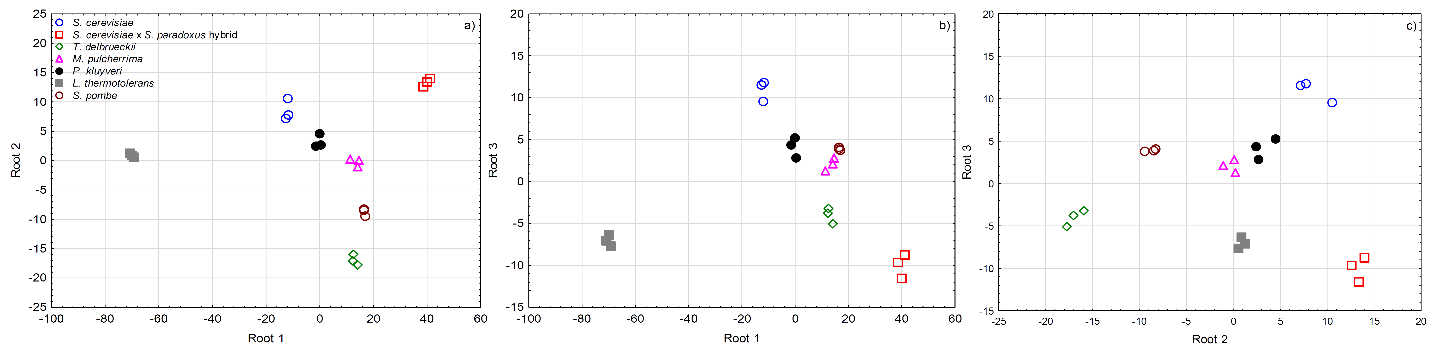
**Figure S2. Separation of Malvazija istarska wines according to yeast used in fermentation defined by the first three discriminant functions (roots) obtained by forward stepwise linear discriminant analysis (SLDA) on the basis of the composition of terpenoids determined by GC×GC/TOF-MS analysis.

Table S2. Variables from the group of terpenoids included in the SLDA model with their contribution to the correct classification (%) of Malvazija istarska wines according to yeast used in fermentation.

| Terpenoids | Wine | | | | | | | |
| --- | --- | --- | --- | --- | --- | --- | --- | --- |
|  | SC | SC×SPx | TD+SC | MP+SC | PK+SC | LT+SC | SP+SC | TOT |
| β-Bisabolene | 0.0 | 66.7 | 66.7 | 66.7 | 0.0 | 100.0 | 0.0 | 42.9 |
| Geranyl acetate | 66.7 | 66.7 | 66.7 | 100.0 | 66.7 | 100.0 | 33.3 | 71.4 |
| Citronellyl acetate | 100.0 | 100.0 | 100.0 | 100.0 | 66.7 | 100.0 | 100.0 | 95.2 |
| Epoxyterpinolene | 100.0 | 100.0 | 100.0 | 100.0 | 100.0 | 100.0 | 100.0 | 100.0 |
| Terpenoid n.i. | 100.0 | 100.0 | 100.0 | 100.0 | 100.0 | 100.0 | 100.0 | 100.0 |
| β-Myrcene | 100.0 | 100.0 | 100.0 | 100.0 | 100.0 | 100.0 | 100.0 | 100.0 |
| α-Calacorene | 100.0 | 100.0 | 100.0 | 100.0 | 100.0 | 100.0 | 100.0 | 100.0 |
| Isomyocorene | 100.0 | 100.0 | 100.0 | 100.0 | 100.0 | 100.0 | 100.0 | 100.0 |
| *trans*-Alloocimene | 100.0 | 100.0 | 100.0 | 100.0 | 100.0 | 100.0 | 100.0 | 100.0 |

Abbreviations: *SC* – *Saccharomyces cerevisiae*, *SC*×*SPx* – *Saccharomyces cerevisiae*×*Saccharomyces paradoxus* hybrid, *TD+SC* – *Torulaspora delbrueckii+S. cerevisiae*, MP+SC – *Metschnikowia pulcherrima+S. cerevisiae*, PK+SC – *Pichia kluyveri+S. cerevisiae,* LT+SC – *Lachancea thermotolerans+S. cerevisiae,* SP+SC – *Schizosaccharomyces pombe+S. cerevisiae*, TOT – TOTAL correct classification.


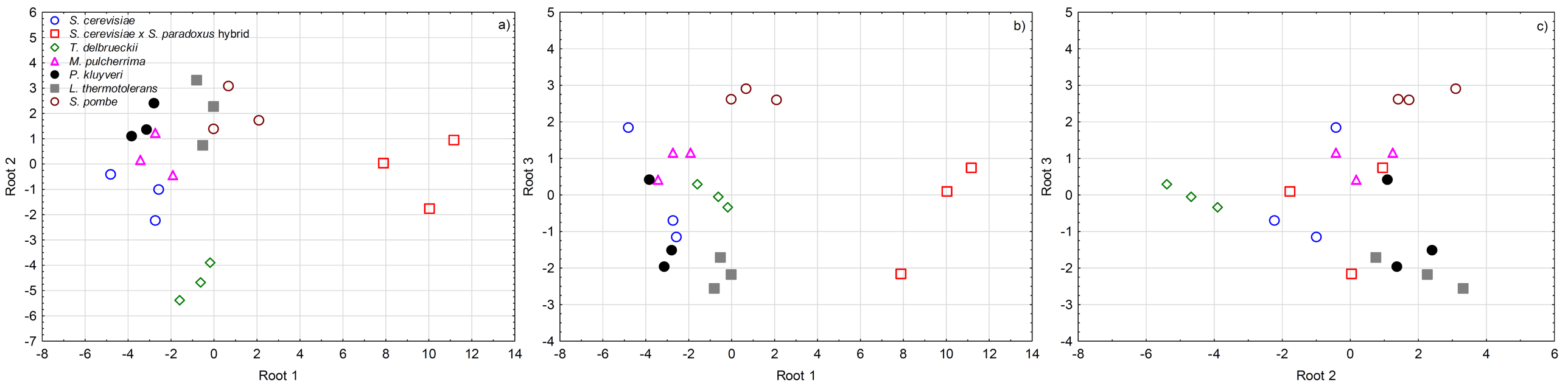
Figure S3. Separation of Malvazija istarska wines according to yeast used in fermentation defined by the first three discriminant functions (roots) obtained by forward stepwise linear discriminant analysis (SLDA) on the basis of the composition of norisoprenoids determined by GC×GC/TOF-MS analysis.

Table S3. Variables from the group of norisoprenoids included in the SLDA model with their contribution to the correct classification (%) of Malvazija istarska wines according to yeast used in fermentation.

| Norisoprenoids | Wine | | | | | | | |
| --- | --- | --- | --- | --- | --- | --- | --- | --- |
|  | SC | SC×SPx | TD+SC | MP+SC | PK+SC | LT+SC | SP+SC | TOT |
| Norisoprenoid n.i. | 0.0 | 66.7 | 100.0 | 0.0 | 66.7 | 0.0 | 100.0 | 47.6 |
| β-Cyclocitral | 66.7 | 100.0 | 66.7 | 33.3 | 33.3 | 33.3 | 100.0 | 61.9 |
| *cis*-β-Damascenone | 33.3 | 100.0 | 100.0 | 33.3 | 66.7 | 66.7 | 100.0 | 71.4 |
| α-Ionene | 100.0 | 100.0 | 100.0 | 66.7 | 66.7 | 100.0 | 100.0 | 90.5 |
| Vitispirane I | 100.0 | 100.0 | 100.0 | 100.0 | 100.0 | 100.0 | 100.0 | 100.0 |
| Theaspirane I | 100.0 | 100.0 | 100.0 | 100.0 | 100.0 | 100.0 | 100.0 | 100.0 |
| *trans*-1-(2,3,6-Trimethylphenyl)buta-1,3-diene (TPB) | 100.0 | 100.0 | 100.0 | 100.0 | 100.0 | 100.0 | 100.0 | 100.0 |

Abbreviations: *SC* – *Saccharomyces cerevisiae*, *SC*×*SPx* – *Saccharomyces cerevisiae*×*Saccharomyces paradoxus* hybrid, TD+SC – *Torulaspora delbrueckii+S. cerevisiae*, MP+SC – *Metschnikowia pulcherrima+S. cerevisiae*, PK+SC – *Pichia kluyveri+S. cerevisiae,* LT+SC – *Lachancea thermotolerans+S. cerevisiae,* SP+SC – *Schizosaccharomyces pombe+S. cerevisiae*, TOT – TOTAL correct classification.


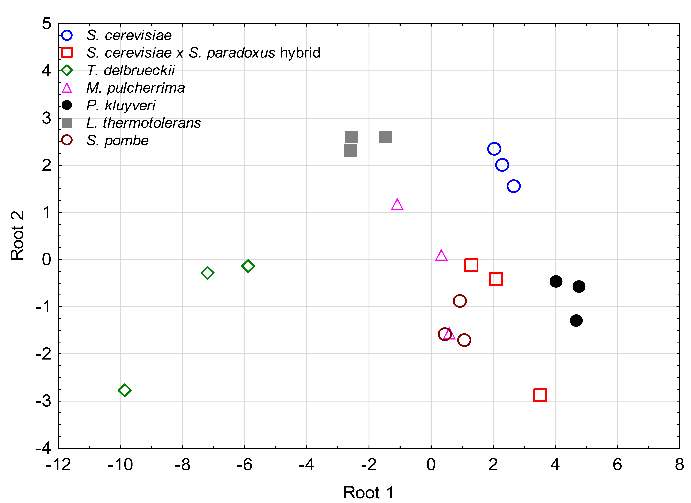


Figure S4. Separation of Malvazija istarska wines according to yeast used in fermentation defined by the first three discriminant functions (roots) obtained by forward stepwise linear discriminant analysis (SLDA) on the basis of the composition of thiols determined by GC/MS analysis.

Table S4. Variables from the group of thiols included in the SLDA model with their contribution to the correct classification (%) of Malvazija istarska wines according to yeast used in fermentation.

| Thiols | Wine | | | | | | | |
| --- | --- | --- | --- | --- | --- | --- | --- | --- |
|  | SC | SC×SPx | TD+SC | MP+SC | PK+SC | LT+SC | SP+SC | TOT |
| 3-Mercaptohexyl acetate (3MHA) | 100.0 | 100.0 | 100.0 | 66.7 | 66.7 | 66.7 | 66.7 | 81.0 |
| 3-Mercaptohexan-1-ol (MH) | 100.0 | 66.7 | 100.0 | 66.7 | 100.0 | 100.0 | 100.0 | 90.5 |

Abbreviations: SC – *Saccharomyces cerevisiae*, SC×SPx – *Saccharomyces cerevisiae*×*Saccharomyces paradoxus* hybrid, TD+SC – *Torulaspora delbrueckii+S. cerevisiae*, MP+SC – *Metschnikowia pulcherrima+S. cerevisiae*, PK+SC – *Pichia kluyveri+S. cerevisiae,* LT+SC – *Lachancea thermotolerans+S. cerevisiae,* SP+SC – *Schizosaccharomyces pombe+S. cerevisiae*, TOT – TOTAL correct classification.


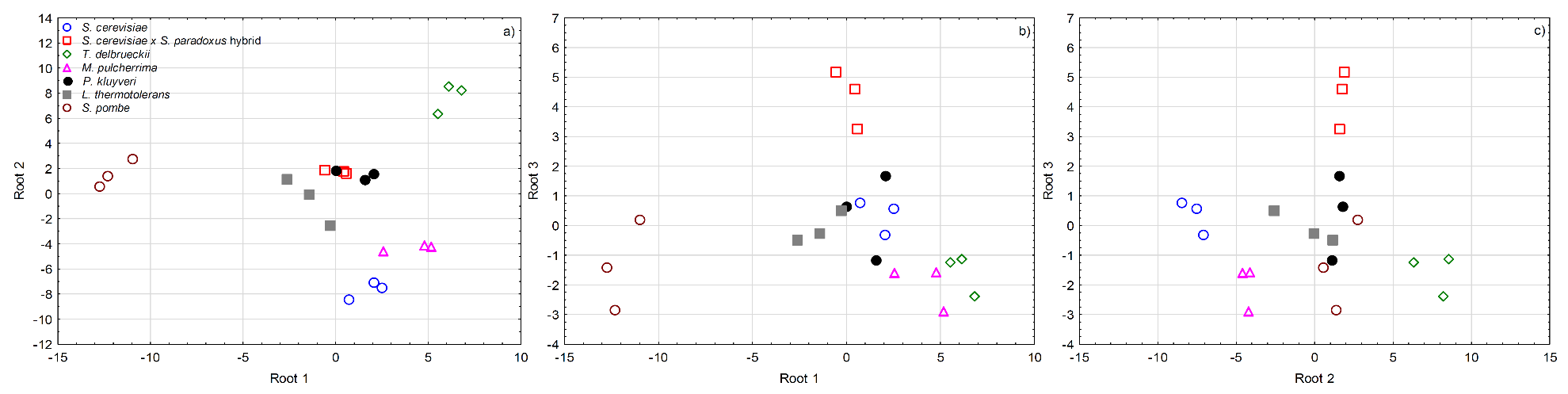


Figure S5. Separation of Malvazija istarska wines according to yeast used in fermentation defined by the first three discriminant functions (roots) obtained by forward stepwise linear discriminant analysis (SLDA) on the basis of the composition of aldehydes determined by GC/FID and GC×GC/TOF-MS analysis.

Table S5. Variables from the group of aldehydes included in the SLDA model with their contribution to the correct classification (%) of Malvazija istarska wines according to yeast used in fermentation.

| Aldehydes | Wine | | | | | | | |
| --- | --- | --- | --- | --- | --- | --- | --- | --- |
|  | SC | SC×SPx | TD+SC | MP+SC | PK+SC | LT+SC | SP+SC | TOT |
| Acetaldehyde | 100.0 | 100.0 | 100.0 | 0.0 | 100.0 | 0.0 | 66.7 | 66.7 |
| 2-Nonenal | 100.0 | 100.0 | 100.0 | 100.0 | 100.0 | 100.0 | 66.7 | 95.2 |
| 2-(Acetoxy)-propanal | 100.0 | 100.0 | 100.0 | 100.0 | 100.0 | 100.0 | 100.0 | 100.0 |
| Tetradecanal | 100.0 | 100.0 | 100.0 | 100.0 | 100.0 | 100.0 | 100.0 | 100.0 |
| 2,6,6-Trimethyl-1-cyclohexene-1-acrolein | 100.0 | 100.0 | 100.0 | 100.0 | 100.0 | 100.0 | 100.0 | 100.0 |
| Nonanal | 100.0 | 100.0 | 100.0 | 100.0 | 100.0 | 100.0 | 100.0 | 100.0 |
| Decanal | 100.0 | 100.0 | 100.0 | 100.0 | 100.0 | 100.0 | 100.0 | 100.0 |
| Octanal | 100.0 | 100.0 | 100.0 | 100.0 | 100.0 | 100.0 | 100.0 | 100.0 |
| Dodecanal | 100.0 | 100.0 | 100.0 | 100.0 | 100.0 | 100.0 | 100.0 | 100.0 |

Abbreviations: SC – *Saccharomyces cerevisiae*, SC×SPx – *Saccharomyces cerevisiae*×*Saccharomyces paradoxus* hybrid, TD+SC – *Torulaspora delbrueckii+S. cerevisiae*, MP+SC – *Metschnikowia pulcherrima+S. cerevisiae*, PK+SC – *Pichia kluyveri+S. cerevisiae,* LT+SC – *Lachancea thermotolerans+S. cerevisiae,* SP+SC – *Schizosaccharomyces pombe+S. cerevisiae*, TOT – TOTAL correct classification.


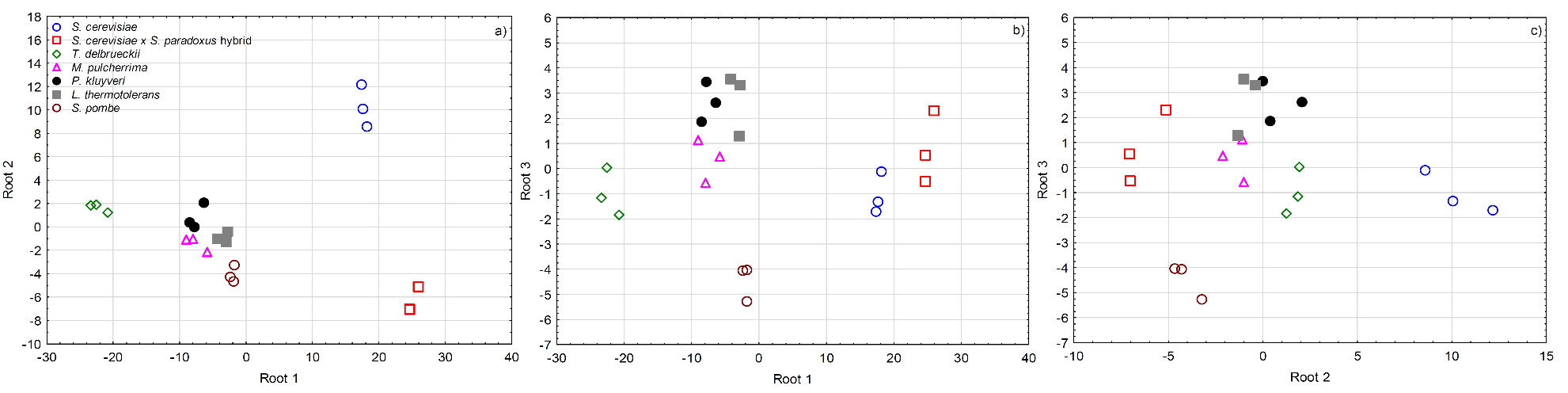
Figure S6. Separation of Malvazija istarska wines according to yeast used in fermentation defined by the first three discriminant functions (roots) obtained by forward stepwise linear discriminant analysis (SLDA) on the basis of the composition of ketones determined by GC×GC/TOF-MS analysis.

Table S6. Variables from the group of ketones included in the SLDA model with their contribution to the correct classification (%) of Malvazija istarska wines according to yeast used in fermentation.

| Ketones | Wine | | | | | | | |
| --- | --- | --- | --- | --- | --- | --- | --- | --- |
|  | SC | SC×SPx | TD+SC | MP+SC | PK+SC | LT+SC | SP+SC | TOT |
| 1,2-Dihydroxycyclobutene-3,4-dione | 100.0 | 100.0 | 100.0 | 33.3 | 66.7 | 66.7 | 100.0 | 81.0 |
| 2-Undecanone | 100.0 | 100.0 | 100.0 | 33.3 | 66.7 | 66.7 | 100.0 | 81.0 |
| 3-(Acetoxy)-4-methyl-2-pentanone | 100.0 | 100.0 | 100.0 | 66.7 | 66.7 | 66.7 | 100.0 | 85.7 |
| 2,3-Dihydro-3,3,4,5-tetramethyl-1H-inden-1-one | 100.0 | 100.0 | 100.0 | 66.7 | 100.0 | 100.0 | 100.0 | 95.2 |
| 2-Decanone | 100.0 | 100.0 | 100.0 | 100.0 | 100.0 | 100.0 | 100.0 | 100.0 |
| 6-Methyl-5-hepten-2-one | 100.0 | 100.0 | 100.0 | 100.0 | 100.0 | 100.0 | 100.0 | 100.0 |
| Acetoin | 100.0 | 100.0 | 100.0 | 100.0 | 100.0 | 100.0 | 100.0 | 100.0 |
| 2-Ethyl-1,6-dioxaspiro[4,4]nonane | 100.0 | 100.0 | 100.0 | 100.0 | 100.0 | 100.0 | 100.0 | 100.0 |

Abbreviations: SC – *Saccharomyces cerevisiae*, SC×SPx – *Saccharomyces cerevisiae*×*Saccharomyces paradoxus* hybrid, TD+SC – *Torulaspora delbrueckii+S. cerevisiae*, MP+SC – *Metschnikowia pulcherrima+S. cerevisiae*, PK+SC – *Pichia kluyveri+S. cerevisiae,* LT+SC – *Lachancea thermotolerans+S. cerevisiae,* SP+SC – *Schizosaccharomyces pombe+S. cerevisiae*, TOT – TOTAL correct classification.


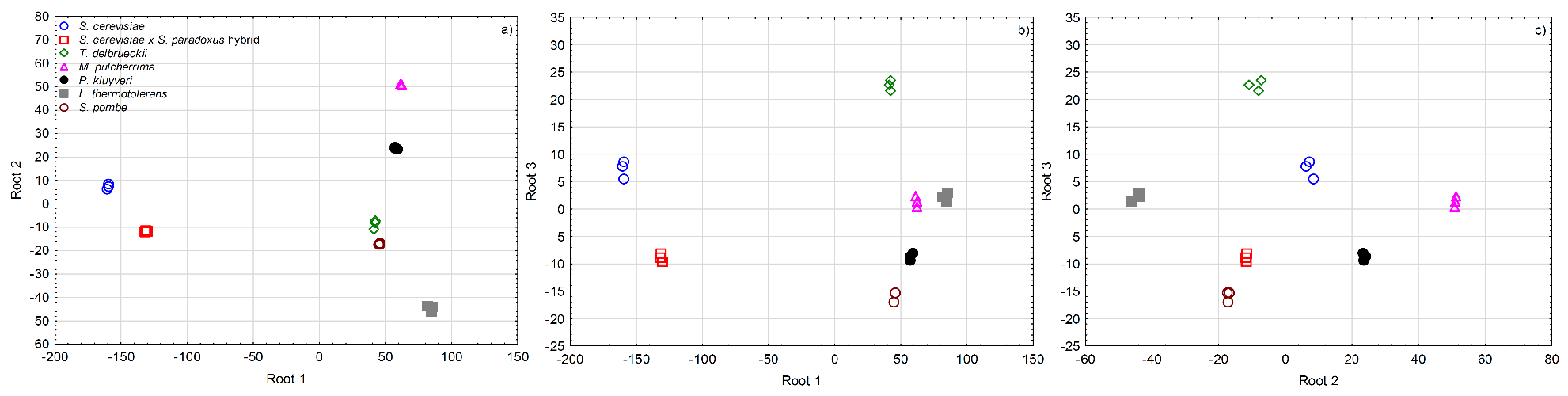


Figure S7. Separation of Malvazija istarska wines according to yeast used in fermentation defined by the first three discriminant functions (roots) obtained by forward stepwise linear discriminant analysis (SLDA) on the basis of the composition of alcohols determined by GC/FID, GC/MS and GC×GC/TOF-MS analysis.

Table S7. Variables from the group of alcohols included in the SLDA model with their contribution to the correct classification (%) of Malvazija istarska wines according to yeast used in fermentation.

| Alcohols | Wine | | | | | | | |
| --- | --- | --- | --- | --- | --- | --- | --- | --- |
|  | SC | SC×SPx | TD+SC | MP+SC | PK+SC | LT+SC | SP+SC | TOT |
| 3-Methylpentanol | 100.0 | 100.0 | 0.0 | 66.7 | 33.3 | 100.0 | 66.7 | 66.7 |
| 1-Propanol | 100.0 | 100.0 | 100.0 | 66.7 | 100.0 | 100.0 | 33.3 | 85.7 |
| 2-Phenylethanol | 100.0 | 100.0 | 100.0 | 100.0 | 100.0 | 100.0 | 100.0 | 100.0 |
| 2-Undecanol | 100.0 | 100.0 | 100.0 | 100.0 | 100.0 | 100.0 | 100.0 | 100.0 |
| 4-Allyl-1,6-Heptadiene-4-ol | 100.0 | 100.0 | 100.0 | 100.0 | 100.0 | 100.0 | 100.0 | 100.0 |
| 4-*tert*-Butylcyclohexanol | 100.0 | 100.0 | 100.0 | 100.0 | 100.0 | 100.0 | 100.0 | 100.0 |
| 2-Ethyl-1-hexanol | 100.0 | 100.0 | 100.0 | 100.0 | 100.0 | 100.0 | 100.0 | 100.0 |
| 1-Hexanol | 100.0 | 100.0 | 100.0 | 100.0 | 100.0 | 100.0 | 100.0 | 100.0 |
| Isobutanol | 100.0 | 100.0 | 100.0 | 100.0 | 100.0 | 100.0 | 100.0 | 100.0 |
| 3-Nonanol | 100.0 | 100.0 | 100.0 | 100.0 | 100.0 | 100.0 | 100.0 | 100.0 |
| *trans*-3-Hexen-1-ol | 100.0 | 100.0 | 100.0 | 100.0 | 100.0 | 100.0 | 100.0 | 100.0 |
| 3-Octanol | 100.0 | 100.0 | 100.0 | 100.0 | 100.0 | 100.0 | 100.0 | 100.0 |

Abbreviations: SC – *Saccharomyces cerevisiae*, SC×SPx – *Saccharomyces cerevisiae*×*Saccharomyces paradoxus* hybrid, TD+SC – *Torulaspora delbrueckii+S. cerevisiae*, MP+SC – *Metschnikowia pulcherrima+S. cerevisiae*, PK+SC – *Pichia kluyveri+S. cerevisiae,* LT+SC – *Lachancea thermotolerans+S. cerevisiae,* SP+SC – *Schizosaccharomyces pombe+S. cerevisiae*, TOT – TOTAL correct classification.


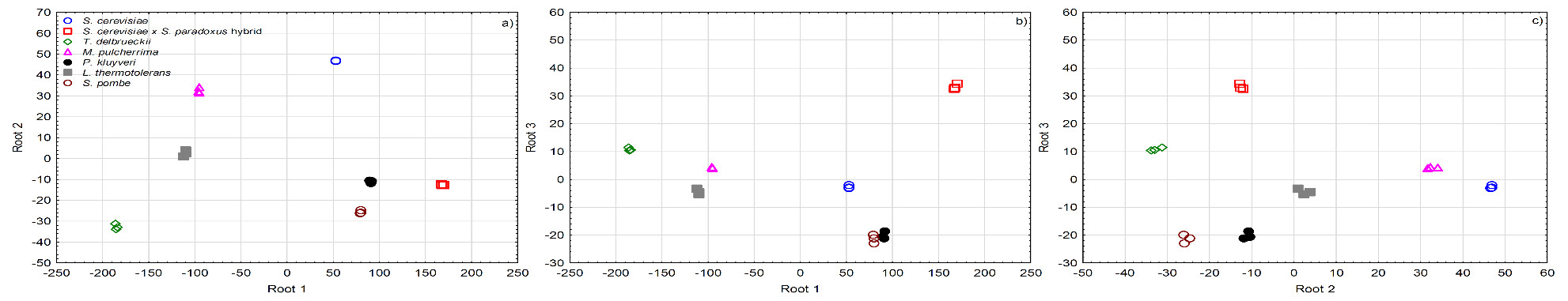


Figure S8. Separation of Malvazija istarska wines according to yeast used in fermentation defined by the first three discriminant functions (roots) obtained by forward stepwise linear discriminant analysis (SLDA) on the basis of the composition of acids determined by GC/MS and GC×GC/TOF-MS analysis.

Table S8. Variables from the group of acids included in the SLDA model with their contribution to the correct classification (%) of Malvazija istarska wines according to yeast used in fermentation.

| Acids | Wine | | | | | | | |
| --- | --- | --- | --- | --- | --- | --- | --- | --- |
|  | SC | SC×SPx | TD+SC | MP+SC | PK+SC | LT+SC | SP+SC | TOT |
| 2-Methylbutyric acid | 100.0 | 100.0 | 66.7 | 33.3 | 33.3 | 100 | 100.0 | 76.2 |
| Isovaleric acid | 100.0 | 100.0 | 100.0 | 100.0 | 100.0 | 100.0 | 100.0 | 100.0 |
| Isobutyric acid | 100.0 | 100.0 | 100.0 | 100.0 | 100.0 | 100.0 | 100.0 | 100.0 |
| *trans*-2-Hexenoic acid | 100.0 | 100.0 | 100.0 | 100.0 | 100.0 | 100.0 | 100.0 | 100.0 |
| 3-Octenoic acid | 100.0 | 100.0 | 100.0 | 100.0 | 100.0 | 100.0 | 100.0 | 100.0 |
| 2-Propenoic acid | 100.0 | 100.0 | 100.0 | 100.0 | 100.0 | 100.0 | 100.0 | 100.0 |
| Heptanoic acid | 100.0 | 100.0 | 100.0 | 100.0 | 100.0 | 100.0 | 100.0 | 100.0 |
| Pivalic acid | 100.0 | 100.0 | 100.0 | 100.0 | 100.0 | 100.0 | 100.0 | 100.0 |
| Acetic acid | 100.0 | 100.0 | 100.0 | 100.0 | 100.0 | 100.0 | 100.0 | 100.0 |
| Butyric acid | 100.0 | 100.0 | 100.0 | 100.0 | 100.0 | 100.0 | 100.0 | 100.0 |
| 2-Ethylhexanoic acid | 100.0 | 100.0 | 100.0 | 100.0 | 100.0 | 100.0 | 100.0 | 100.0 |
| Nonanoic acid | 100.0 | 100.0 | 100.0 | 100.0 | 100.0 | 100.0 | 100.0 | 100.0 |
| *cis*-2-Octanoic acid | 100.0 | 100.0 | 100.0 | 100.0 | 100.0 | 100.0 | 100.0 | 100.0 |

Abbreviations: SC – *Saccharomyces cerevisiae*, SC×SPx – *Saccharomyces cerevisiae*×*Saccharomyces paradoxus* hybrid, TD+SC – *Torulaspora delbrueckii+S. cerevisiae*, MP+SC – *Metschnikowia pulcherrima+S. cerevisiae*, PK+SC – *Pichia kluyveri+S. cerevisiae,* LT+SC – *Lachancea thermotolerans+S. cerevisiae,* SP+SC – *Schizosaccharomyces pombe+S. cerevisiae*, TOT – TOTAL correct classification.


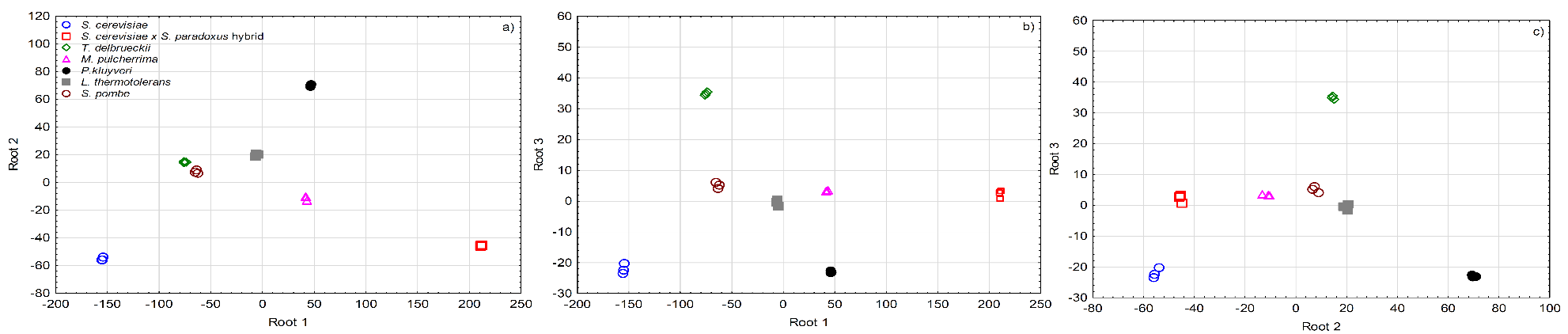


Figure S9. Separation of Malvazija istarska wines according to yeast used in fermentation defined by the first three discriminant functions (roots) obtained by forward stepwise linear discriminant analysis (SLDA) on the basis of the composition of ethyl esters determined by GC/MS and GC×GC/TOF-MS analysis.

Table S9. Variables from the group of ethyl esters included in the SLDA model with their contribution to the correct classification (%) of Malvazija istarska wines accoding to yeast used in fermentation.

| Ethyl esters | Wine | | | | | | | |
| --- | --- | --- | --- | --- | --- | --- | --- | --- |
|  | SC | SC×SPx | TD+SC | MP+SC | PK+SC | LT+SC | SP+SC | TOT |
| Ethyl propanoate | 66.7 | 66.7 | 100.0 | 33.3 | 66.7 | 33.3 | 100.0 | 66.7 |
| Ethyl 3-methylbutyrate | 66.7 | 66.7 | 100.0 | 100.0 | 66.7 | 100.0 | 100.0 | 85.7 |
| Ethyl 2-hexenoate II | 100.0 | 100.0 | 100.0 | 100.0 | 100.0 | 100.0 | 100.0 | 100.0 |
| Ethyl isoamyl succinate | 100.0 | 100.0 | 100.0 | 100.0 | 100.0 | 100.0 | 100.0 | 100.0 |
| Ethyl butyl succinate | 100.0 | 100.0 | 100.0 | 100.0 | 100.0 | 100.0 | 100.0 | 100.0 |
| Ethyl 3-hydroxydecanoate | 100.0 | 100.0 | 100.0 | 100.0 | 100.0 | 100.0 | 100.0 | 100.0 |
| Ethyl 3-hydroxybutyrate | 100.0 | 100.0 | 100.0 | 100.0 | 100.0 | 100.0 | 100.0 | 100.0 |
| Ethyl *tran*s-4-octenoate | 100.0 | 100.0 | 100.0 | 100.0 | 100.0 | 100.0 | 100.0 | 100.0 |
| Ethyl *cis*-11-hexadecenoate | 100.0 | 100.0 | 100.0 | 100.0 | 100.0 | 100.0 | 100.0 | 100.0 |
| Ethyl decanoate | 100.0 | 100.0 | 100.0 | 100.0 | 100.0 | 100.0 | 100.0 | 100.0 |
| Ethyl *trans*-2-butenoate | 100.0 | 100.0 | 100.0 | 100.0 | 100.0 | 100.0 | 100.0 | 100.0 |
| Ethyl 7-octenoate | 100.0 | 100.0 | 100.0 | 100.0 | 100.0 | 100.0 | 100.0 | 100.0 |

Abbreviations: SC – *Saccharomyces cerevisiae*, SC×SPx – *Saccharomyces cerevisiae*×*Saccharomyces paradoxus* hybrid, TD+SC – *Torulaspora delbrueckii+S. cerevisiae*, MP+SC – *Metschnikowia pulcherrima+S. cerevisiae*, PK+SC – *Pichia kluyveri+S. cerevisiae,* LT+SC – *Lachancea thermotolerans+S. cerevisiae,* SP+SC – *Schizosaccharomyces pombe+S. cerevisiae*, TOT – TOTAL correct classification.


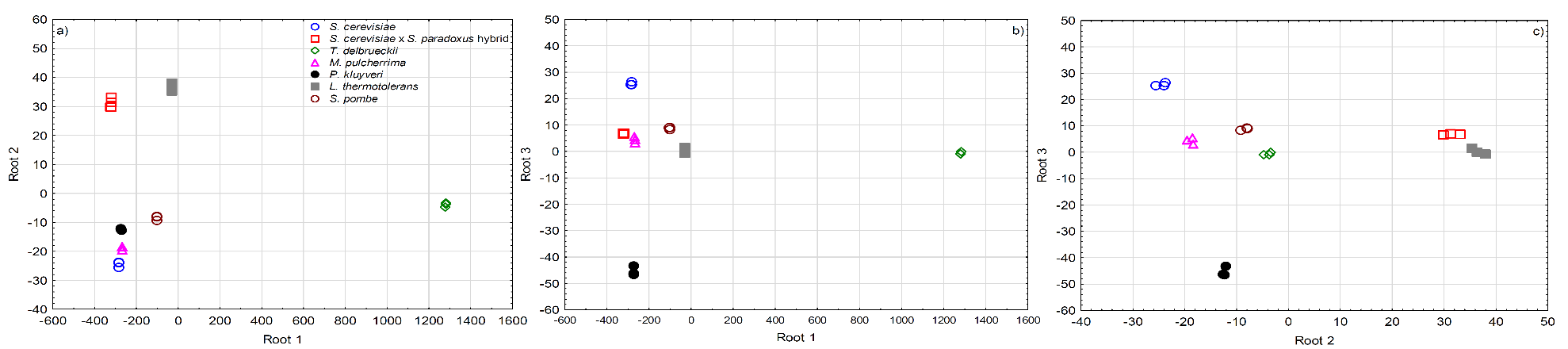


Figure S10. Separation of Malvazija istarska wines according to yeast used in fermentation defined by the first three discriminant functions (roots) obtained by forward stepwise linear discriminant analysis (SLDA) on the basis of the composition of acetate esters determined by GC/FID, GC/MS and GC×GC/TOF-MS analysis.

Table S10. Variables from the group of acetate esters included in the SLDA model with their contribution to the correct classification (%) of Malvazija istarska wines according to yeast used in fermentation.

| Acetate esters | Wine | | | | | | | |
| --- | --- | --- | --- | --- | --- | --- | --- | --- |
|  | SC | SC×SPx | TD+SC | MP+SC | PK+SC | LT+SC | SP+SC | TOT |
| 3-Ethoxypropyl acetate | 100.0 | 100.0 | 100.0 | 100.0 | 100.0 | 100.0 | 66.7 | 95.2 |
| Diol acetate n.i. | 100.0 | 100.0 | 100.0 | 66.7 | 66.7 | 100.0 | 66.7 | 85.7 |
| *trans*-3-Hexen-1-yl acetate | 100.0 | 100.0 | 100.0 | 100.0 | 100.0 | 100.0 | 100.0 | 100.0 |
| 2-Phenetyl acetate | 100.0 | 100.0 | 100.0 | 100.0 | 100.0 | 100.0 | 100.0 | 100.0 |
| *cis*-6-Nonen-1-yl acetate | 100.0 | 100.0 | 100.0 | 100.0 | 100.0 | 100.0 | 100.0 | 100.0 |
| Butyl acetate | 100.0 | 100.0 | 100.0 | 100.0 | 100.0 | 100.0 | 100.0 | 100.0 |
| Propyl acetate | 100.0 | 100.0 | 100.0 | 100.0 | 100.0 | 100.0 | 100.0 | 100.0 |
| 3-Methyl-3-buten-1-yl acetate | 100.0 | 100.0 | 100.0 | 100.0 | 100.0 | 100.0 | 100.0 | 100.0 |
| Hexyl acetate | 100.0 | 100.0 | 100.0 | 100.0 | 100.0 | 100.0 | 100.0 | 100.0 |
| Methyl acetate | 100.0 | 100.0 | 100.0 | 100.0 | 100.0 | 100.0 | 100.0 | 100.0 |
| Ethyl acetate | 100.0 | 100.0 | 100.0 | 100.0 | 100.0 | 100.0 | 100.0 | 100.0 |
| (*trans, trans*)-2,4-Octadienyl acetate | 100.0 | 100.0 | 100.0 | 100.0 | 100.0 | 100.0 | 100.0 | 100.0 |
| Isoamyl acetate | 100.0 | 100.0 | 100.0 | 100.0 | 100.0 | 100.0 | 100.0 | 100.0 |

Abbreviations: SC – *Saccharomyces cerevisiae*, SC×SPx – *Saccharomyces cerevisiae*×*Saccharomyces paradoxus* hybrid, TD+SC – *Torulaspora delbrueckii+S. cerevisiae*, MP+SC – *Metschnikowia pulcherrima+S. cerevisiae*, PK+SC – *Pichia kluyveri+S. cerevisiae,* LT+SC – *Lachancea thermotolerans+S. cerevisiae,* SP+SC – *Schizosaccharomyces pombe+S. cerevisiae*, TOT – TOTAL correct classification.


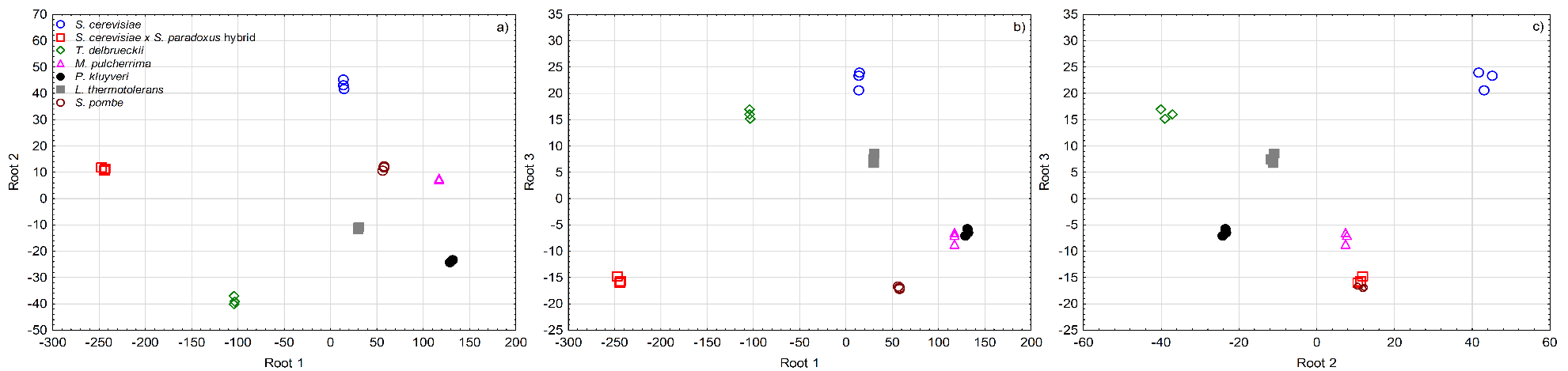


Figure S11. Separation of Malvazija istarska wines according to yeast used in fermentation defined by the first three discriminant functions (roots) obtained by forward stepwise linear discriminant analysis (SLDA) on the basis of the composition of other esters determined by GC/FID, GC/MS and GC×GC/TOF-MS analysis.

Table S11. Variables from the group of other esters included in the SLDA model with their contribution to the correct classification (%) of Malvazija istarska wines according to yeast used in fermentation.

| Other esters | Wine | | | | | | | |
| --- | --- | --- | --- | --- | --- | --- | --- | --- |
|  | SC | SC×SPx | TD+SC | MP+SC | PK+SC | LT+SC | SP+SC | TOT |
| β-Phenethyl formate | 66.7 | 100.0 | 33.3 | 33.3 | 66.7 | 100.0 | 66.7 | 66.7 |
| Methyl pyruvate | 66.7 | 100.0 | 100.0 | 33.3 | 100.0 | 100.0 | 100.0 | 85.7 |
| Diethyl malate | 100.0 | 100.0 | 100.0 | 100.0 | 100.0 | 100.0 | 100.0 | 100.0 |
| Isobutyl octanoate | 100.0 | 100.0 | 100.0 | 100.0 | 100.0 | 100.0 | 100.0 | 100.0 |
| Hexadecyl hexanoate | 100.0 | 100.0 | 100.0 | 100.0 | 100.0 | 100.0 | 100.0 | 100.0 |
| Isoamyl lactate | 100.0 | 100.0 | 100.0 | 100.0 | 100.0 | 100.0 | 100.0 | 100.0 |
| Amyl methacrylate | 100.0 | 100.0 | 100.0 | 100.0 | 100.0 | 100.0 | 100.0 | 100.0 |
| Diethyl glutarate | 100.0 | 100.0 | 100.0 | 100.0 | 100.0 | 100.0 | 100.0 | 100.0 |
| Diethyl fumarate | 100.0 | 100.0 | 100.0 | 100.0 | 100.0 | 100.0 | 100.0 | 100.0 |
| Isoamyl decanoate | 100.0 | 100.0 | 100.0 | 100.0 | 100.0 | 100.0 | 100.0 | 100.0 |
| Hexyl propyl oxalate | 100.0 | 100.0 | 100.0 | 100.0 | 100.0 | 100.0 | 100.0 | 100.0 |
| 2-Ethenylphenyl acetate | 100.0 | 100.0 | 100.0 | 100.0 | 100.0 | 100.0 | 100.0 | 100.0 |

Abbreviations: SC – *Saccharomyces cerevisiae*, SC×SPx – *Saccharomyces cerevisiae*×*Saccharomyces paradoxus* hybrid, TD+SC – *Torulaspora delbrueckii+S. cerevisiae*, MP+SC – *Metschnikowia pulcherrima+S. cerevisiae*, PK+SC – *Pichia kluyveri+S. cerevisiae,* LT+SC – *Lachancea thermotolerans+S. cerevisiae,* SP+SC – *Schizosaccharomyces pombe+S. cerevisiae*, TOT – TOTAL correct classification.


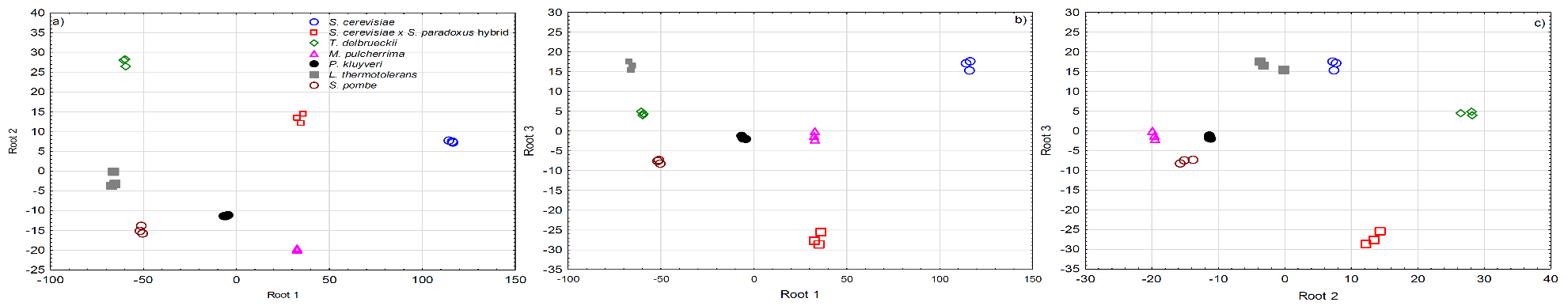


Figure S12. Separation of Malvazija istarska wines according to yeast used in fermentation defined by the first three discriminant functions (roots) obtained by forward stepwise linear discriminant analysis (SLDA) on the basis of the composition of sulfur containing compounds determined by GC/MS and GC×GC/TOF-MS analysis.

Table S12. Variables from the group of sulfur containing compounds included in the SLDA model with their contribution to the correct classification (%) of Malvazija istarska wines according to yeast used in fermentation.

| Sulfur containing compounds | Wine | | | | | | | |
| --- | --- | --- | --- | --- | --- | --- | --- | --- |
|  | SC | SC×SPx | TD+SC | MP+SC | PK+SC | LT+SC | SP+SC | TOT |
| 3-Hydroxyethyl 3-hydroxypropyl sulfide I | 100.0 | 66.7 | 100.0 | 0.0 | 66.7 | 66.7 | 66.7 | 66.7 |
| 3-Hydroxyethyl 3-hydroxypropyl sulfide II | 100.0 | 100.0 | 100.0 | 66.7 | 66.7 | 100.0 | 100.0 | 90.5 |
| Ethyl 3-methylthiopropanoate | 100.0 | 100.0 | 100.0 | 100.0 | 100.0 | 100.0 | 100.0 | 100.0 |
| Methionol | 100.0 | 100.0 | 100.0 | 100.0 | 100.0 | 100.0 | 100.0 | 100.0 |
| Mercapto-2-propanone | 100.0 | 100.0 | 100.0 | 100.0 | 100.0 | 100.0 | 100.0 | 100.0 |
| 3-Methylthio acetate | 100.0 | 100.0 | 100.0 | 100.0 | 100.0 | 100.0 | 100.0 | 100.0 |
| S-ethyl octanethioate | 100.0 | 100.0 | 100.0 | 100.0 | 100.0 | 100.0 | 100.0 | 100.0 |
| Propyl ethynyl sulfoxide | 100.0 | 100.0 | 100.0 | 100.0 | 100.0 | 100.0 | 100.0 | 100.0 |
| Ethyl methanesulfonate | 100.0 | 100.0 | 100.0 | 100.0 | 100.0 | 100.0 | 100.0 | 100.0 |
| 2-Thiophenecarboxaldehyde | 100.0 | 100.0 | 100.0 | 100.0 | 100.0 | 100.0 | 100.0 | 100.0 |
| Ethyl methanesulfonate | 100.0 | 100.0 | 100.0 | 100.0 | 100.0 | 100.0 | 100.0 | 100.0 |
| Dihydro-2-methyl-3(2H)-thiophenone | 100.0 | 100.0 | 100.0 | 100.0 | 100.0 | 100.0 | 100.0 | 100.0 |
| 2-(Methylmercapto) benzothiazole | 100.0 | 100. | 100.0 | 100.0 | 100.0 | 100.0 | 100.0 | 100.0 |

Abbreviations: SC – *Saccharomyces cerevisiae*, SC×SPx – *Saccharomyces cerevisiae*×*Saccharomyces paradoxus* hybrid, TD+SC – *Torulaspora delbrueckii+S. cerevisiae*, MP+SC – *Metschnikowia pulcherrima+S. cerevisiae*, PK+SC – *Pichia kluyveri+S. cerevisiae,* LT+SC – *Lachancea thermotolerans+S. cerevisiae,* SP+SC – *Schizosaccharomyces pombe+S. cerevisiae*, TOT – TOTAL correct classification.


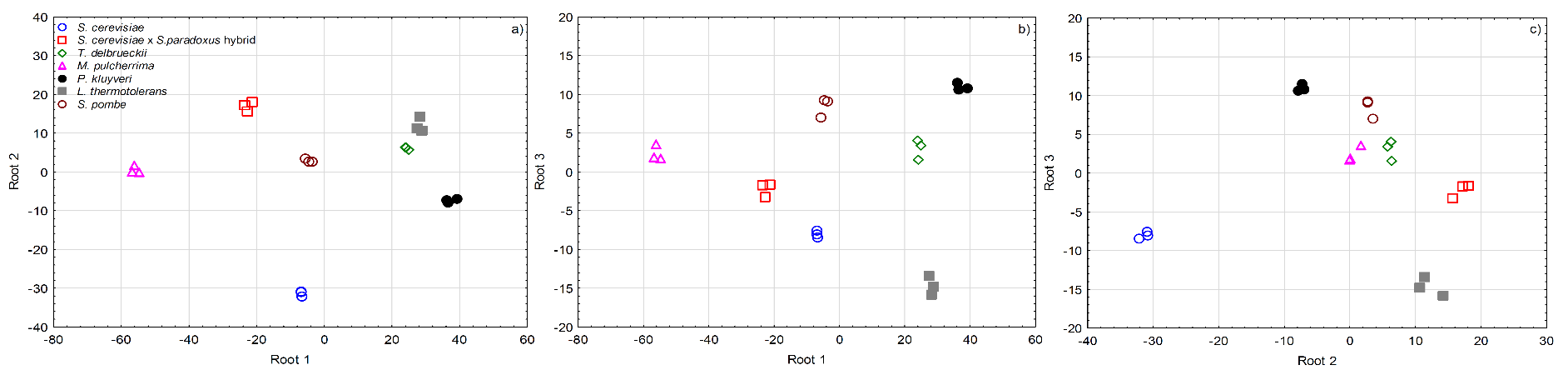


Figure S13. Separation of Malvazija istarska wines according to yeast used in fermentation defined by the first three discriminant functions (roots) obtained by forward stepwise linear discriminant analysis (SLDA) on the basis of the composition of furanoids and lactones determined by GC×GC/TOF-MS analysis.

Table S13. Variables from the group of furanoids and lactones included in the SLDA model with their contribution to the correct classification (%) of Malvazija istarska wines according to yeast used in fermentation.

| Furanoids and lactones | Wine | | | | | | | |
| --- | --- | --- | --- | --- | --- | --- | --- | --- |
|  | SC | SC×SPx | TD+SC | MP+SC | PK+SC | LT+SC | SP+SC | TOT |
| γ-Butyrolactone | 100.0 | 33.3 | 33.3 | 0.0 | 100.0 | 66.7 | 66.7 | 57.1 |
| γ-Decalactone | 100.0 | 100.0 | 66.7 | 66.7 | 100.0 | 100.0 | 66.7 | 85.7 |
| γ-Nonalactone | 100.0 | 100.0 | 100.0 | 100.0 | 100.0 | 100.0 | 66.7 | 95.2 |
| ß-Methyl-γ-butyro-lactone | 100.0 | 100.0 | 100.0 | 100.0 | 100.0 | 100.0 | 100.0 | 100.0 |
| 2-Ethyl furoate | 100.0 | 100.0 | 100.0 | 100.0 | 100.0 | 100.0 | 100.0 | 100.0 |
| Furfural | 100.0 | 100.0 | 100.0 | 100.0 | 100.0 | 100.0 | 100.0 | 100.0 |
| 2-Butyltetrahydrofuran | 100.0 | 100.0 | 100.0 | 100.0 | 100.0 | 100.0 | 100.0 | 100.0 |
| 2,2,4-trimethyl-5-(2,2-dimethylpropyl)-3(2H)-furanone | 100.0 | 100.0 | 100.0 | 100.0 | 100.0 | 100.0 | 100.0 | 100.0 |
| 4-Methyl-2-butenoic acid γ-lactone | 100.0 | 100.0 | 100.0 | 100.0 | 100.0 | 100.0 | 100.0 | 100.0 |
| δ-Lactone (n.i.) | 100.0 | 100.0 | 100.0 | 100.0 | 100.0 | 100.0 | 100.0 | 100.0 |
| 4-(1-Hydroxyethyl)- γ-butyrolactone | 100.0 | 100.0 | 100.0 | 100.0 | 100.0 | 100.0 | 100.0 | 100.0 |
| 2,5-Furandicarboxaldehyde | 100.0 | 100.0 | 100.0 | 100.0 | 100.0 | 100.0 | 100.0 | 100.0 |

Abbreviations: SC – *Saccharomyces cerevisiae*, SC×SPx – *Saccharomyces cerevisiae*×*Saccharomyces paradoxus* hybrid, TD+SC – *Torulaspora delbrueckii+S. cerevisiae*, MP+SC – *Metschnikowia pulcherrima+S. cerevisiae*, PK+SC – *Pichia kluyveri+S. cerevisiae,* LT+SC – *Lachancea thermotolerans+S. cerevisiae,* SP+SC – *Schizosaccharomyces pombe+S. cerevisiae*, TOT – TOTAL correct classification.


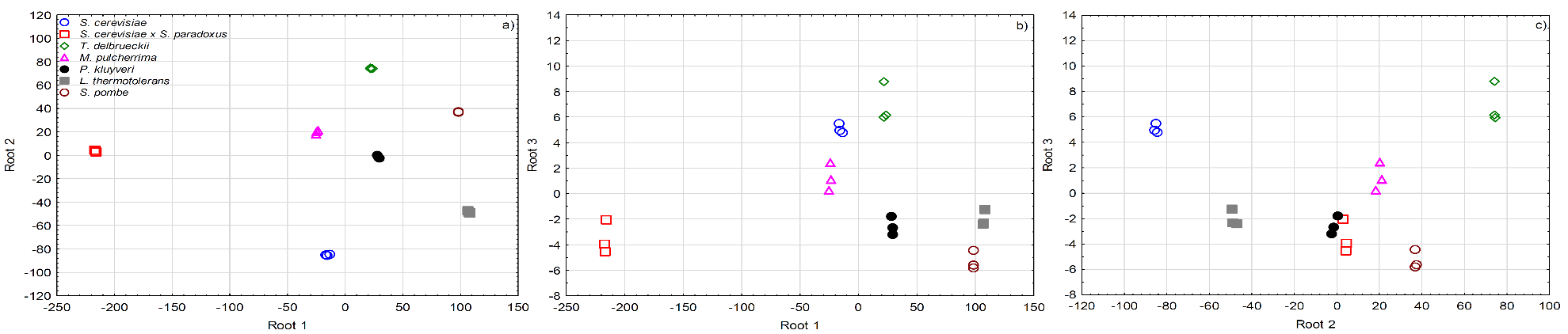


Figure S14. Separation of Malvazija istarska wines according to yeast used in fermentation defined by the first three discriminant functions (roots) obtained by forward stepwise linear discriminant analysis (SLDA) on the basis of the composition of benzenoids determined by GC×GC/TOF-MS analysis.

Table S14. Variables from the group of benzenoids included in the SLDA model with their contribution to the correct classification (%) of Malvazija istarska wines according to yeast used in fermentation.

| Benzenoids | Wine | | | | | | | |
| --- | --- | --- | --- | --- | --- | --- | --- | --- |
|  | SC | SC×SPx | TD+SC | MP+SC | PK+SC | LT+SC | SP+SC | TOT |
| Benzeneacetaldehyde | 33.3 | 100.0 | 33.3 | 66.7 | 33.3 | 66.7 | 33.3 | 52.4 |
| *p*-Ethylstyrene | 66.7 | 100.0 | 100.0 | 66.7 | 100.0 | 100.0 | 100.0 | 90.5 |
| Benzenoid n.i. | 66.7 | 100.0 | 100.0 | 66.7 | 66.7 | 100.0 | 66.7 | 81.0 |
| 2-Ethyl-*m*-xylene | 100.0 | 100.0 | 100.0 | 100.0 | 100.0 | 100.0 | 100.0 | 100.0 |
| Ethyl benzene | 100.0 | 100.0 | 100.0 | 100.0 | 100.0 | 100.0 | 100.0 | 100.0 |
| 2-(2-Phenylethoxy)propanal | 100.0 | 100.0 | 100.0 | 100.0 | 100.0 | 100.0 | 100.0 | 100.0 |
| Ethyl benzeneacetate | 100.0 | 100.0 | 100.0 | 100.0 | 100.0 | 100.0 | 100.0 | 100.0 |
| Methyl salicylate | 100.0 | 100.0 | 100.0 | 100.0 | 100.0 | 100.0 | 100.0 | 100.0 |
| (4-Methylphenyl) methanol, neopentyl ether | 100.0 | 100.0 | 100.0 | 100.0 | 100.0 | 100.0 | 100.0 | 100.0 |
| 3-(1-Methylethyl)benzoic acid | 100.0 | 100.0 | 100.0 | 100.0 | 100.0 | 100.0 | 100.0 | 100.0 |
| Acetaldehyde ethyl phenethyl acetal | 100.0 | 100.0 | 100.0 | 100.0 | 100.0 | 100.0 | 100.0 | 100.0 |
| (3-Methylphenyl) methanol, 2-methylpropyl ether | 100.0 | 100.0 | 100.0 | 100.0 | 100.0 | 100.0 | 100.0 | 100.0 |

Abbreviations: SC – *Saccharomyces cerevisiae*, SC×SPx – *Saccharomyces cerevisiae*×*Saccharomyces paradoxus* hybrid, TD+SC – *Torulaspora delbrueckii+S. cerevisiae*, MP+SC – *Metschnikowia pulcherrima+S. cerevisiae*, PK+SC – *Pichia kluyveri+S. cerevisiae,* LT+SC – *Lachancea thermotolerans+S. cerevisiae,* SP+SC – *Schizosaccharomyces pombe+S. cerevisiae*, TOT – TOTAL correct classification.


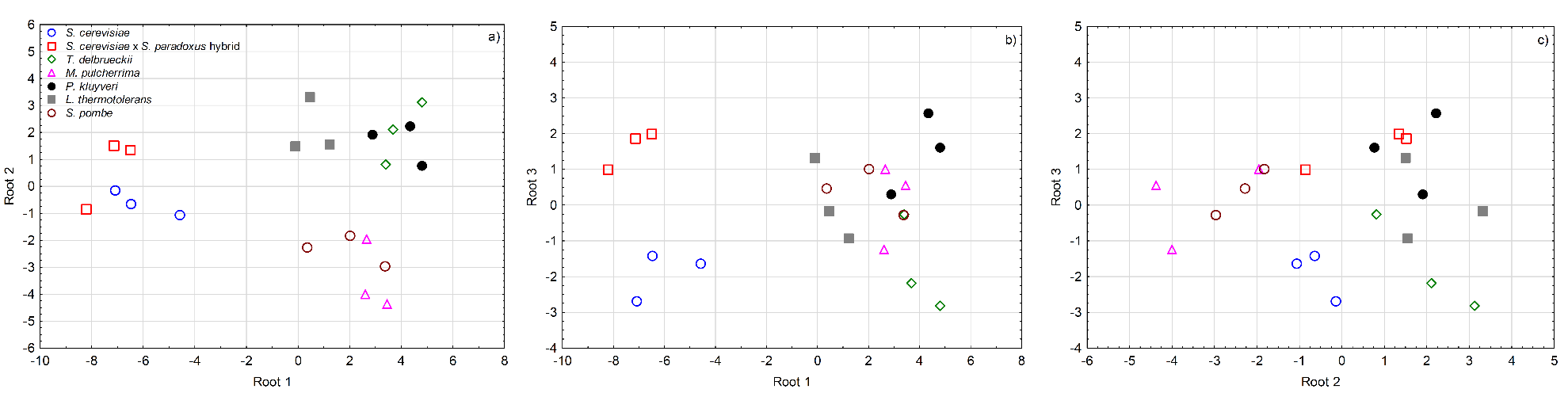
Figure S15. Separation of Malvazija istarska wines according to yeast used in fermentation defined by the first three discriminant functions (roots) obtained by forward stepwise linear discriminant analysis (SLDA) on the basis of the composition of volatile phenols determined by GC×GC/TOF-MS analysis.

Table S15. Variables from the group of volatile phenols included in the SLDA model with their contribution to the correct classification (%) of Malvazija istarska wines according to yeast used in fermentation.

| Volatile phenols | Wine | | | | | | | |
| --- | --- | --- | --- | --- | --- | --- | --- | --- |
|  | SC | SC×SPx | TD+SC | MP+SC | PK+SC | LT+SC | SP+SC | TOT |
| 4-Vinylguaiacol | 100.0 | 66.7 | 66.7 | 33.3 | 66.7 | 100.0 | 33.3 | 66.7 |
| 4-Vinylphenol | 100.0 | 66.7 | 100.0 | 100.0 | 66.7 | 100.0 | 66.7 | 85.7 |
| Guaiacol | 100.0 | 66.7 | 100.0 | 100.0 | 66.7 | 100.0 | 66.7 | 85.7 |
| *o*-Cresol | 100.0 | 100.0 | 66.7 | 100.0 | 100.0 | 100.0 | 66.7 | 90.5 |
| 2.3.6-Trimethylphenol | 100.0 | 100.0 | 100.0 | 100.0 | 66.7 | 100.0 | 66.7 | 90.5 |
| *p-tert*-Amylphenol | 100.0 | 100.0 | 100.0 | 100.0 | 100.0 | 100.0 | 100.0 | 100.0 |

Abbreviations: SC – *Saccharomyces cerevisiae*, SC×SPx – *Saccharomyces cerevisiae*×*Saccharomyces paradoxus* hybrid, TD+SC – *Torulaspora delbrueckii+S. cerevisiae*, MP+SC – *Metschnikowia pulcherrima+S. cerevisiae*, PK+SC – *Pichia kluyveri+S. cerevisiae,* LT+SC – *Lachancea thermotolerans+S. cerevisiae,* SP+SC – *Schizosaccharomyces pombe+S. cerevisiae*, TOT – TOTAL correct classification.


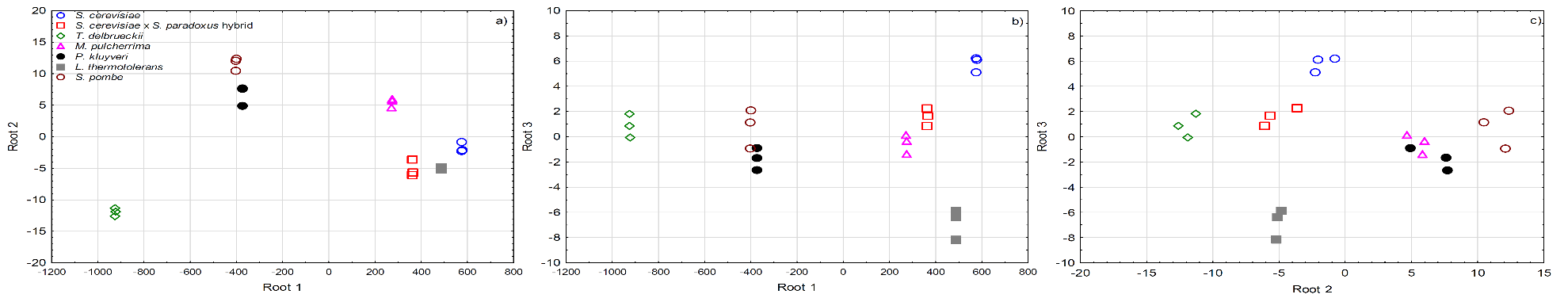


Figure S16. Separation of Malvazija istarska wines according to yeast used in fermentation defined by the first three discriminant functions (roots) obtained by forward stepwise linear discriminant analysis (SLDA) on the basis of the composition of phenolic compounds from grapes determined by UPLC/QqQ-MS/MS analysis.

Table S16. Variables from phenolic compounds included in the SLDA model with their contribution to the correct classification (%) of Malvazija istarska wines according to yeast used in fermentation.

| Phenolic compounds | Wine | | | | | | | |
| --- | --- | --- | --- | --- | --- | --- | --- | --- |
|  | SC | SC×SPx | TD+SC | MP+SC | PK+SC | LT+SC | SP+SC | TOT |
| 4-Aminobenzoic acid | 66.7 | 100.0 | 100.0 | 100.0 | 0.0 | 0.0 | 0.0 | 52.4 |
| Ferulic acid | 100.0 | 100.0 | 100.0 | 100.0 | 66.7 | 66.7 | 66.7 | 85.7 |
| Caffeic acid | 100.0 | 100.0 | 100.0 | 100.0 | 66.7 | 66.7 | 66.7 | 85.7 |
| Protocatechuic acid | 100.0 | 100.0 | 100.0 | 100.0 | 66.7 | 100.0 | 100.0 | 95.2 |
| Catechol | 100.0 | 100.0 | 100.0 | 100.0 | 100.0 | 100.0 | 100.0 | 100.0 |
| Kaempferol | 100.0 | 100.0 | 100.0 | 100.0 | 100.0 | 100.0 | 100.0 | 100.0 |
| Caftaric acid | 100.0 | 100.0 | 100.0 | 100.0 | 100.0 | 100.0 | 100.0 | 100.0 |
| Procyanidin B1 | 100.0 | 100.0 | 100.0 | 100.0 | 100.0 | 100.0 | 100.0 | 100.0 |
| Quercetin | 100.0 | 100.0 | 100.0 | 100.0 | 100.0 | 100.0 | 100.0 | 100.0 |
| *t*-Resveratrol | 100.0 | 100.0 | 100.0 | 100.0 | 100.0 | 100.0 | 100.0 | 100.0 |
| Procyanidin B2 + B4 | 100.0 | 100.0 | 100.0 | 100.0 | 100.0 | 100.0 | 100.0 | 100.0 |
| Syringic acid | 100.0 | 100.0 | 100.0 | 100.0 | 100.0 | 100.0 | 100.0 | 100.0 |
| *p*-Hydroxybenzoic acid | 100.0 | 100.0 | 100.0 | 100.0 | 100.0 | 100.0 | 100.0 | 100.0 |

Abbreviations: SC – *Saccharomyces cerevisiae*, SC×SPx – *Saccharomyces cerevisiae*×*Saccharomyces paradoxus* hybrid, TD+SC – *Torulaspora delbrueckii+S. cerevisiae*, MP+SC – *Metschnikowia pulcherrima+S. cerevisiae*, PK+SC – *Pichia kluyveri+S. cerevisiae,* LT+SC – *Lachancea thermotolerans+S. cerevisiae,* SP+SC – *Schizosaccharomyces pombe+S. cerevisiae*, TOT – TOTAL correct classification.

Table S17. Squared Mahalanobis distances of wine samples fermented with different yeasts from group centroids in the full discriminant space, obtained by forward stepwise linear discriminant analysis (SLDA) based on terpenoid composition determined by GC/MS and GC×GC/TOF-MS

| Squared Mahalanobis distances  from group centroids | Wine | | | | | | |
| --- | --- | --- | --- | --- | --- | --- | --- |
|  | SC | SC×SPx | TD+SC | MP+SC | PK+SC | LT+SC | SP+SC |
| SC_1 | 7 | 25235 | 16874 | 4842 | 616 | 5318 | 5641 |
| SC_2 | 8 | 24771 | 16642 | 4736 | 519 | 5168 | 5471 |
| SC_3 | 9 | 24970 | 16741 | 4777 | 573 | 5260 | 5565 |
| SC×SPx_1 | 24859 | 9 | 2000 | 8814 | 18328 | 46150 | 9713 |
| SC×SPx_2 | 25405 | 9 | 2044 | 9093 | 18799 | 47032 | 9965 |
| SC×SPx_3 | 24715 | 8 | 2004 | 8751 | 18198 | 45936 | 9618 |
| TD+SC_1 | 16845 | 1939 | 7 | 3895 | 11656 | 37464 | 4328 |
| TD+SC_2 | 16235 | 2081 | 8 | 3615 | 11170 | 36608 | 4005 |
| TD+SC_3 | 17178 | 2027 | 9 | 4078 | 11961 | 38026 | 4330 |
| MP+SC_1 | 4598 | 9171 | 3977 | 9 | 2148 | 18173 | 676 |
| MP+SC_2 | 4916 | 8782 | 3799 | 9 | 2365 | 18725 | 850 |
| MP+SC_3 | 4843 | 8706 | 3813 | 8 | 2295 | 18467 | 707 |
| PK+SC_1 | 535 | 18473 | 11635 | 2274 | 8 | 7985 | 2970 |
| PK+SC_2 | 601 | 18246 | 11426 | 2209 | 8 | 8023 | 2941 |
| PK+SC_3 | 572 | 18604 | 11725 | 2322 | 7 | 7804 | 2989 |
| LT+SC_1 | 5262 | 46313 | 37390 | 18479 | 7953 | 4 | 19325 |
| LT+SC_2 | 5200 | 46520 | 37390 | 18405 | 7891 | 8 | 19224 |
| LT+SC_3 | 5279 | 46279 | 37313 | 18475 | 7966 | 8 | 19338 |
| SP+SC_1 | 5611 | 9737 | 4205 | 738 | 3005 | 19423 | 8 |
| SP+SC_2 | 5500 | 3756 | 4211 | 739 | 2919 | 19152 | 8 |
| SP+SC_3 | 5567 | 9802 | 4247 | 756 | 2978 | 19317 | 9 |
| Total | 173744 | 325437 | 227462 | 117025 | 131365 | 404049 | 127683 |

Abbreviations: SC – *Saccharomyces cerevisiae* (control, monoculture); SC×SPx – *Saccharomyces cerevisiae*×*Saccharomyces paradoxus* hybrid (monoculture); TD+SC – *Torulaspora delbrueckii+S. cerevisiae*; MP+SC – *Metschnikowia pulcherrima+S. cerevisiae*; PK+SC – *Pichia kluyveri+S. cerevisiae*; LT+SC – *Lachancea thermotolerans+S. cerevisiae*; SP+SC – *Schizosaccharomyces pombe+S. cerevisiae* (TD+SC, MP+SC, PK+SC, LT+SC and SP+SC sequential fermentations were finished by *S. cerevisiae* (SC) inoculated at 2 vol. % ethanol).

Table S18. Squared Mahalanobis distances of wine samples fermented with different yeasts from group centroids in the full discriminant space, obtained by forward stepwise linear discriminant analysis (SLDA) based on thiol composition determined by GC/MS

| Squared Mahalanobis distances  from group centroids | Wine | | | | | | |
| --- | --- | --- | --- | --- | --- | --- | --- |
|  | SC | SC×SPx | TD+SC | MP+SC | PK+SC | LT+SC | SP+SC |
| SC_1 | 0 | 7 | 113 | 10 | 9 | 24 | 12 |
| SC_2 | 0 | 12 | 106 | 10 | 16 | 18 | 15 |
| SC_3 | 0 | 10 | 109 | 10 | 13 | 21 | 14 |
| SC×SPx_1 | 25 | 5 | 128 | 21 | 5 | 62 | 10 |
| SC×SPx_2 | 5 | 2 | 81 | 2 | 11 | 19 | 2 |
| SC×SPx_3 | 6 | 1 | 96 | 5 | 6 | 27 | 3 |
| TD+SC_1 | 96 | 91 | 1 | 51 | 137 | 33 | 66 |
| TD+SC_2 | 171 | 151 | 8 | 104 | 210 | 87 | 116 |
| TD+SC_3 | 72 | 68 | 4 | 34 | 108 | 21 | 47 |
| MP+SC_1 | 12 | 17 | 48 | 3 | 35 | 3 | 10 |
| MP+SC_2 | 16 | 3 | 68 | 3 | 16 | 24 | 0 |
| MP+SC_3 | 7 | 5 | 65 | 0 | 18 | 12 | 2 |
| PK+SC_1 | 16 | 6 | 152 | 24 | 0 | 62 | 15 |
| PK+SC_2 | 12 | 6 | 155 | 24 | 0 | 58 | 16 |
| PK+SC_3 | 9 | 3 | 137 | 17 | 0 | 47 | 11 |
| LT+SC_1 | 24 | 36 | 37 | 12 | 60 | 0 | 25 |
| LT+SC_2 | 15 | 28 | 52 | 9 | 47 | 1 | 21 |
| LT+SC_3 | 24 | 38 | 39 | 14 | 61 | 0 | 27 |
| SP+SC_1 | 16 | 4 | 66 | 2 | 17 | 24 | 0 |
| SP+SC_2 | 15 | 2 | 77 | 4 | 13 | 29 | 0 |
| SP+SC_3 | 10 | 2 | 74 | 2 | 13 | 21 | 0 |
| Total | 554 | 497 | 1615 | 359 | 794 | 592 | 413 |

Abbreviations: SC – *Saccharomyces cerevisiae* (control, monoculture); SC×SPx – *Saccharomyces cerevisiae*×*Saccharomyces paradoxus* hybrid (monoculture); TD+SC – *Torulaspora delbrueckii+S. cerevisiae*; MP+SC – *Metschnikowia pulcherrima+S. cerevisiae*; PK+SC – *Pichia kluyveri+S. cerevisiae*; LT+SC – *Lachancea thermotolerans+S. cerevisiae*; SP+SC – *Schizosaccharomyces pombe+S. cerevisiae* (TD+SC, MP+SC, PK+SC, LT+SC and SP+SC sequential fermentations were finished by *S. cerevisiae* (SC) inoculated at 2 vol. % ethanol).

Table S19. Squared Mahalanobis distances of wine samples fermented with different yeasts from group centroids in the full discriminant space, obtained by forward stepwise linear discriminant analysis (SLDA) based on acetate ester composition determined by GC/FID, GC/MS and GC×GC/TOF-MS

| Squared Mahalanobis distances  from group centroids | Wine | | | | | | |
| --- | --- | --- | --- | --- | --- | --- | --- |
|  | SC | SC×SPx | TD+SC | MP+SC | PK+SC | LT+SC | SP+SC |
| SC_1 | 7 | 69803 | 4300415 | 92954 | 108245 | 267086 | 1001610 |
| SC_2 | 7 | 70676 | 4306413 | 94095 | 109475 | 268750 | 1004704 |
| SC_3 | 7 | 71016 | 4308369 | 94580 | 109960 | 269293 | 1005808 |
| SC×SPx_1 | 70645 | 9 | 3281598 | 3152 | 5621 | 64201 | 543866 |
| SC×SPx_2 | 71302 | 7 | 3276851 | 2916 | 5352 | 63452 | 541854 |
| SC×SPx_3 | 69547 | 6 | 3288519 | 3159 | 5719 | 65083 | 546626 |
| TD+SC_1 | 4302413 | 3279960 | 9 | 3156421 | 3071771 | 2428600 | 1155880 |
| TD+SC_2 | 4307461 | 3284487 | 9 | 3160836 | 3076097 | 2432503 | 1158670 |
| TD+SC_3 | 4305329 | 3282527 | 9 | 3158880 | 3074228 | 2430795 | 1157381 |
| MP+SC_1 | 93571 | 2997 | 3160155 | 9 | 846 | 48750 | 493481 |
| MP+SC_2 | 94005 | 3165 | 3158581 | 8 | 885 | 48688 | 492749 |
| MP+SC_3 | 94056 | 3069 | 3157399 | 9 | 847 | 48441 | 492308 |
| PK+SC_1 | 109654 | 5567 | 3070608 | 875 | 7 | 38226 | 458691 |
| PK+SC_2 | 108828 | 5588 | 3077774 | 869 | 9 | 39144 | 461425 |
| PK+SC_3 | 109203 | 5540 | 3073713 | 833 | 9 | 38619 | 459856 |
| LT+SC_1 | 269260 | 64693 | 2427688 | 49123 | 39093 | 7 | 234228 |
| LT+SC_2 | 268796 | 6440 | 2429826 | 48762 | 38766 | 6 | 234873 |
| LT+SC_3 | 267073 | 63604 | 2434378 | 47991 | 38128 | 8 | 236244 |
| SP+SC_1 | 1005916 | 545523 | 1155248 | 494148 | 461206 | 236019 | 8 |
| SP+SC_2 | 1003261 | 543557 | 1158074 | 492364 | 459552 | 234762 | 9 |
| SP+SC_3 | 1002949 | 543270 | 1158607 | 492025 | 459213 | 234568 | 8 |
| Total | 17553290 | 11851504 | 52224243 | 11394009 | 11065029 | 9257001 | 11680279 |

Abbreviations: SC – *Saccharomyces cerevisiae* (control, monoculture); SC×SPx – *Saccharomyces cerevisiae*×*Saccharomyces paradoxus* hybrid (monoculture); TD+SC – *Torulaspora delbrueckii+S. cerevisiae*; MP+SC – *Metschnikowia pulcherrima+S. cerevisiae*; PK+SC – *Pichia kluyveri+S. cerevisiae*; LT+SC – *Lachancea thermotolerans+S. cerevisiae*; SP+SC – *Schizosaccharomyces pombe+S. cerevisiae* (TD+SC, MP+SC, PK+SC, LT+SC and SP+SC sequential fermentations were finished by *S. cerevisiae* (SC) inoculated at 2 vol. % ethanol).

Table S20. Squared Mahalanobis distances of wine samples fermented with different yeasts from group centroids in the full discriminant space, obtained by forward stepwise linear discriminant analysis (SLDA) based on aldehyde composition determined by GC/FID and GC×GC/TOF-MS

| Squared Mahalanobis distances  from group centroids | Wine | | | | | | |
| --- | --- | --- | --- | --- | --- | --- | --- |
|  | SC | SC×SPx | TD+SC | MP+SC | PK+SC | LT+SC | SP+SC |
| SC_1 | 3 | 140 | 103 | 27 | 46 | 47 | 325 |
| SC_2 | 6 | 164 | 135 | 28 | 76 | 73 | 371 |
| SC_3 | 3 | 151 | 135 | 44 | 59 | 50 | 322 |
| SC×SPx_1 | 151 | 7 | 105 | 156 | 46 | 65 | 169 |
| SC×SPx_2 | 134 | 6 | 64 | 121 | 31 | 61 | 177 |
| SC×SPx_3 | 179 | 9 | 83 | 175 | 54 | 79 | 194 |
| TD+SC_1 | 143 | 105 | 8 | 115 | 56 | 104 | 318 |
| TD+SC_2 | 118 | 62 | 7 | 81 | 30 | 66 | 270 |
| TD+SC_3 | 120 | 84 | 6 | 96 | 43 | 87 | 293 |
| MP+SC_1 | 27 | 133 | 101 | 6 | 54 | 57 | 309 |
| MP+SC_2 | 24 | 142 | 81 | 3 | 56 | 70 | 368 |
| MP+SC_3 | 52 | 171 | 105 | 6 | 77 | 90 | 393 |
| PK+SC_1 | 91 | 26 | 40 | 91 | 7 | 25 | 147 |
| PK+SC_2 | 60 | 50 | 32 | 44 | 5 | 16 | 188 |
| PK+SC_3 | 37 | 53 | 55 | 55 | 7 | 16 | 224 |
| LT+SC_1 | 35 | 55 | 65 | 51 | 6 | 4 | 181 |
| LT+SC_2 | 72 | 73 | 98 | 87 | 23 | 5 | 114 |
| LT+SC_3 | 65 | 71 | 88 | 78 | 25 | 6 | 150 |
| SP+SC_1 | 347 | 205 | 293 | 369 | 194 | 152 | 8 |
| SP+SC_2 | 348 | 141 | 274 | 357 | 174 | 149 | 7 |
| SP+SC_3 | 332 | 195 | 316 | 350 | 194 | 151 | 7 |
| Total | 2347 | 2041 | 2194 | 2340 | 1262 | 1375 | 4535 |

Abbreviations: SC – *Saccharomyces cerevisiae* (control, monoculture); SC×SPx – *Saccharomyces cerevisiae*×*Saccharomyces paradoxus* hybrid (monoculture); TD+SC – *Torulaspora delbrueckii+S. cerevisiae*; MP+SC – *Metschnikowia pulcherrima+S. cerevisiae*; PK+SC – *Pichia kluyveri+S. cerevisiae*; LT+SC – *Lachancea thermotolerans+S. cerevisiae*; SP+SC – *Schizosaccharomyces pombe+S. cerevisiae* (TD+SC, MP+SC, PK+SC, LT+SC and SP+SC sequential fermentations were finished by *S. cerevisiae* (SC) inoculated at 2 vol. % ethanol).

Table S21. Squared Mahalanobis distances of wine samples fermented with different yeasts from group centroids in the full discriminant space, obtained by forward stepwise linear discriminant analysis (SLDA) based on norisoprenoid composition determined by GC/MS and GC×GC/TOF-MS

| Squared Mahalanobis distances  from group centroids | Wine | | | | | | |
| --- | --- | --- | --- | --- | --- | --- | --- |
|  | SC | SC×SPx | TD+SC | MP+SC | PK+SC | LT+SC | SP+SC |
| SC_1 | 6 | 166 | 17 | 13 | 16 | 50 | 29 |
| SC_2 | 5 | 123 | 2 | 18 | 24 | 45 | 37 |
| SC_3 | 2 | 123 | 5 | 11 | 7 | 28 | 29 |
| SC×SPx_1 | 164 | 4 | 131 | 159 | 160 | 140 | 117 |
| SC×SPx_2 | 140 | 1 | 108 | 138 | 138 | 120 | 100 |
| SC×SPx_3 | 105 | 6 | 78 | 105 | 96 | 75 | 84 |
| TD+SC_1 | 11 | 93 | 5 | 22 | 20 | 32 | 25 |
| TD+SC_2 | 9 | 101 | 3 | 21 | 28 | 46 | 37 |
| TD+SC_3 | 1 | 123 | 2 | 9 | 13 | 33 | 24 |
| MP+SC_1 | 6 | 130 | 9 | 3 | 9 | 29 | 11 |
| MP+SC_2 | 14 | 139 | 20 | 2 | 9 | 33 | 16 |
| MP+SC_3 | 16 | 129 | 19 | 2 | 9 | 22 | 13 |
| PK+SC_1 | 18 | 144 | 25 | 15 | 3 | 12 | 39 |
| PK+SC_2 | 8 | 156 | 16 | 3 | 6 | 29 | 18 |
| PK+SC_3 | 21 | 97 | 23 | 17 | 5 | 10 | 22 |
| LT+SC_1 | 39 | 125 | 39 | 31 | 13 | 1 | 43 |
| LT+SC_2 | 31 | 104 | 27 | 19 | 11 | 3 | 26 |
| LT+SC_3 | 46 | 103 | 41 | 33 | 21 | 2 | 38 |
| SP+SC_1 | 13 | 95 | 13 | 13 | 20 | 41 | 8 |
| SP+SC_2 | 44 | 97 | 44 | 19 | 30 | 44 | 7 |
| SP+SC_3 | 46 | 121 | 43 | 23 | 37 | 37 | 7 |
| Total | 744 | 2180 | 671 | 676 | 675 | 832 | 731 |

Abbreviations: SC – *Saccharomyces cerevisiae* (control, monoculture); SC×SPx – *Saccharomyces cerevisiae*×*Saccharomyces paradoxus* hybrid (monoculture); TD+SC – *Torulaspora delbrueckii+S. cerevisiae*; MP+SC – *Metschnikowia pulcherrima+S. cerevisiae*; PK+SC – *Pichia kluyveri+S. cerevisiae*; LT+SC – *Lachancea thermotolerans+S. cerevisiae*; SP+SC – *Schizosaccharomyces pombe+S. cerevisiae* (TD+SC, MP+SC, PK+SC, LT+SC and SP+SC sequential fermentations were finished by *S. cerevisiae* (SC) inoculated at 2 vol. % ethanol).

Table S22. Squared Mahalanobis distances of wine samples fermented with different yeasts from group centroids in the full discriminant space, obtained by forward stepwise linear discriminant analysis (SLDA) based on benzenoid composition determined by GC/MS and GC×GC/TOF-MS

| Squared Mahalanobis distances  from group centroids | Wine | | | | | | |
| --- | --- | --- | --- | --- | --- | --- | --- |
|  | SC | SC×SPx | TD+SC | MP+SC | PK+SC | LT+SC | SP+SC |
| SC_1 | 8 | 49212 | 26527 | 11058 | 8793 | 15946 | 27376 |
| SC_2 | 9 | 48435 | 27087 | 11276 | 9213 | 16615 | 28238 |
| SC_3 | 8 | 47971 | 27009 | 11159 | 9227 | 16842 | 28348 |
| SC×SPx_1 | 48631 | 9 | 62117 | 37257 | 60347 | 107751 | 100223 |
| SC×SPx_2 | 48032 | 8 | 61870 | 36949 | 59919 | 107088 | 99822 |
| SC×SPx_3 | 48956 | 8 | 62417 | 37504 | 60692 | 108249 | 100664 |
| TD+SC_1 | 26880 | 61899 | 6 | 5185 | 5877 | 22515 | 7371 |
| TD+SC_2 | 26971 | 62669 | 7 | 5315 | 5845 | 22209 | 7122 |
| TD+SC_3 | 26769 | 61834 | 9 | 5155 | 5884 | 22519 | 7435 |
| MP+SC_1 | 11419 | 37655 | 4988 | 7 | 3262 | 22028 | 15135 |
| MP+SC_2 | 11235 | 37262 | 5147 | 8 | 3345 | 22149 | 15406 |
| MP+SC_3 | 10838 | 36793 | 5523 | 9 | 3357 | 22142 | 15726 |
| PK+SC_1 | 9263 | 59933 | 5606 | 3170 | 9 | 8691 | 6301 |
| PK+SC_2 | 8928 | 60580 | 6084 | 3429 | 8 | 8252 | 6340 |
| PK+SC_3 | 9043 | 60445 | 5920 | 3366 | 9 | 8342 | 6300 |
| LT+SC_1 | 16333 | 107478 | 22444 | 22052 | 8391 | 3 | 7512 |
| LT+SC_2 | 16661 | 108488 | 22805 | 22523 | 8694 | 7 | 7651 |
| LT+SC_3 | 16401 | 107114 | 21989 | 21737 | 8191 | 8 | 7234 |
| SP+SC_1 | 27911 | 100224 | 7311 | 15395 | 6284 | 7425 | 9 |
| SP+SC_2 | 27965 | 100226 | 7333 | 15437 | 6313 | 7448 | 9 |
| SP+SC_3 | 28087 | 100260 | 7289 | 15438 | 6346 | 7534 | 9 |
| Total | 420347 | 1248502 | 389489 | 283429 | 280005 | 553759 | 494230 |

Abbreviations: SC – *Saccharomyces cerevisiae* (control, monoculture); SC×SPx – *Saccharomyces cerevisiae*×*Saccharomyces paradoxus* hybrid (monoculture); TD+SC – *Torulaspora delbrueckii+S. cerevisiae*; MP+SC – *Metschnikowia pulcherrima+S. cerevisiae*; PK+SC – *Pichia kluyveri+S. cerevisiae*; LT+SC – *Lachancea thermotolerans+S. cerevisiae*; SP+SC – *Schizosaccharomyces pombe+S. cerevisiae* (TD+SC, MP+SC, PK+SC, LT+SC and SP+SC sequential fermentations were finished by *S. cerevisiae* (SC) inoculated at 2 vol. % ethanol).

Table S23. Squared Mahalanobis distances of wine samples fermented with different yeasts from group centroids in the full discriminant space, obtained by forward stepwise linear discriminant analysis (SLDA) based on ethyl ester composition determined by GC/MS and GC×GC/TOF-MS

| Squared Mahalanobis distances  from group centroids | Wine | | | | | | |
| --- | --- | --- | --- | --- | --- | --- | --- |
|  | SC | SC×SPx | TD+SC | MP+SC | PK+SC | LT+SC | SP+SC |
| SC_1 | 7 | 342443 | 172645 | 18136 | 9441 | 22235 | 7983 |
| SC_2 | 5 | 341434 | 171770 | 17860 | 9168 | 21921 | 7966 |
| SC_3 | 9 | 342135 | 172619 | 18132 | 9527 | 22258 | 8066 |
| SC×SPx_1 | 339654 | 9 | 33359 | 205798 | 257485 | 192511 | 435371 |
| SC×SPx_2 | 344537 | 9 | 34991 | 209601 | 261814 | 196213 | 440955 |
| SC×SPx_3 | 341826 | 8 | 34193 | 207607 | 259586 | 194263 | 438027 |
| TD+SC_1 | 172223 | 34341 | 7 | 80278 | 111050 | 71655 | 236934 |
| TD+SC_2 | 172054 | 34354 | 3 | 80150 | 110866 | 71554 | 236714 |
| TD+SC_3 | 172754 | 33839 | 7 | 80615 | 111482 | 72004 | 237690 |
| MP+SC_1 | 18062 | 207622 | 80272 | 8 | 3605 | 289 | 43024 |
| MP+SC_2 | 17988 | 207991 | 80577 | 9 | 3755 | 340 | 43072 |
| MP+SC_3 | 18085 | 207394 | 80204 | 9 | 3722 | 313 | 43243 |
| PK+SC_1 | 9411 | 259770 | 111183 | 3728 | 9 | 5357 | 23914 |
| PK+SC_2 | 9257 | 259370 | 111059 | 3624 | 8 | 5232 | 23973 |
| PK+SC_3 | 9474 | 259746 | 111165 | 3730 | 9 | 5369 | 23955 |
| LT+SC_1 | 21934 | 194464 | 71951 | 317 | 5374 | 8 | 49183 |
| LT+SC_2 | 21900 | 195342 | 72381 | 259 | 5158 | 9 | 48850 |
| LT+SC_3 | 22585 | 193181 | 70890 | 365 | 5426 | 8 | 49840 |
| SP+SC_1 | 8068 | 438872 | 237734 | 43364 | 24137 | 49571 | 9 |
| SP+SC_2 | 8081 | 438380 | 237319 | 43211 | 24008 | 49392 | 9 |
| SP+SC_3 | 7873 | 437102 | 236296 | 42767 | 23697 | 48911 | 9 |
| Total | 1715787 | 4427805 | 2120625 | 1059568 | 1239327 | 1029413 | 2398787 |

Abbreviations: SC – *Saccharomyces cerevisiae* (control, monoculture); SC×SPx – *Saccharomyces cerevisiae*×*Saccharomyces paradoxus* hybrid (monoculture); TD+SC – *Torulaspora delbrueckii+S. cerevisiae*; MP+SC – *Metschnikowia pulcherrima+S. cerevisiae*; PK+SC – *Pichia kluyveri+S. cerevisiae*; LT+SC – *Lachancea thermotolerans+S. cerevisiae*; SP+SC – *Schizosaccharomyces pombe+S. cerevisiae* (TD+SC, MP+SC, PK+SC, LT+SC and SP+SC sequential fermentations were finished by *S. cerevisiae* (SC) inoculated at 2 vol. % ethanol).

Table S24. Squared Mahalanobis distances of wine samples fermented with different yeasts from group centroids in the full discriminant space, obtained by forward stepwise linear discriminant analysis (SLDA) based on other ester composition determined by GC/MS and GC×GC/TOF-MS

| Squared Mahalanobis distances  from group centroids | Wine | | | | | | |
| --- | --- | --- | --- | --- | --- | --- | --- |
|  | SC | SC×SPx | TD+SC | MP+SC | PK+SC | LT+SC | SP+SC |
| SC_1 | 8 | 75537 | 14782 | 12993 | 20174 | 3562 | 5305 |
| SC_2 | 9 | 77094 | 15188 | 12250 | 19253 | 3193 | 5063 |
| SC_3 | 9 | 75229 | 14473 | 12702 | 19760 | 3291 | 4823 |
| SC×SPx_1 | 75899 | 8 | 29626 | 137594 | 154069 | 90129 | 90221 |
| SC×SPx_2 | 75816 | 7 | 29513 | 137587 | 154052 | 90009 | 90087 |
| SC×SPx_3 | 76143 | 9 | 30011 | 138369 | 155027 | 90636 | 90845 |
| TD+SC_1 | 14597 | 30031 | 8 | 41652 | 50429 | 17232 | 19370 |
| TD+SC_2 | 14990 | 29251 | 8 | 42617 | 51503 | 17862 | 19972 |
| TD+SC_3 | 14852 | 29868 | 6 | 42145 | 50930 | 17507 | 19676 |
| MP+SC_1 | 12518 | 137480 | 41929 | 9 | 934 | 5658 | 6652 |
| MP+SC_2 | 12666 | 137999 | 42216 | 8 | 934 | 5772 | 6761 |
| MP+SC_3 | 12759 | 138072 | 42271 | 7 | 893 | 5766 | 6777 |
| PK+SC_1 | 19764 | 154688 | 51133 | 912 | 9 | 9818 | 10231 |
| PK+SC_2 | 19527 | 153446 | 50329 | 893 | 7 | 9460 | 9997 |
| PK+SC_3 | 19895 | 155015 | 51402 | 956 | 8 | 9933 | 10242 |
| LT+SC_1 | 3387 | 90088 | 17428 | 5769 | 9772 | 5 | 2320 |
| LT+SC_2 | 3292 | 90622 | 17683 | 5683 | 9686 | 8 | 2252 |
| LT+SC_3 | 3362 | 90061 | 17488 | 5739 | 9748 | 8 | 2286 |
| SP+SC_1 | 4969 | 89838 | 19297 | 6738 | 10173 | 2157 | 9 |
| SP+SC_2 | 5131 | 90703 | 19867 | 6774 | 10188 | 2387 | 8 |
| SP+SC_3 | 5091 | 90614 | 19857 | 6679 | 10110 | 2319 | 9 |
| Total | 394683 | 1735661 | 524515 | 618076 | 737661 | 386711 | 402906 |

Abbreviations: SC – *Saccharomyces cerevisiae* (control, monoculture); SC×SPx – *Saccharomyces cerevisiae*×*Saccharomyces paradoxus* hybrid (monoculture); TD+SC – *Torulaspora delbrueckii+S. cerevisiae*; MP+SC – *Metschnikowia pulcherrima+S. cerevisiae*; PK+SC – *Pichia kluyveri+S. cerevisiae*; LT+SC – *Lachancea thermotolerans+S. cerevisiae*; SP+SC – *Schizosaccharomyces pombe+S. cerevisiae* (TD+SC, MP+SC, PK+SC, LT+SC and SP+SC sequential fermentations were finished by *S. cerevisiae* (SC) inoculated at 2 vol. % ethanol).

Table S25. Squared Mahalanobis distances of wine samples fermented with different yeasts from group centroids in the full discriminant space, obtained by forward stepwise linear discriminant analysis (SLDA) based on ketone composition determined by GC×GC/TOF-MS

| Squared Mahalanobis distances  from group centroids | Wine | | | | | | |
| --- | --- | --- | --- | --- | --- | --- | --- |
|  | SC | SC×SPx | TD+SC | MP+SC | PK+SC | LT+SC | SP+SC |
| SC_1 | 7 | 340 | 3918 | 2177 | 2261 | 1984 | 1297 |
| SC_2 | 4 | 267 | 4188 | 2348 | 2441 | 2142 | 1417 |
| SC_3 | 8 | 203 | 3801 | 2036 | 2129 | 1843 | 1175 |
| SC×SPx_1 | 293 | 6 | 4170 | 2237 | 2388 | 2035 | 1249 |
| SC×SPx_2 | 257 | 1 | 4014 | 2127 | 2263 | 1920 | 1180 |
| SC×SPx_3 | 252 | 4 | 4141 | 2215 | 2352 | 2002 | 1258 |
| TD+SC_1 | 3969 | 4123 | 6 | 327 | 274 | 404 | 890 |
| TD+SC_2 | 3973 | 4091 | 6 | 210 | 266 | 391 | 870 |
| TD+SC_3 | 3965 | 4119 | 7 | 325 | 276 | 406 | 883 |
| MP+SC_1 | 2181 | 2178 | 320 | 5 | 18 | 14 | 155 |
| MP+SC_2 | 2165 | 2178 | 315 | 1 | 8 | 6 | 167 |
| MP+SC_3 | 2209 | 2224 | 322 | 6 | 14 | 18 | 194 |
| PK+SC_1 | 2247 | 2297 | 274 | 7 | 9 | 16 | 205 |
| PK+SC_2 | 2344 | 2401 | 253 | 18 | 5 | 30 | 246 |
| PK+SC_3 | 2242 | 2315 | 292 | 25 | 7 | 26 | 238 |
| LT+SC_1 | 1953 | 1934 | 418 | 13 | 25 | 3 | 111 |
| LT+SC_2 | 1929 | 1905 | 430 | 20 | 29 | 5 | 117 |
| LT+SC_3 | 2082 | 2120 | 348 | 6 | 9 | 5 | 169 |
| SP+SC_1 | 1286 | 1206 | 883 | 178 | 229 | 131 | 5 |
| SP+SC_2 | 1184 | 1138 | 970 | 202 | 263 | 161 | 8 |
| SP+SC_3 | 1417 | 1349 | 788 | 142 | 194 | 110 | 5 |
| Total | 35967 | 36399 | 29863 | 14625 | 15461 | 13651 | 11839 |

Abbreviations: SC – *Saccharomyces cerevisiae* (control, monoculture); SC×SPx – *Saccharomyces cerevisiae*×*Saccharomyces paradoxus* hybrid (monoculture); TD+SC – *Torulaspora delbrueckii+S. cerevisiae*; MP+SC – *Metschnikowia pulcherrima+S. cerevisiae*; PK+SC – *Pichia kluyveri+S. cerevisiae*; LT+SC – *Lachancea thermotolerans+S. cerevisiae*; SP+SC – *Schizosaccharomyces pombe+S. cerevisiae* (TD+SC, MP+SC, PK+SC, LT+SC and SP+SC sequential fermentations were finished by *S. cerevisiae* (SC) inoculated at 2 vol. % ethanol).

Table S26. Squared Mahalanobis distances of wine samples fermented with different yeasts from group centroids in the full discriminant space, obtained by forward stepwise linear discriminant analysis (SLDA) based on alcohol composition determined by GC/FID, GC/MS, and GC×GC/TOF-MS

| Squared Mahalanobis distances  from group centroids | Wine | | | | | | |
| --- | --- | --- | --- | --- | --- | --- | --- |
|  | SC | SC×SPx | TD+SC | MP+SC | PK+SC | LT+SC | SP+SC |
| SC_1 | 7 | 2494 | 10400 | 13439 | 12069 | 16925 | 15597 |
| SC_2 | 6 | 2590 | 10337 | 13531 | 12058 | 16814 | 15659 |
| SC_3 | 4 | 2639 | 10060 | 13136 | 11712 | 16402 | 15264 |
| SC×SPx_1 | 2590 | 7 | 11510 | 12221 | 11905 | 18649 | 14096 |
| SC×SPx_2 | 2569 | 7 | 11467 | 12081 | 11753 | 18521 | 13879 |
| SC×SPx_3 | 2567 | 5 | 11555 | 12187 | 11885 | 18627 | 14061 |
| TD+SC_1 | 10296 | 11460 | 8 | 1169 | 518 | 1055 | 1507 |
| TD+SC_2 | 10216 | 11679 | 7 | 1343 | 605 | 1073 | 1653 |
| TD+SC_3 | 10288 | 11394 | 5 | 112 | 451 | 1100 | 1403 |
| MP+SC_1 | 13350 | 12204 | 1180 | 7 | 304 | 1864 | 280 |
| MP+SC_2 | 13340 | 12058 | 1209 | 6 | 301 | 1903 | 262 |
| MP+SC_3 | 13421 | 12230 | 1237 | 9 | 280 | 1829 | 277 |
| PK+SC_1 | 11649 | 11594 | 521 | 274 | 8 | 1222 | 485 |
| PK+SC_2 | 12473 | 12280 | 571 | 265 | 8 | 1144 | 408 |
| PK+SC_3 | 11725 | 11672 | 487 | 348 | 8 | 1196 | 522 |
| LT+SC_1 | 16340 | 18141 | 973 | 1726 | 1050 | 7 | 1577 |
| LT+SC_2 | 16854 | 18756 | 1118 | 1955 | 1256 | 5 | 1718 |
| LT+SC_3 | 16947 | 18898 | 1134 | 1911 | 1249 | 5 | 1732 |
| SP+SC_1 | 15318 | 13882 | 1487 | 251 | 436 | 1683 | 8 |
| SP+SC_2 | 15545 | 14117 | 1489 | 251 | 439 | 1615 | 6 |
| SP+SC_3 | 15661 | 14038 | 1587 | 316 | 535 | 1733 | 6 |
| Total | 211166 | 212144 | 78345 | 86538 | 78830 | 123372 | 100399 |

Abbreviations: SC – *Saccharomyces cerevisiae* (control, monoculture); SC×SPx – *Saccharomyces cerevisiae*×*Saccharomyces paradoxus* hybrid (monoculture); TD+SC – *Torulaspora delbrueckii+S. cerevisiae*; MP+SC – *Metschnikowia pulcherrima+S. cerevisiae*; PK+SC – *Pichia kluyveri+S. cerevisiae*; LT+SC – *Lachancea thermotolerans+S. cerevisiae*; SP+SC – *Schizosaccharomyces pombe+S. cerevisiae* (TD+SC, MP+SC, PK+SC, LT+SC and SP+SC sequential fermentations were finished by *S. cerevisiae* (SC) inoculated at 2 vol. % ethanol).

Table S27. Squared Mahalanobis distances of wine samples fermented with different yeasts from group centroids in the full discriminant space, obtained by forward stepwise linear discriminant analysis (SLDA) based on volatile phenol composition determined by GC×GC/TOF-MS

| Squared Mahalanobis distances  from group centroids | Wine | | | | | | |
| --- | --- | --- | --- | --- | --- | --- | --- |
|  | SC | SC×SPx | TD+SC | MP+SC | PK+SC | LT+SC | SP+SC |
| SC_1 | 7 | 36 | 144 | 137 | 148 | 84 | 102 |
| SC_2 | 3 | 13 | 118 | 99 | 127 | 59 | 79 |
| SC_3 | 3 | 24 | 85 | 67 | 92 | 40 | 49 |
| SC×SPx_1 | 17 | 4 | 164 | 131 | 161 | 87 | 108 |
| SC×SPx_2 | 26 | 2 | 124 | 115 | 118 | 55 | 92 |
| SC×SPx_3 | 23 | 1 | 137 | 129 | 128 | 63 | 100 |
| TD+SC_1 | 146 | 177 | 8 | 62 | 35 | 37 | 59 |
| TD+SC_2 | 107 | 142 | 6 | 41 | 20 | 18 | 35 |
| TD+SC_3 | 99 | 118 | 4 | 19 | 10 | 12 | 15 |
| MP+SC_1 | 93 | 109 | 28 | 5 | 23 | 24 | 8 |
| MP+SC_2 | 96 | 129 | 40 | 3 | 51 | 46 | 13 |
| MP+SC_3 | 113 | 143 | 48 | 4 | 41 | 53 | 8 |
| PK+SC_1 | 93 | 109 | 8 | 31 | 6 | 7 | 20 |
| PK+SC_2 | 132 | 152 | 19 | 29 | 2 | 27 | 20 |
| PK+SC_3 | 139 | 151 | 32 | 53 | 4 | 31 | 37 |
| LT+SC_1 | 60 | 82 | 11 | 31 | 16 | 2 | 19 |
| LT+SC_2 | 63 | 70 | 17 | 52 | 21 | 2 | 36 |
| LT+SC_3 | 55 | 52 | 27 | 35 | 23 | 3 | 23 |
| SP+SC_1 | 83 | 100 | 33 | 13 | 25 | 25 | 6 |
| SP+SC_2 | 53 | 75 | 43 | 15 | 33 | 26 | 7 |
| SP+SC_3 | 97 | 135 | 32 | 5 | 25 | 37 | 4 |
| Total | 1509 | 1823 | 1127 | 1076 | 1107 | 737 | 840 |

Abbreviations: SC – *Saccharomyces cerevisiae* (control, monoculture); SC×SPx – *Saccharomyces cerevisiae*×*Saccharomyces paradoxus* hybrid (monoculture); TD+SC – *Torulaspora delbrueckii+S. cerevisiae*; MP+SC – *Metschnikowia pulcherrima+S. cerevisiae*; PK+SC – *Pichia kluyveri+S. cerevisiae*; LT+SC – *Lachancea thermotolerans+S. cerevisiae*; SP+SC – *Schizosaccharomyces pombe+S. cerevisiae* (TD+SC, MP+SC, PK+SC, LT+SC and SP+SC sequential fermentations were finished by *S. cerevisiae* (SC) inoculated at 2 vol. % ethanol).
